# NLRP3/Caspase-1-Mediated Pyroptosis of Astrocytes Induced by Antipsychotics Is Inhibited by a Histamine H1 Receptor-Selective Agonist

**DOI:** 10.3389/fnagi.2022.847561

**Published:** 2022-05-09

**Authors:** Meng He, Jun Fan, Ruqin Zhou, Guanbin Gao, Ruoxi Li, YuFeng Zuo, Benben Li, Yanmei Li, Taolei Sun

**Affiliations:** ^1^School of Chemistry, Chemical Engineering and Life Sciences, Wuhan University of Technology, Wuhan, China; ^2^State Key Laboratory of Advanced Technology for Materials Synthesis and Processing, Wuhan University of Technology, Wuhan, China; ^3^School of Public Health, Tongji Medical College, Huazhong University of Science and Technology, Wuhan, China; ^4^Hubei Provincial Key Laboratory for Applied Toxicology, Hubei Provincial Center for Disease Control and Prevention, Wuhan, China

**Keywords:** antipsychotics, astrocytes, brain volume loss, pyroptosis, NLRP3, caspase-1, GSDMD

## Abstract

Emerging data indicate that antipsychotic treatment causes brain volume loss and astrocyte death, but the mechanisms remain elusive. Pyroptosis, inflammatory cell death characterized by the formation of inflammatory bodies, increased expression of nod-like receptor proteins (NLRPs) such as NLRP3, and activation of caspases and gasdermin D (GSDMD) are largely associated with innate immunity, inflammation, and cell injury/death. However, the main effect of antipsychotics on astrocyte pyroptotic signaling and the molecular mechanisms remain obscure. In the present study, 72-h treatment with olanzapine, quetiapine, risperidone, or haloperidol significantly decreased the viability of astrocytes. Twenty-four hour treatment with olanzapine, quetiapine, risperidone, or haloperidol dose-dependently increased the protein expression of astrocytic NLRP3, NLRP6, caspase-1, caspase-4, and GSDMD. Co-treatment with a histamine H1 receptor agonist, 2-(3-trifluoromethylphenyl) histamine (FMPH), dose-dependently reduced the increased expression of NLRP3, caspase-1 and GSDMD induced by olanzapine, quetiapine, risperidone, or haloperidol. Moreover, olanzapine, quetiapine, risperidone, or haloperidol treatment induced pore formation in the membranes of astrocytes, and these effects were inhibited by FMPH co-treatment. Taken together, antipsychotic treatment activated astrocyte pyroptotic signaling, and these effects may be related to antipsychotic-induced astrocyte death. H1 receptor activation is an effective treatment strategy to suppress antipsychotic-induced astrocyte pyroptosis and inflammation.

## Introduction

Antipsychotics, in particular olanzapine, risperidone, quetiapine, and haloperidol, are widely used in the treatment of psychotic disorders. However, numerous clinical studies have revealed that patients who receive olanzapine, quetiapine, risperidone, or haloperidol treatment present a significant reduction in brain size, cortical thinning, and a decrease in cortical volume in the frontal and parietal regions (Miller et al., 2001; Molina et al., 2007; Moncrieff and Leo, 2010; Ebdrup et al., 2011; Ho et al., 2011). Consistently with the clinical findings, animal studies have revealed that chronic olanzapine or haloperidol treatment significantly decreases whole-brain volume, particularly in the cortex (Konopaske et al., 2008; Vernon et al., 2011, 2012). Although much effort has been made to determine the mechanisms by which antipsychotics induce cell death (Vucicevic et al., 2014; Boz et al., 2020), the molecular pathways remain elusive.

Astrocytes are the most abundant cell type in the central nervous system (CNS) and have attracted increasing attention in the regulation of neuronal development and function, metabolism, brain injury, and inflammation (Horvath et al., 2010). Chronic olanzapine or haloperidol treatment induced marked astrocyte loss in the monkey parietal cortex (Konopaske et al., 2008). Haloperidol treatment decreased astrocytic markers including glial fibrillary acidic protein (GFAP) and connexin 43 (CX43) in the rat prefrontal cortex (Fatemi et al., 2008). Olanzapine treatment for 3 days induced cell death in cultured astrocytes (He et al., 2020). However, the mechanisms by which antipsychotics induce astrocyte death are unclear.

Pyroptosis has received increased attention due to its involvement in the pathogenesis of various severe diseases such as Alzheimer’s disease, Parkinson’s disease, atherosclerosis, cancer, osteoarthritis, and stroke (An et al., 2020; Cai et al., 2021; Li et al., 2021b; Liu et al., 2021; Wang et al., 2021; Romero et al., 2022). Pyroptosis is a proinflammatory programmed cell death, characterized by the formation of inflammatory bodies, the activation of caspases, the formation of membrane pores, and the release of proinflammatory factors such as interleukin 1β (IL-1β) (Yang et al., 2015; Wu et al., 2019; Ma et al., 2021). Canonical and non-canonical inflammasome activation pathways contribute to pyroptosis (Ma et al., 2021). In the canonical pathway, the inflammasome sensors, nod-like receptor proteins (NLRPs) such as NLRP3 and NLRP6, form inflammasome complexes, leading to activation of caspase-1 (McIlwain et al., 2013). Activated caspase-1 cleaves the inhibitory C-terminal domain of gasdermin D (GSDMD), thus helping to liberate the N-terminal domain of GSDMD. N-GSDMD accumulates inside the cell membrane to form non-selective holes, resulting in cell pyroptosis (Yang et al., 2020). Caspase-1 could also promote the maturation and release of proinflammatory factors including IL-1β, IL-6 and IL-18, inducing an inflammatory response and pyroptosis (Ghimire et al., 2020; Zhang et al., 2020; Yu et al., 2021). In addition to NLRPs, NLR family caspase recruitment domain containing 4 (NLRC4) protein could form the NLRC4 inflammasome to activate caspase-1, resulting in pyroptosis (Franchi et al., 2009; McClellan et al., 2017). In the non-canonical pyroptosis pathway, the upstream sensory complexes of human caspase-4 are absent, and caspase-4 could bind directly to cellular lipopolysaccharide (LPS), which activates caspase-4 (Yu et al., 2021). Activated caspase-4 cleaves GSDMD into N-GSDMD in a manner similar to caspase-1, leading to pore formation and pyroptosis. Moreover, caspase-4 could induce the maturation and secretion of IL-1β *via* NLRP3/caspase-1 signaling, thereby inducing pyroptosis (Yu et al., 2021).

In pyroptotic signaling, NLRP3 is the most widely studied NLRP and has been reported to be related to various inflammatory diseases (Yang et al., 2021; Yu et al., 2021). Activation of NLRP3 induces the release of IL-1β in the CNS (Cai et al., 2021; Tastan et al., 2021), while inhibition of NLRP3 stops IL-1β production, reduces pyroptosis in the CNS, and improves cognitive function (Cai et al., 2021; Tastan et al., 2021). Moreover, the NLRP3 inflammasome is expressed by astrocytes and is involved in astrocyte dysfunction and the inflammatory response (Johann et al., 2015; Zhu and Tang, 2020). In primary cultured astrocytes, activation of the NLRP3 inflammasome *via* LPS plus adenosine triphosphate markedly increased the expression of caspase-1 and IL-1β (Zhu et al., 2018). Our previous study has reported that in both cultured astrocytes and the rat prefrontal cortex, olanzapine increased the expression of IL-1β (Li et al., 2021c). Haloperidol increased IL-β in the rat brain (Sonego et al., 2021; Mezzomo et al., 2022). These findings suggest that antipsychotics such as olanzapine and haloperidol might significantly affect the NLRP3/caspase-1 signaling, leading to IL-1β production and astrocyte death. To understand the mechanisms by which antipsychotics cause cell death and brain volume loss, we examined how different antipsychotics, including olanzapine, quetiapine, risperidone and haloperidol affect cell growth; NLRP3/caspase-1 signaling; and NLRP6, NLRC4 and caspase-4 expression in cultured astrocytes.

Moreover, it has been reported that histamine inhibited the secretion of IL-1β, a phenomenon completely abolished by co-treatment with a histamine H1 receptor antagonist, cetirizine, in primary cultured astrocytes (Xu et al., 2018). These findings suggest that astrocyte inflammation may be largely related to H1 receptor antagonism (Xu et al., 2018). However, whether the H1 receptor is related to antipsychotic-induced changes in NLRP3/caspase-1 signaling is unclear. We previously reported that olanzapine treatment induced H1 receptor signaling dysfunction in the rat brain, and this effect was inhibited by using a selective H1 receptor agonist, namely 2-(3-trifluoromethylphenyl) histamine (FMPH) (He et al., 2014; Chen et al., 2020b). Therefore, we investigated whether activation of the H1 receptor *via* FMPH could inhibit antipsychotic-induced changes in NLRP3/caspase-1 signaling. This study should help to further understand antipsychotic-induced side effects including central inflammation, astrocyte death, and brain volume loss during antipsychotic treatment, and it should provide valuable information to develop effective treatment strategies and novel antipsychotics with fewer side effects.

## Materials and Methods

### Cell Line and Antipsychotics

A human astrocyte cell line (C1028) was purchased from Shanghai Honsun Biological Technology Co., Ltd. (Shanghai, China). These astrocytes originated from the cortex of the human brain, and therefore should reflect the function of cortical astrocytes (He et al., 2020). Astrocytes were cultured in Dulbecco’s Modified Eagle Medium (DMEM) (Thermo Fisher Scientific, Wuhan, China) with 10% fetal bovine serum and 1% penicillin/streptomycin (Gibco Life Technologies, United States, A3160801 and 15140-122). The cells were cultured normally at 37°C with 5% CO_2_ and saturated humidity. Antipsychotics including olanzapine (Sigma-Aldrich, St. Louis, MO, United States, PHR1825), quetiapine (Sigma-Aldrich, Q3638), risperidone (Sigma-Aldrich, R3030), and haloperidol (Sigma-Aldrich, H1512) were obtained commercially.

### Cell Viability Analysis

To test the effects of olanzapine, quetiapine, risperidone, and haloperidol on the viability of astrocytes, 5 × 10^3^ cells/well were seeded on 96-well plates. The cells were divided into five groups, which were treated with vehicle or antipsychotic drugs at 0.01, 0.1, 1, or 10 μM. After 24-, 48-, or 72-h treatment, a cell counting kit 8 (cck8) (DOJINDO, Beijing, China, CK04) was used to determine cell viability. Antipsychotics including olanzapine, quetiapine, risperidone, and haloperidol were dissolved in dimethyl sulfoxide (DMSO) (Sigma-Aldrich, St. Louis, MO, United States, D2650) to prepare 10 mM stock solutions. They were subsequently diluted with DMEM to 0.01, 0.1, 1, or 10 μM before use. Control cells were treated with DMEM + DMSO (vehicle).

### The Effects of Antipsychotics on the Key Markers of Pyroptosis

To determine the dose-dependent effects of olanzapine, risperidone, quetiapine, and haloperidol on the protein expression of NLRP3, NLRP6, caspase-1, caspase-4, GSDMD, and NLRC4 in cultured astrocytes, cells were seeded on a six-well plate at a density of 9 × 10^5^ cells/well. The cells were treated with 0, 0.1, 1, or 10 μM olanzapine, risperidone, quetiapine, or haloperidol. After treatment for 24 h, the cell lysates were collected and stored at –80°C for western blot analysis.

### The Effects of Co-treatment of Antipsychotics With FMPH on NLRP3/Caspase-1 Signaling

To investigate whether H1 receptor activation could inhibit pyroptosis NLRP3/caspase-1 signaling induced by antipsychotics, cultured astrocytes were co-treated with an antipsychotic and a selective H1 receptor agonist, FMPH (Sigma-Aldrich, T4951). In brief, astrocytes were seeded on six-well plates at a density of 9 × 10^5^ cells/well. The cells were divided into five groups: group 1, control (Con); group 2, antipsychotic drug (10 μM); group 3, antipsychotic drug + FMPH high dose (10 μM) (antipsychotic + FMPH H); group 4, antipsychotic drug + FMPH low dose (5 μM) (antipsychotic + FMPH L); group 5: control + FMPH high dose (10 μM) (FMPH H). The FMPH doses were based on our previous study (Chen et al., 2020b). FMPH was dissolved in water to prepare a 10 mM stock solution, which was then diluted with DMEM to 10 and 5 μM. Cells were treated with FMPH for 2 h, and then were treated with the appropriate combination of FMPH and antipsychotic for 24 h. The control group was treated with DMEM + water. This treatment protocol was based on previous studies (Zhang et al., 2017; Chen et al., 2020a). Pretreatment with FMPH for 2 h allows FMPH to bind to the H1 receptor and exert physiological functions prior to olanzapine exposure. The treatment groups were determined based on previous studies (He et al., 2014) and the results of the pre-tests. Specifically, we examined the dose-dependent effects of FMPH on cell viability and NLRP3 protein expression in astrocytes without olanzapine treatment (Supplementary Figures 1, 2). Treatment with either FMPH high dose or FMPH low dose did not affect cell survival and NLRP3 expression. Therefore, we set up a control + FMPH high-dose group to investigate the effect of FMPH on NLRP3 signaling, but we did not set up a control + FMPH low-dose group. After the treatment, the cell lysates were collected and stored at –80°C.

### The Effects of Antipsychotics and FMPH Treatment on Cell Morphology

The effects of antipsychotics and FMPH treatment on cell morphological changes were investigated based on previous studies (Chen et al., 2016; Zheng et al., 2021). In brief, 1 × 10^5^ cells/well were seeded on glass coverslips placed in six-well plates. The cells were treated with vehicle, antipsychotic drug (10 μM), antipsychotic drug + FMPH high dose (10 μM), or FMPH high dose (10 μM) for 24 h. A scanning electron microscope (TESCAN, Czech, VEGA 3 LMU) was used to confirm the morphological changes of cells treated with vehicle, each antipsychotic drug, or each antipsychotic drug + FMPH high dose (Figure 10). Cells were fixed with 2.5% glutaraldehyde and rinsed with phosphate-buffered saline three times. Then, the cells were dehydrated through a graded ethanol series (50, 70, 80, 90, and 100%) and dried by an automated dryer (Leica, German, EM CPD300). The dried specimens were coated with gold-palladium by using an ion sputter coater (QUORUM, United Kingdom, Q150RS). The images were taken by using the scanning electron microscope.

### Western Blot Analysis

Western blot analysis was performed according to our previous study (He et al., 2020). Radioimmunoprecipitation assay (RIPA) cell lysis buffer was used to homogenize the cells. A BCA protein quantification kit (Absin, Wuhan, China, abs9232) was used to determine the protein concentration of each sample. The protein samples were loaded onto 8% gels (Beyotime, Wuhan, China, P0452S). The electrophoresis conditions were 80 V for 30 min, and then 120 V for 60 min. Then, the proteins were transferred to polyvinylidene difluoride (PVDF) membranes (Millipore, Ireland, IPVH00010). The PVDF membranes were blocked with 5% non-fat milk in tris-buffered saline with tween-20 (TBST) for 1.5 h and then incubated with the primary antibodies at 4°C overnight. The primary antibodies used were: anti-GSDMD (Affinity, AF4012), anti-caspase-4 (Affinity, DF7609), anti-NLRC4 (ABclonal, Wuhan, China, A7382), anti-NLRP6 (ABclonal, A15628), anti-NLRP3 (Cell Signaling Technology, Danvers, MA, United States, D4D8T), anti-caspase-1 (Huabio, Boston, United States, ET1608-69), anti-IL-1β (Affinity, Jiangsu, China, AF5103) and anti-β-actin (Affinity, AF7018). The membranes were washed in TBST and then incubated with goat anti-mouse (1:8,000, Affinity, S0002) or goat anti-rabbit (Proteintech, Wuhan, China, SA00001-2) secondary antibody for 1.5 h at room temperature. The bands were detected with a super-sensitive ECL chemiluminescence kit (Biosharp, Wuhan, China, BL520B).

### Statistics

The data were analyzed with SPSS 22.0 (IBM Corp., Armonk, NY, United States). One-way analysis of variance (ANOVA) followed by Dunnett’s t test was used to analyze the difference in cell viability and the protein expression of caspase-1, caspase-4, NLRC4, NLRP3, NLRP6, GSDMD, and IL-1β. All data are presented as the mean ± standard error of the mean. Statistical significance was defined as *p* < 0.05.

## Results

### Antipsychotics Differentially Affected the Viability of Astrocytes

**FIGURE 1:**
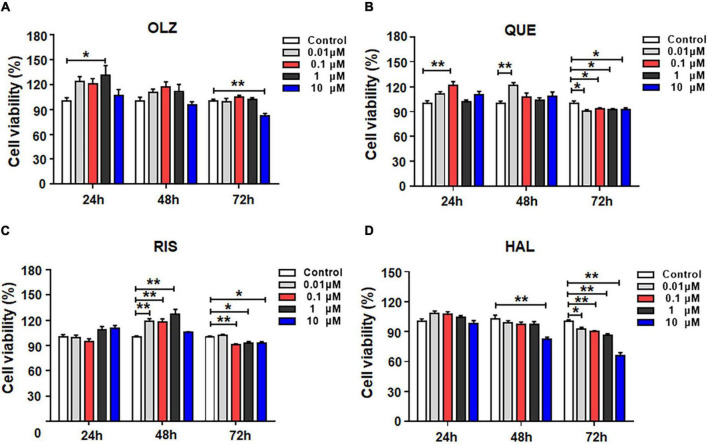
The effects of 0.01, 0.1, 1, and 10 μM olanzapine **(A)**, quetiapine **(B)**, risperidone **(C)**, and haloperidol **(D)** treatment for 24, 48, and 72 h on the viability of cultured astrocytes. The data are presented as the mean ± standard error of the mean. *p < 0.05, **p < 0.01 vs. control (one-way analysis of variance and *post hoc* Dunnett’s multiple comparison test; *n* = 6–12 cultures/group). Con, control; OLZ, olanzapine; QUE, quetiapine; RIS, risperidone; HAL, haloperidol.

To evaluate the effects of different antipsychotics—olanzapine, quetiapine, risperidone and haloperidol—on the viability of astrocytes, the cells were incubated with 0, 0.01, 0.1, 1, or 10 μM of each antipsychotic or vehicle for 24, 48, or 72 h. As shown in Figure 1A, after 24-h treatment, olanzapine 1 μM but not 0.01, 0.1, or 10 μM significantly increased cell viability (by 31.5 ± 10.5%, *p* = 0.014). After 48-h treatment, olanzapine 0.1 and 1 μM slightly increased cell viability (by 17.4 ± 8.0%, *p* = 0.1, and 11.9 ± 7.8%, *p* = 0.37, respectively). Olanzapine 0.01 or 10 μM did not affect cell viability. After 72-h treatment, olanzapine 10 μM significantly reduced cell viability (by 17.6 ± 3.7%, *p* = 0.001).

The effects of quetiapine on cell viability are shown in Figure 1B. Similarly to olanzapine, 24-h treatment with quetiapine 0.1 μM increased cell viability (by 21.7 ± 5.4%, *p* = 0.001). As treatment was prolonged, quetiapine 0.01 μM significantly increased cell viability (by 21.3 ± 5.2%, *p* = 0.001). However, quetiapine 0.1–10 μM did not alter cell viability. After 72-h treatment, quetiapine 0.01–10 μM treatment slightly but significantly decreased cell viability (0.01 μM, by 10.4 ± 7.9%, *p* = 0.01; 0.1 μM, by 7.1 ± 1.7%, *p* = 0.035; 1 μM, by 8.1 ± 1.6%, *p* = 0.035; 10 μM, by 8.2 ± 2.5%, *p* = 0.049, respectively).

The effects of risperidone on the viability of astrocytes are shown in Figure 1C. After 24-h treatment, risperidone 0.01–10 μM did not affect cell viability. After 48 h, risperidone 0.01–1 μM evidentially increased cell viability (0.01 μM, by 19.1 ± 4.9%, *p* = 0.000; 0.1 μM, by 17.7 ± 5.0%, *p* = 0.001; 1 μM, by 27.4 ± 5.0%, *p* = 0.001). After 72 h, risperidone 0.1–10 μM slightly decreased cell viability (0.1 μM, by 9.5 ± 2.7%, *p* = 0.004; 1 μM, by 7.1 ± 2.6%, *p* = 0.031; 10 μM, by 7.5 ± 2.6%, *p* = 0.019).

The effects of haloperidol on the viability of astrocytes are shown in Figure 1D. In contrast to olanzapine, quetiapine, risperidone, and haloperidol treatment for 24 did not alter cell viability. After 48 h, haloperidol 10 μM decreased cell viability (by 20.3 ± 4.7%, *p* = 0.004). Moreover, after 72-h treatment, haloperidol 0.01–10 μM reduced cell viability (0.01 μM, by 7.2 ± 3.5%, *p* = 0.015; 0.1 μM, by 8.8 ± 3.6%, *p* = 0.002; 1 μM, by 14.0 ± 3.5%, *p* = 0.002; 10 μM, by 34.0 ± 3.5%, *p* = 0.001). Overall, during short-term treatment, olanzapine, quetiapine, and risperidone, but not haloperidol, slightly increased astrocyte viability. However, as treatment was prolonged, all four antipsychotics induce cell death. Haloperidol had the largest effect of inducing astrocyte death.

### Olanzapine Activated NLRP3, NLRP6, and NLRC4 Pyroptotic Signaling in a Dose-Dependent Manner in Cultured Astrocytes

**FIGURE 2:**
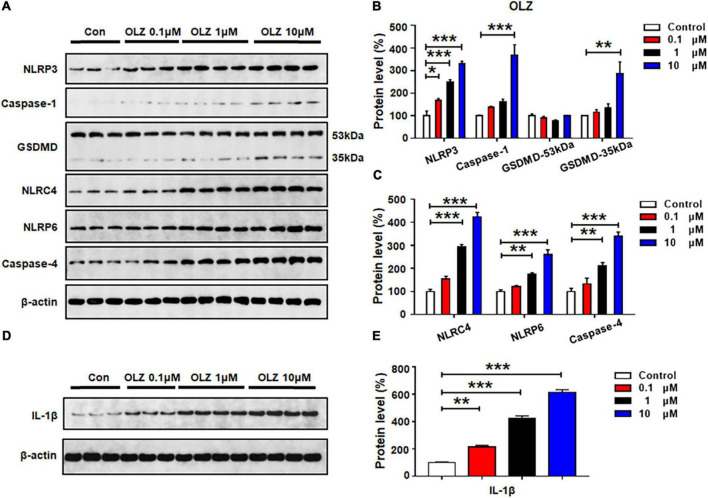
Olanzapine treatment activated NLRP3/caspase-1 signaling, and increased NLRC4, NLRP6, and IL-1β protein expression in cultured astrocytes. Cultured cells were treated with 0.1, 1, or 10 μM olanzapine for 24 h. **(A–E)** Western blot was used to evaluate the expression of NLRP3, caspase-1 (20 kDa), GSDMD (53 and 35 kDa), NLRC4, NLRP6, and IL-1β. The data are presented as the mean ± standard error of the mean. *p < 0.05, **p < 0.01, ***p < 0.001 vs. control (one-way analysis of variance and *post hoc* Dunnett’s multiple comparison test; *n* = 3–4 cultures/western blot group). Con, control; OLZ, olanzapine.

Astrocytes were treated with olanzapine 0, 0.1, 1, or 10 μM for 24 h, and western blot analysis was used to examine the protein expression of NLRP3, caspase-1, GSDMD, NLRC4, NLRP6, caspase-4, and IL-1β. As shown in Figures 2A–C, olanzapine 0.1 μM significantly increased NLRP3 (by 67.7 ± 19.6%, *p* = 0.015), but it did not affect the expression of caspase-1, GSDMD, NLRC4, NLRP6, or caspase-4. Olanzapine 1 μM upregulated NLRP3 (by 150.9 ± 9.0%, *p* = 0.000), NLRC4 (by 194.5 ± 8.8%, *p* = 0.000), NLRP6 (by 74.5 ± 6.7%, *p* = 0.007), and caspase-4 (by 111.2 ± 14.4%, *p* = 0.005). Olanzapine 1 μM did not affect the expression of caspase-1 or GSDMD. Olanzapine 10 μM markedly increased NLRP3 (by 230.2 ± 11.8%, *p* = 0.000), caspase-1 (by 267.0 ± 47.2%, *p* = 0.001), and GSDMD 35 kDa (by 186.2 ± 52.4%, *p* = 0.006), but not GSDMD 53 kDa. Olanzapine 10 μM also increased NLRC4 (by 324.0 ± 19.9%, *p* = 0.000), NLRP6 (by 160.4 ± 20.6%, *p* = 0.000), and caspase-4 (by 239.1 ± 19.3%, *p* = 0.000). Moreover, olanzapine 0.1–10 μM markedly upregulated IL-1β (0.1 μM, by 116.0 ± 11.9%, *p* = 0.003; 1 μM, by 321.4 ± 20.0%, *p* = 0.000; 10 μM, 514.1 ± 19.2%, *p* = 0.000, respectively) (Figures 2D,E). Overall, olanzapine dose-dependently increased NLRP3, NLRP6, NLRC4, caspase-1, caspase-4, and GSDMD 35 kDa, changes that could activate the relevant signaling and be associated with olanzapine-induced inflammation.

### Quetiapine Dose-Dependently Activated NLRP3, NLRP6, and NLRC4 Pyroptotic Signaling

**FIGURE 3:**
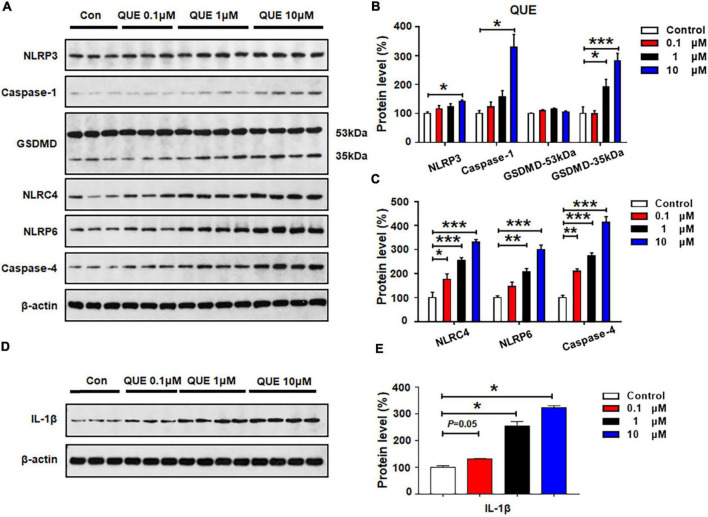
Quetiapine treatment activated NLRP3/caspase-1 signaling and upregulated NLRC4, NLRP6, and IL-1β protein expression in cultured astrocytes. Cells were treated with 0.1, 1, or 10 μM quetiapine for 24 h. **(A–E)** Western blot was used to examine the expression of NLRP3, caspase-1 (20 kDa), GSDMD (53 and 35 kDa), NLRC4, NLRP6, and IL-1β. The data are presented as the mean ± standard error of the mean. *p < 0.05, **p < 0.01, ***p < 0.001 vs. control (one-way analysis of variance and *post hoc* Dunnett’s multiple comparison test; *n* = 3–4 cultures/western blot group). Con, control; QUE, quetiapine.

As shown in Figures 3A–C, quetiapine 0.1 μM did not affect the protein expression of NLRP3, caspase-1, GSDMD, or NLRP6, but this treatment significantly increased the expression of NLRC4 (by 77.4 ± 21.9%, *p* = 0.029) and caspase-4 (by 109.5 ± 10.8%, *p* = 0.005). These data suggest that a relatively low dose of quetiapine could activate NLRC4 and caspase-4 signaling. When the concentration of quetiapine increased to 1 μM, besides increasing the expression of NLRC4 (by 155.1 ± 11.9%, *p* = 0.000) and caspase-4 (by 173.9 ± 12.9%, *p* = 0.000), quetiapine also increased the expression of GSDMD 35 kDa (by 93.2 ± 25.2%, *p* = 0.04) and NLRP6 (by 107.4 ± 14.5%, *p* = 0.002). After 24-h treatment with quetiapine 10 μM, there was significant upregulation of NLRP3, caspase-1, GSDMD 35 kDa, NLRC4, NLRP6, and caspase-4 (NLRP3, by 42.3 ± 3.0%, *p* = 0.02; caspase-1, by 228.3 ± 45.0%, *p* = 0.034; GSDMD 35 kDa, by 82.9 ± 24.7%, *p* = 0.000; NLRC4, by 230.3 ± 12.1%, *p* = 0.000; NLRP6, by 201.5 ± 17.6%, *p* = 0.000; caspase-4, by 313.7 ± 23.9%, *p* = 0.000). Furthermore, quetiapine 0.1–10 μM markedly upregulated IL-1β expression (0.1 μM, by 30.7 ± 1.5%, *p* = 0.05; 1 μM, by 154.6 ± 16.7%, *p* = 0.034; 10 μM, 222.2 ± 8.5%, *p* = 0.034, respectively) (Figures 3D,E). Taken together, a relatively low dose of quetiapine upregulates NLRC4 and caspase-4 but not NLRP3. As the concentration increases, both NLRP3 and NLRP6 signaling are also activated.

### Risperidone Activated NLRP3, NLRP6, and NLRC4 Pyroptotic Signaling

**FIGURE 4:**
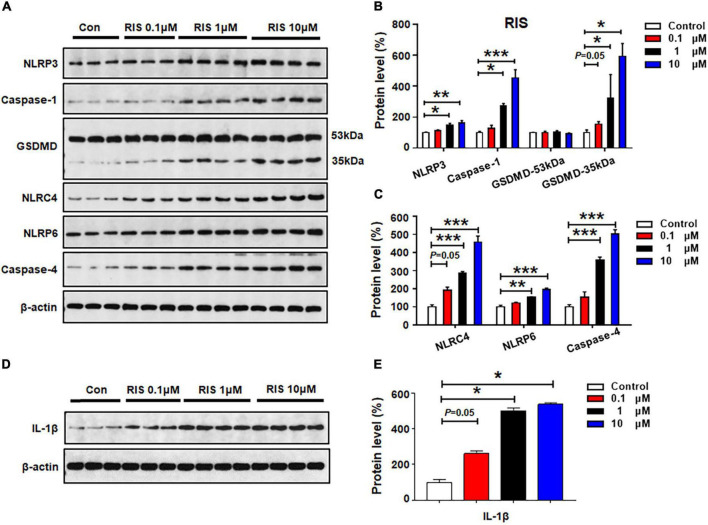
Risperidone treatment activated NLRP3/caspase-1 signaling and increased NLRC4, NLRP6, and IL-1β protein expression in cultured astrocytes. Cells were treated with 0.1, 1, or 10 μM risperidone for 24 h. **(A–E)** Western blot was used to examine the expression of NLRP3, caspase-1 (20 kDa), GSDMD (53 and 35 kDa), NLRC4, NLRP6, and IL-1β. The data are presented as the mean ± standard error of the mean. *p < 0.05, **p < 0.01, ***p < 0.001 vs. control (one-way analysis of variance and *post hoc* Dunnett’s multiple comparison test; *n* = 3–4 cultures/western blot group). Con, control; RIS, risperidone.

The effects of 24-h risperidone treatment on NLRP3, capase-1, GSDMD, NLRC4, NLRP6, and caspase-4 protein expression are shown in Figures 4A–C. Low-dose risperidone (0.1 μM) upregulated NLRC4 (by 92.1 ± 16.9%, *p* = 0.05) and GSDMD 35 kDa (by 54.1 ± 9.9%, *p* = 0.05), but not NLRP3, caspase-1, NLRP6, or caspase-4 (*p* > 0.05). Treatment with risperidone 1 μM elevated the expression of NLRP3 (by 50.3 ± 9.8%, *p* = 0.024), caspase-1 (by 174.4 ± 13.2%, *p* = 0.011), NLRC4 (by 185.7 ± 9.5%, *p* = 0.000), NLRP6 (by 54.0 ± 2.1%, *p* = 0.001), caspase-4 (by 257.0 ± 18.3%, *p* = 0.000), and GSDMD 35 kDa (by 222.7 ± 76.2%, *p* = 0.034), but not GSDMD 53 kDa. Risperidone at a relatively higher dose (10 μM) notably increased NLRP3 (by 62.1 ± 15.4%, *p* = 0.007), caspase-1 (by 352.7 ± 53.8%, *p* = 0.000), GSDMD 35 kDa (by 493.1 ± 41.3%, *p* = 0.034), NLRC4 (by 357.4 ± 66.5%, *p* = 0.000), NLRP6 (by 95.3 ± 9.6%, *p* = 0.000), and caspase-4 (by 401.7 ± 24.5%, *p* = 0.000). Risperidone 0.1–10 μM upregulated IL-1β (0.1 μM, by 161.1 ± 16.2%, *p* = 0.05; 1 μM, by 401.7 ± 16.6%, *p* = 0.034; 10 μM, 438.4 ± 9.1%, *p* = 0.034, respectively) (Figures 4D,E).

### Haloperidol Activated NLRP3, NLRP6, and NLRC4 Pyroptotic Signaling

**FIGURE 5:**
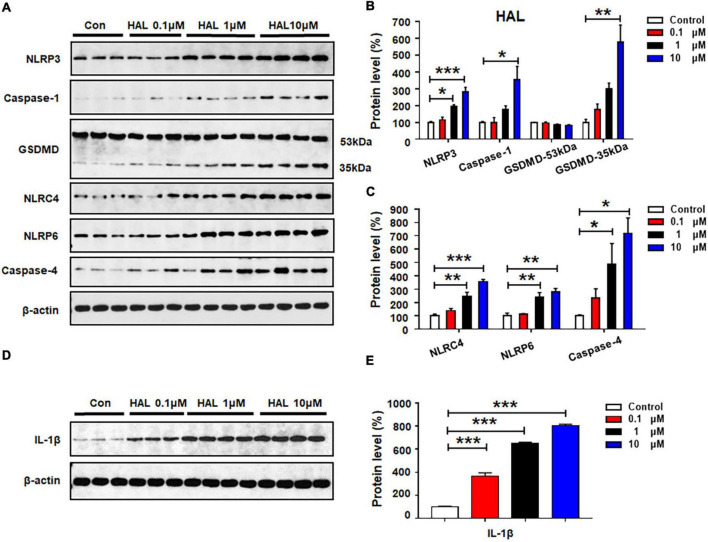
Haloperidol treatment activated NLRP3/caspase-1 signaling and increased NLRC4, NLRP6, and IL-1β protein expression in cultured astrocytes. Cells were treated with 0.1, 1, or 10 μM haloperidol for 24 h. **(A–E)** Western blot was used to examine the expression of NLRP3, caspase-1 (20 kDa), GSDMD (53 and 35 kDa), NLRC4, NLRP6, and IL-1β. The data are presented as the mean ± standard error of the mean. *p < 0.05, **p < 0.01, ***p < 0.001 vs. control (one-way analysis of variance and *post hoc* Dunnett’s multiple comparison test; *n* = 3–4 cultures/western blot group). Con, control; HAL, haloperidol.

Haloperidol 0.1 μM did not affect the expression of the investigated proteins (all *p* > 0.05) (Figures 5A–C). At 1 μM, there was significant upregulation of NLRP3 (by 93.3 ± 11.9%, *p* = 0.014), but not caspase-1 or GSDMD. Haloperidol 1 μM increased the expression of NLRC4 (by 148.8 ± 28.5%, *p* = 0.001), NLRP6 (by 142.8 ± 31.9%, *p* = 0.008), and caspase-4 (by 387.3 ± 155.3%, *p* = 0.012). Moreover, haloperidol 10 μM increased NLRP3 (by 182.3 ± 26.5%, *p* = 0.000), caspase-1 (by 254.3 ± 78.4%, *p* = 0.012), GSDMD 35 kDa (by 475.3 ± 102.8%, *p* = 0.001), NLRC4 (by 257.1 ± 15.7%, *p* = 0.000), NLRP6 (by 179.2 ± 27.0%, *p* = 0.002), and caspase-4 (by 617.2 ± 116.4%, *p* = 0.011). Haloperidol 0.1–10 μM markedly upregulated IL-1β (0.1 μM, by 265.7 ± 29.2%, *p* = 0.000; 1 μM, by 549.3 ± 10.8%, *p* = 0.000; 10 μM, 704.1 ± 13.3%, *p* = 0.000, respectively) (Figures 5D,E). Based on the results presented in sections 3.2–3.5, each of the tested antipsychotics—olanzapine, quetiapine, risperidone, and haloperidol—significantly increased the expression of proteins involved in pyroptotic signaling. These effects may be largely responsible for the astrocyte death induced by 72-h antipsychotic treatment.

### Antipsychotic-Induced Activation of NLRP3/Caspase-1 Signaling Was Inhibited by Co-treatment With a Histamine H1 Receptor Agonist

**FIGURE 6:**
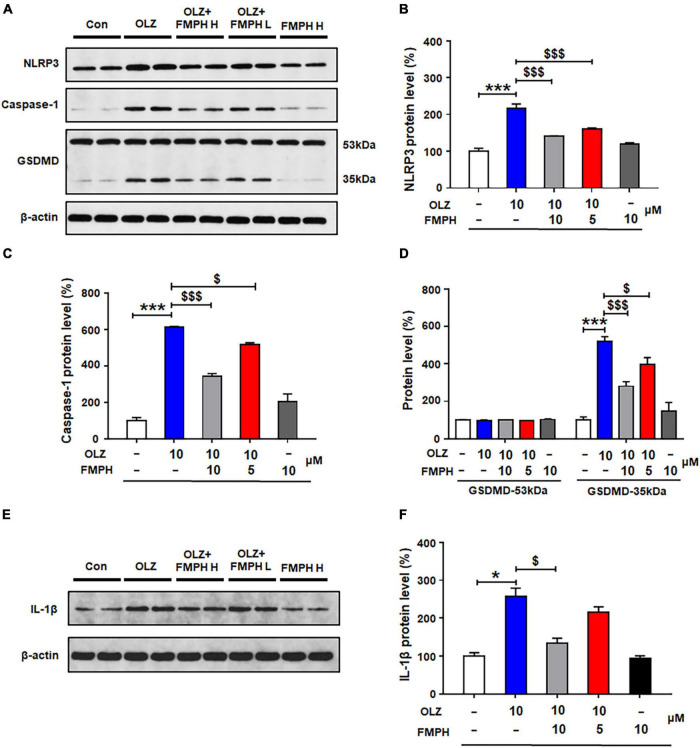
2-(3-Trifluoromethylphenyl) histamine (FMPH) co-treatment inhibited olanzapine-induced NLRP3/caspase-1 signaling activation and reduced IL-1β protein expression in cultured astrocytes. **(A–F)** Cultured cells were treated with vehicle [dimethyl sulfoxide (DMSO) + water], olanzapine 10 μM, olanzapine 10 μM + FMPH 10 μM, olanzapine 10 μM + FMPH 5 μM, or FMPH 10 μM for 24 h. Western blot was used to examine the expression of NLRP3, caspase-1 (20 kDa), GSDMD (53 and 35 kDa), and IL-1β. The data are presented as the mean ± standard error of the mean. *p < 0.05, ***p < 0.001 vs. Con; $*p* < 0.05, $$$*p* < 0.001 vs. OLZ (one-way analysis of variance and *post hoc* Dunnett’s multiple comparison test; *n* = 4 cultures/western blot group). Con, control; OLZ, olanzapine; OLZ + FMPH H, olanzapine + FMPH high dose; OLZ + FMPH L, olanzapine + FMPH low dose; FMPH H, FMPH high dose.

To determine whether the effects of antipsychotics on astrocytic NLRP3/caspase-1 signaling are related to the histamine H1 receptor, we examined whether the selective H1 receptor agonist FMPH could suppress antipsychotic-induced increased protein expression of NLRP3, caspase-1, GSDMD and IL-1β. Compared with vehicle, 24-h olanzapine treatment significantly upregulated NLRP3 (by 116.2 ± 12.4%, *p* = 0.000), caspase-1 (by 512.5 ± 3.6%, *p* = 0.000), GSDMD 35 kDa (by 419.9 ± 24.8%, *p* = 0.000), and IL-1β (by 157.1 ± 21.9%, *p* = 0.021) (Figures 6A–F). Co-treatment with FMPH high or low dose inhibited olanzapine-induced expression of NLRP3 (high dose, by 75.7 ± 1.6%, *p* = 0.000; low dose, by 55.0 ± 2.1%, *p* = 0.000), caspase-1 (high dose, by 269.8 ± 14.6%, *p* = 0.000; low dose, by 98.5 ± 12.6%, *p* = 0.026), GSDMD 35 kDa (high dose, by 239.3 ± 24.6%, *p* = 0.000; low dose, by 123.7 ± 37.8%, *p* = 0.026), and IL-1β (high dose, by122.9 ± 13.0%, *p* = 0.021; low dose, by 41.9 ± 14.5%, *p* = 0.083).

**FIGURE 7:**
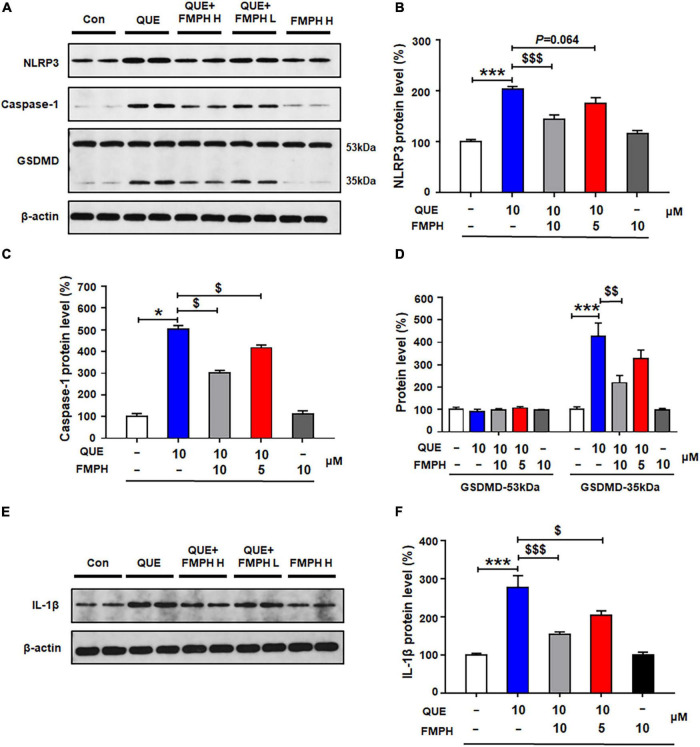
2-(3-Trifluoromethylphenyl) histamine (FMPH) co-treatment reversed quetiapine induced activation of NLRP3/caspase-1 signaling and inhibited IL-1β protein expression in cultured astrocytes. **(A–F)** Cultured cells were treated with vehicle [dimethyl sulfoxide (DMSO) + water], quetiapine 10 μM, quetiapine10 μM + FMPH 10 μM, quetiapine 10 μM + FMPH 5 μM, or FMPH 10 μM for 24 h. Western blot was used to examine the expression of NLRP3, caspase-1 (20 kDa), GSDMD (53 and 35 kDa), and IL-1β. The data are presented as the mean ± standard error of the mean. *p < 0.05, ***p < 0.001 vs. Con; $*p* < 0.05, $$*p* < 0.01, $$$*p* < 0.001 vs. QUE (one-way analysis of variance and *post hoc* Dunnett’s multiple comparison test; *n* = 4 cultures/western blot group). Con, control; QUE, quetiapine; QUE + FMPH H, quetiapine + FMPH high dose; QUE + FMPH L, quetiapine + FMPH low dose; FMPH H, FMPH high dose.

Compared with vehicle, 24-h quetiapine treatment significantly upregulated NLRP3 (by 103.6 ± 5.5%, *p* = 0.000), caspase-1 (by 402.4 ± 17.4%, *p* = 0.021), GSDMD 35 kDa (by 325.5 ± 60.7%, *p* = 0.000), and IL-1β (by 177.2 ± 30.8%, *p* = 0.000) (Figures 7A–F). Co-treatment with either FMPH high or low dose counteracted the quetiapine-induced increased expression of NLRP3 (high dose, by 58.7 ± 8.2%, *p* = 0.000; low dose, by 27.6 ± 11.0%, *p* = 0.064), caspase-1 (high dose, by 200.8 ± 11.1%, *p* = 0.021; low dose, by 85.3 ± 13.4%, *p* = 0.021), GSDMD 35 kDa (high dose, by 205.9 ± 33.3%, *p* = 0.004; low dose, by 98.8 ± 39.2%, *p* = 0.207), and IL-1β (high dose, by 123.6 ± 6.5%, *p* = 0.000; low dose, by 70.1 ± 11.3%, *p* = 0.015).

**FIGURE 8:**
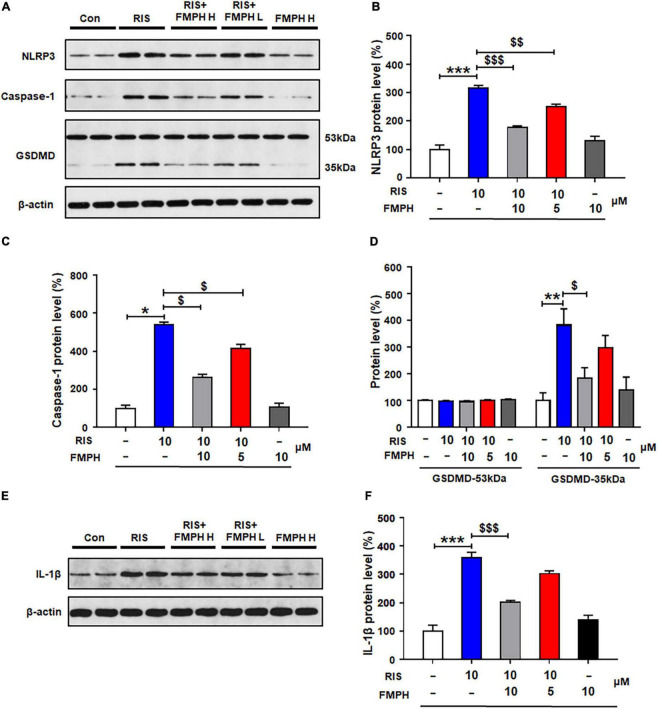
2-(3-Trifluoromethylphenyl) histamine (FMPH) inhibited risperidone-induced activation of NLRP3/caspase-1 signaling and inhibited IL-1β protein expression in cultured astrocytes. **(A–F)** Cultured cells were treated with vehicle [dimethyl sulfoxide (DMSO) + water], risperidone 10 μM, risperidone 10 μM + FMPH 10 μM, risperidone 10 μM + FMPH 5 μM, or FMPH 10 μM for 24 h. Western blot was used to investigate the expression of NLRP3, caspase-1 (20 kDa), GSDMD (53 and 35 kDa), and IL-1β. The data are presented as mean ± standard error of the mean. *p < 0.05, **p < 0.01, ***p < 0.001 vs. Con; $*p* < 0.05, $$*p* < 0.01, $$$*p* < 0.001 vs. RIS (one-way analysis of variance and *post hoc* Dunnett’s multiple comparison test; *n* = 4 cultures/western blot group). Con, control; RIS, risperidone; RIS + FMPH H, risperidone + FMPH high dose; risperidone + FMPH L, risperidone + FMPH low dose; FMPH H, FMPH high dose.

Compared with vehicle, 24-h risperidone treatment significantly upregulated NLRP3 (by 215.1 ± 10.6%, *p* = 0.000), caspase-1 (by 402.4 ± 17.4%, *p* = 0.021), GSDMD 35 kDa (by 281.6 ± 61.7%, *p* = 0.002), and IL-1β (by 259.3 ± 18.4%, *p* = 0.000) (Figures 8A–F). Co-treatment with either FMPH high or low dose ameliorated the risperidone-induced increased expression of NLRP3 (high dose, by 136.2 ± 4.6%, *p* = 0.000; low dose, by 63.3 ± 8.1%, *p* = 0.006), caspase-1 (high dose, by 200.8 ± 11.1%, *p* = 0.021; low dose, by 85.3 ± 13.4%, *p* = 0.021), GSDMD 35 kDa (high dose, by 198.0 ± 38.7%, *p* = 0.028; low dose, by 85.5 ± 47.2%, *p* = 0.513), and IL-1β (high dose, by 157.2 ± 6.0%, *p* = 0.000; low dose, by 57.1 ± 10.3%, *p* = 0.000).

**FIGURE 9:**
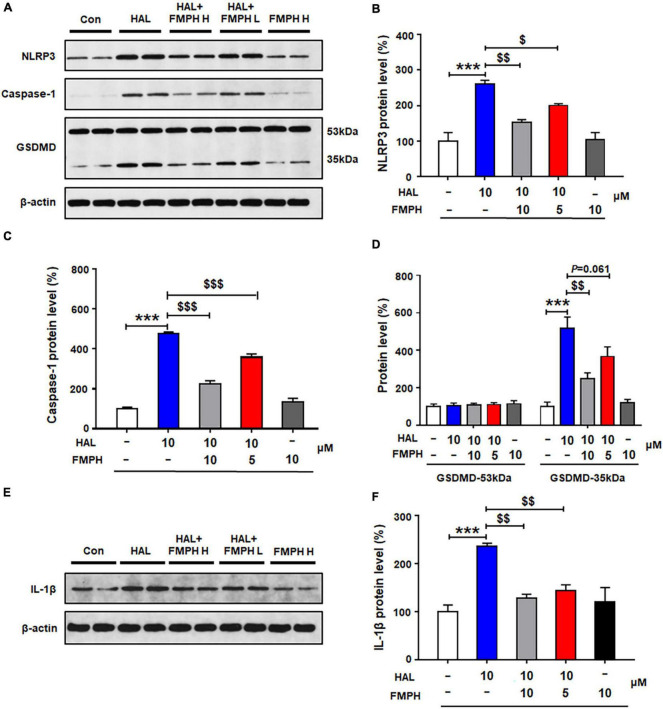
Co-treatment with 2-(3-trifluoromethylphenyl) histamine (FMPH) suppressed haloperidol-induced activation of NLRP3/caspase-1 and inhibited IL-1β protein expression in cultured astrocytes. **(A–F)** Cultured cells were treated with vehicle [dimethyl sulfoxide (DMSO) + water], haloperidol 10 μM, haloperidol 10 μM + FMPH 10 μM, haloperidol 10 μM + FMPH 5 μM, or FMPH 10 μM for 24 h. Western blot was used to investigate the expression of NLRP3, caspase-1 (20 kDa), GSDMD (53 and 35 kDa), and IL-1β. The data are presented as mean ± standard error of the mean. ***p < 0.001 vs. Con; $*p* < 0.05, $$*p* < 0.01, $$$*p* < 0.001 vs. HAL (one-way analysis of variance and *post hoc* Dunnett’s multiple comparison test; *n* = 4 cultures/western blot group). Con, control; HAL, haloperidol; HAL + FMPH H, haloperidol + FMPH high dose; haloperidol + FMPH L, haloperidol + FMPH low dose; FMPH H, FMPH high dose.

Similarly to the other three antipsychotics, compared with vehicle, 24-h haloperidol treatment significantly upregulated NLRP3 (by 160.1 ± 11.5%, *p* = 0.000), caspase-1 (by 376.8 ± 8.5%, *p* = 0.000), GSDMD 35 kDa (by 417.1 ± 60.4%, *p* = 0.000) and IL-1β (by 135.8 ± 6.8%, *p* = 0.000) (Figures 9A–F). Co-treatment with either FMPH high or low dose counteracted the haloperidol-induced increased expression of NLRP3 (high dose, by 106.8 ± 7.1%, *p* = 0.001; low dose, by 59.6 ± 4.7%, *p* = 0.046), caspase-1 (high dose, by 252.6 ± 16.8%, *p* = 0.000; low dose, by 118.3 ± 16.4%, *p* = 0.000), GSDMD 35 kDa (high dose, by 260.0 ± 31.5%, *p* = 0.001; low dose, by 150.9 ± 52.4%, *p* = 0.061) and IL-1β (high dose, by 107.7 ± 8.3%, *p* = 0.001; low dose, by 92.3 ± 12.3%, *p* = 0.004).

### Antipsychotic-Induced Pore Formation in the Cell Membrane Was Inhibited by FMPH Co-treatment

**FIGURE 10:**
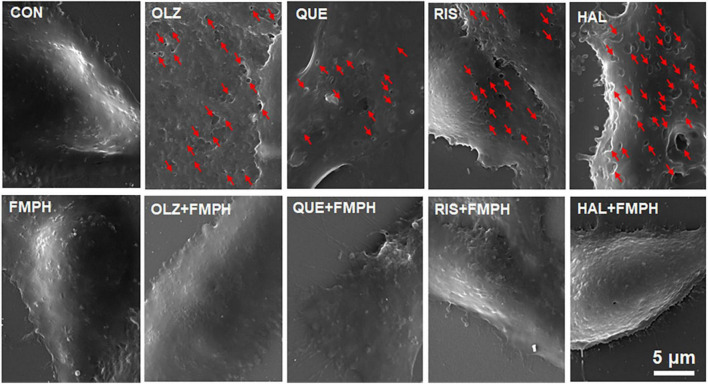
2-(3-Trifluoromethylphenyl) histamine (FMPH) inhibited antipsychotic-induced pore formation in the cell membrane. SEM Images of astrocytes treated with vehicle, antipsychotic drug (olanzapine/quetiapine/risperidone/haloperidol), or antipsychotic drug + FMPH 10 μM were taken by a Tescan Vega-3 LMU emission scanning electron microscope operating at 20 kV. Red arrow indicates pores in the cell membrane. Con, control; OLZ, olanzapine; QUE, quetiapine; RIS, risperidone; HAL, haloperidol; FMPH,2-(3-Trifluoromethylphenyl) histamine.

Astrocyte pyroptosis involves cell swelling, pore formation in the cell membrane and increased membrane permeabilization (Chen et al., 2016; Zheng et al., 2021). The present study found that FMPH high dose co-treatment significantly inhibited olanzapine-, quetiapine-, risperidone-, or haloperidol-induced increased expression of NLRP3, caspase-1, GSDMD and IL-1β. Therefore, the effects of antipsychotics and FMPH high dose treatment on astrocyte cell morphological changes were examined. As shown in Figure 10, compared with vehicle, FMPH treatment did not affect the cell morphology. Compared with vehicle, certain cells treated with 10 μM of olanzapine, quetiapine, risperidone, or haloperidol had numerous pits or pores of different sizes across the cell membranes. FMPH 10 μM significantly inhibited the pore formation in the membrane induced by treatment with olanzapine, quetiapine, risperidone, or haloperidol. These findings showed that astrocytes treated with olanzapine, quetiapine, risperidone, or haloperidol exhibited pyroptosis-like morphological changes, and these effects could be partly inhibited by FMPH treatment.

## Discussion

Emerging data have shown that astrocytes are the primary homeostatic cells in the CNS and that they affect neuronal development and function, metabolism, neuronal injury, and inflammation. In the present study, we have found that second-generation antipsychotics, including olanzapine, quetiapine, and risperidone, increased the viability of astrocytes during short-term treatment (24–48 h), but decreased their viability during long-term treatment (72 h). Consistently with this study, we had previously found that short-term (24 h) olanzapine treatment increased the protein expression of astrocyte markers, including calcium binding protein B (S100B) and GFAP, in cultured astrocytes, while long-term (72 h) olanzapine treatment led to astrocyte death (He et al., 2020). These findings are consistent with animal studies, which have shown that short-term olanzapine treatment activated astrocytes in the rat brain (He et al., 2020) while chronic exposure to olanzapine reduced the number of astrocytes in the parietal cortex of monkeys (Konopaske et al., 2008). Furthermore, previous studies have reported that risperidone increased S100B secretion from C6 glioma cells after 1-h treatment (de Souza et al., 2013) and increased S100B expression of primary cultured astrocytes after 24-h treatment (Nardin et al., 2011). In mice, chronic quetiapine treatment mitigated astrocyte activation induced by cuprizone (Shao et al., 2015). In contrast to the second-generation antipsychotics, the first-generation antipsychotic drug haloperidol did not increase the viability of astrocytes during short-term (24 h) treatment, but decreased the viability as treatment was prolonged to 48 and 72 h. Consistently with this study, researchers have reported that in primary astrocytes, haloperidol (10 μM) did not change S100B secretion after 24-h treatment (Nardin et al., 2011). Chronic haloperidol treatment induced astrocyte loss in the monkey parietal cortex (Konopaske et al., 2008). Our findings suggest that chronic treatment with the antipsychotics olanzapine, quetiapine, risperidone, or haloperidol induces astrocyte death, and these effects may contribute at least partly to antipsychotic-induced brain volume loss and cortical thinning.

Currently, how antipsychotics induce astrocyte death remains elusive. Numerous studies have reported that antipsychotic treatment, in particular olanzapine and risperidone, is associated with increased expression of proinflammatory cytokines such as IL-1β and IL-6, suggesting the possible involvement of proinflammatory programmed cell death (Zhang et al., 2014; Cruz Jung et al., 2016; He et al., 2019; May et al., 2020). As has been reported, activation of NLRP3/caspase-1 and NLRC4 signaling facilitates IL-1β and IL-6 secretion and induces pyroptosis in astrocytes (Liu and Chan, 2014; Mao et al., 2018; Voet et al., 2019). An increase in IL-1β also promotes astrocyte death (van Kralingen et al., 2013). In the present study, treatment with olanzapine, quetiapine, risperidone, or haloperidol significantly increased the expression of NLRP3, NLRP6, NLRC4, caspase-1, caspase-4, GSDMD, and IL-1β in cultured astrocytes. Therefore, NLRP/caspase-1 and NLRC4 signaling in astrocytes may be partly responsible for the increased IL-1β expression and astrocyte death induced by antipsychotics. Moreover, each of the tested antipsychotics increased the protein expression of GSDMD 35 kDa but not GSDMD 53 kDa, which represent the cleaved and full-length forms of GSDMD, respectively. Activated GSDMD (35 kDa) could accumulate inside the cell membrane and facilitate the formation of membrane pores, inducing cell blebbing and pyroptosis. In our work, cell imaging revealed that antipsychotic treatment induced pore formation in the cell membrane. These findings suggest that antipsychotics may induce astrocyte pyroptosis, and this effect may be largely related to NLRP/caspase/GSDMD signaling. However, the exact mechanism by which antipsychotics induce pore formation is unknown. A recent study in astrocytes reported that activated GSDMD increased osmotic pressure (OP) and intracellular calcium, and these effects contributed to astrocyte swelling and membrane blebbing (Zheng et al., 2021). Additional studies that investigate the effects of antipsychotics on OP and calcium flux and whether these effects are related to GSDMD are warranted.

Antipsychotics could bind directly to a variety of neurotransmitter receptors to regulate physiological functions. Elucidating the receptors involved in antipsychotic-induced activation of pyroptotic signaling has important implications for developing strategies to inhibit antipsychotic-induced astrocyte death. The H1 receptor is expressed on astrocytes (Fukui et al., 1991; Karpati et al., 2019), and olanzapine, quetiapine, and risperidone have affinity for the H1 receptor. In this study, the H1 receptor agonist FMPH markedly reduced olanzapine-, quetiapine-, and risperidone-induced increased expression of NLRP3, caspase-1, GSDMD and IL-1β. FMPH also inhibited olanzapine-, quetiapine-, and risperidone-induced cell membrane pore formation. Hence, activation of NLRP3/caspase-1 signaling and cell pyroptosis induced by second-generation antipsychotics may be partly related to their antagonistic effects on the H1 receptor. However, the mechanism by which the H1 receptor regulates NLRP3/caspase-1 signaling is unclear. A recent study reported that NLRP3 signaling and pyroptosis was inhibited by rapamycin (which binds to mammalian target of rapamycin [mTOR]) in macrophages (Yang et al., 2021). Previous studies have also suggested that the NLRP3 inflammasome is activated by potassium efflux and calcium influx (Li et al., 2021a). Therefore, future studies should investigate the role of the H1 receptor in regulating mTOR and calcium influx. Furthermore, it has been reported that activation of the hypothalamic H1 receptor, such as by using betahistine (an H1 receptor agonist/H3 receptor antagonist), reduced olanzapine-induced food intake and weight gain in rodents and patients (Deng et al., 2012; Poyurovsky et al., 2013; Barak et al., 2016; Smith et al., 2018). Besides counteracting obesity, perhaps activating the H1 receptor by using betahistine, or another H1 receptor agonist, could also inhibit NLRP3/caspase-1 signaling and thus pyroptosis and brain volume loss induced by chronic antipsychotic treatment. It is worth performing preclinical studies in rodents to investigate whether central activation of the H1 receptor using betahistine or FMPH could reverse antipsychotic-induced central inflammation and astrocyte loss.

It should be noted that we could not exclude that other receptors may be associated with antipsychotic-induced NLRP3/caspase-1 signaling activation. Haloperidol has low affinity for the H1 receptor, but it did activate NLRP3/caspase-1 signaling. In astrocytes, D2 receptor antagonism induced astrocyte inflammation (Shao et al., 2013), while D2 receptor activation suppressed astrocyte inflammation and restricted NLRP3 inflammasome and caspase-1 activation (Zhu et al., 2018). Olanzapine, quetiapine, risperidone, or haloperidol all have high affinity for the D2 receptor. These findings suggest that antipsychotic-induced activation of NLRP3/caspase-1 signaling in astrocytes may also be related to the D2 receptor. However, it is well known that the D2 receptor is associated with the therapeutic effects of antipsychotics (Seeman, 2002). Therefore, the D2 receptor may not be an ideal target to treat antipsychotic-induced astrocyte death. In the present study, although haloperidol may work on the D2 receptor rather than the H1 receptor to activate NLRP3/caspase-1 signaling, activation of the H1 receptor by FMPH might offset part of the effect of haloperidol on NLRP3/caspase-1 signaling. It is also possible that FMPH-induced inhibition of NLRP3 signaling is an indirect effect during haloperidol treatment.

Furthermore, we have demonstrated the involvement of NLRP3 signaling in antipsychotic-induced astrocyte death. Recent findings have revealed that pharmacological blockade of NLRP3 signaling by using salidroside, Dl-3-n-butylphthalide (NBP), gliquidone, or dimethyl fumarate significantly inhibited astrocyte-mediated neuroinflammation, reduced mitochondrial impairment, improved neuronal survival, and slowed the progression of various disease (Cai et al., 2021; Kim et al., 2021; Que et al., 2021; Tastan et al., 2021). Therefore, we suggest that inhibition of NLRP3 signaling may help suppress antipsychotic-induced inflammation and astroglia death. Future studies could investigate whether NLRP3 inhibitors such as MCC950, NBP, and gliquidone could inhibit antipsychotic-induced astrocyte death, providing potential therapeutic agents to counteract antipsychotic-induced brain cell death.

The limitation of this study was that we did not examine the effects of antipsychotics on NLRP3/caspase-1 signaling *in vivo*. In the future, we will investigate the chronic effects of olanzapine, quetiapine, risperidone and haloperidol on the NLRP3/caspase-1 signaling, inflammatory factors such as IL-1β, and astrocyte survival in the cortex as well as other brain regions of rodents.

## Conclusion

Treatment with olanzapine, quetiapine, risperidone, or haloperidol time-dependently affected the viability of cultured astrocytes. During chronic treatment, antipsychotics induced astrocyte death and dose-dependently activated NLRP3/caspase-1 signaling. These effects may be partly responsible for antipsychotic induced brain volume loss. Co-treatment with a histamine H1 receptor agonist reduced the antipsychotic-induced activation of NLRP3/caspase-1 signaling, suggesting that H1 receptor activation may be an effective strategy to ameliorate antipsychotic-induced inflammation, astrocyte death, and brain volume loss. This study may help develop strategies to inhibit antipsychotic-induced astrocyte death and to develop novel antipsychotic drugs with fewer inflammatory and toxic side effects.

## Data Availability Statement

The original contributions presented in the study are included in the article/Supplementary Material, further inquiries can be directed to the corresponding authors.

## Author Contributions

MH, TS, and YL designed the study. MH, GG, RZ, JF, YZ, RL, and BL performed the experiments and managed the project. MH, JF, and RZ undertook the statistical analysis and wrote the first draft of the manuscript. MH and TS contributed to the interpretation of the results and revised the manuscript. All authors have contributed to and have approved the final manuscript.

## Conflict of Interest

The authors declare that the research was conducted in the absence of any commercial or financial relationships that could be construed as a potential conflict of interest.

## Publisher’s Note

All claims expressed in this article are solely those of the authors and do not necessarily represent those of their affiliated organizations, or those of the publisher, the editors and the reviewers. Any product that may be evaluated in this article, or claim that may be made by its manufacturer, is not guaranteed or endorsed by the publisher.

## Supplementary Material

Supplementary Figure 1 Treatment with 1, 5, 10, or 20 μM 2-(3-trifluoromethylphenyl) histamine (FMPH) did not affect astrocyte viability.

Supplementary Figure 2 Treatment with 5 or 10 μM 2-(3-trifluoromethylphenyl) histamine (FMPH) did not affect NLRP3 protein expression in cultured astrocytes. Cultured cells were treated with vehicle (water), FMPH 5 μM, or FMPH 10 μM for 24 h. Western blot was used to investigate NLRP3 protein expression. The data are presented as the mean ± standard error of the mean. *p* < 0.05 (one-way analysis of variance and *post hoc* Dunnett’s multiple comparison test; *n* = 4 samples/group).
