# Supplementary material for: NLRP3/Caspase-1-Mediated Pyroptosis of Astrocytes Induced by Antipsychotics Is Inhibited by a Histamine H1 Receptor-Selective Agonist
Source: Front Aging Neurosci. 2022 May 9;14:847561. doi: 10.3389/fnagi.2022.847561 (PMC9125084; doi:10.3389/fnagi.2022.847561)

1. **Supplementary figure 1.**

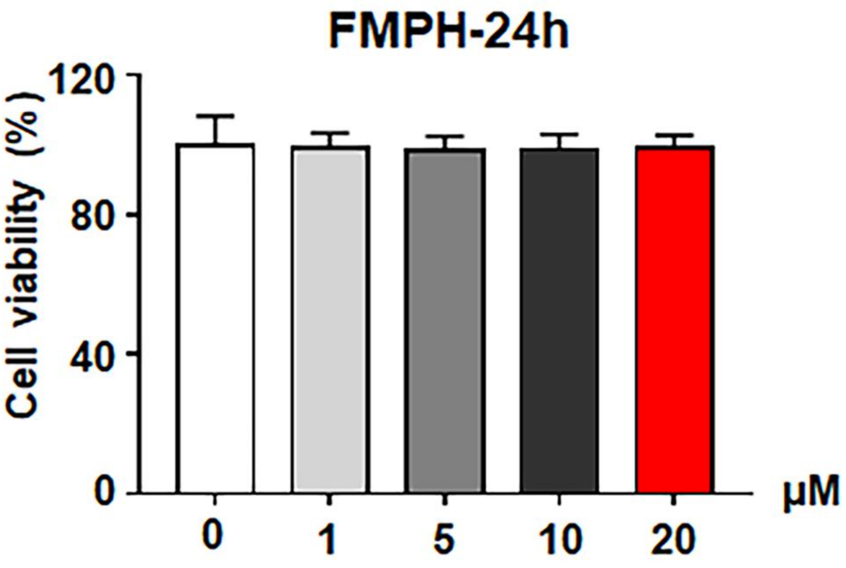

2. **Supplementary figure 2.**

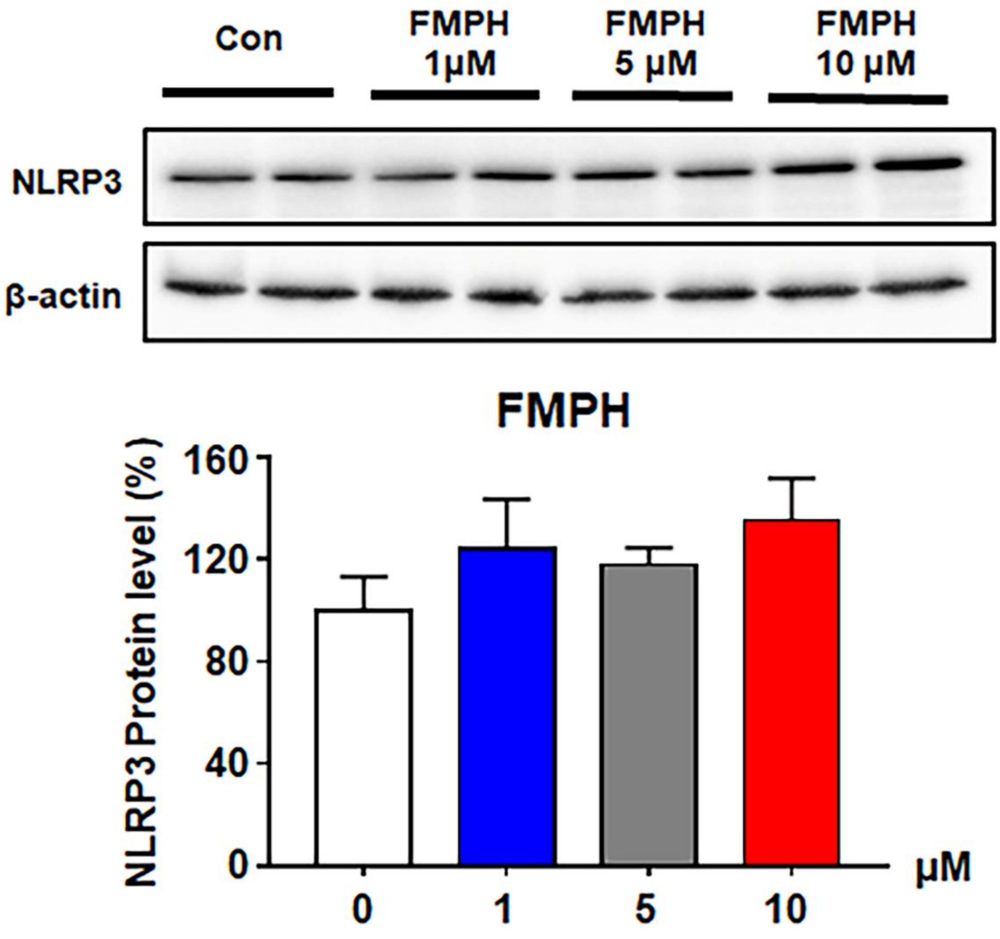

3. **Supplementary figure 3. Original images in figure 2A and D**

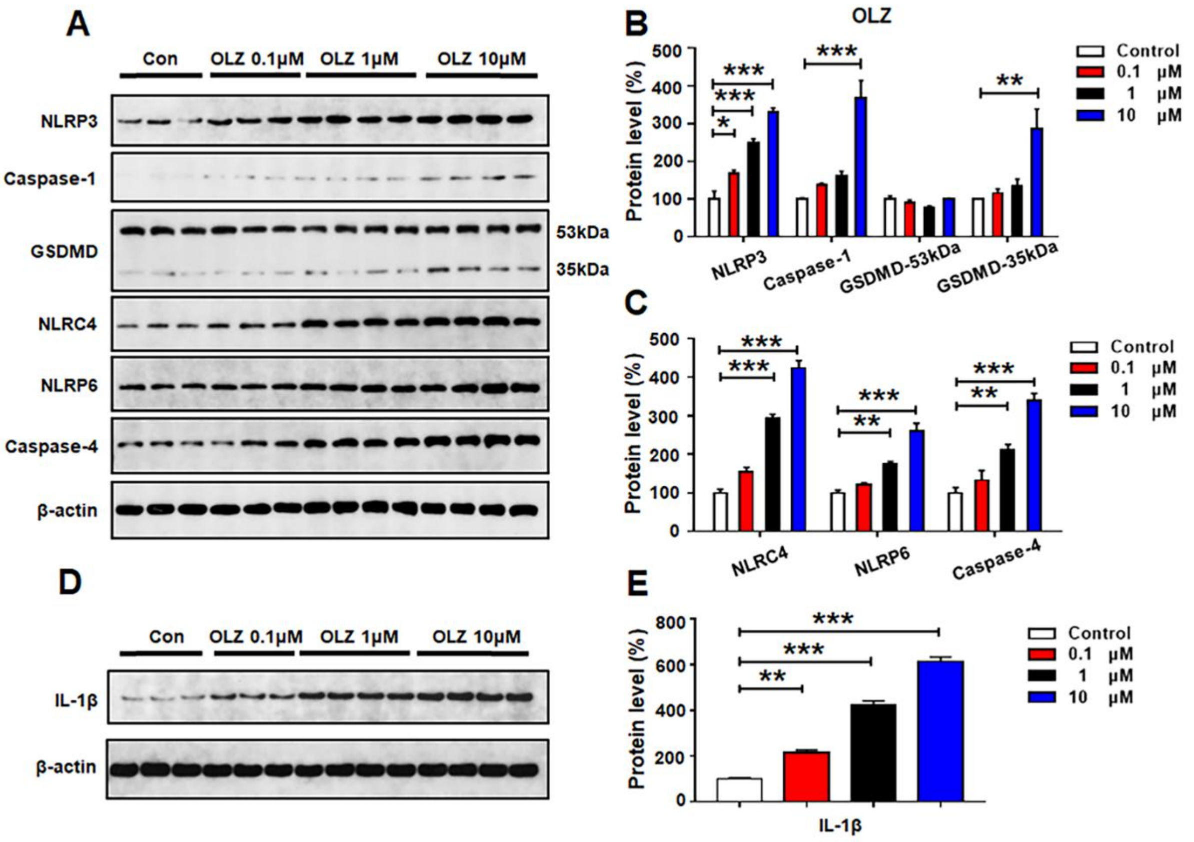

3.1 OLZ-NLRP3

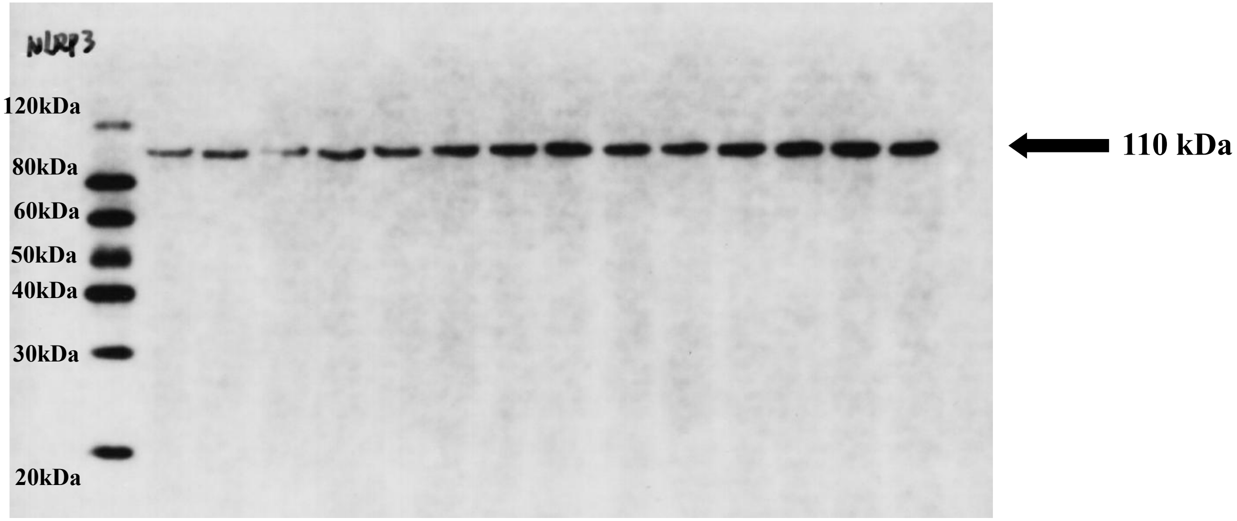

3.2 OLZ-Caspase1

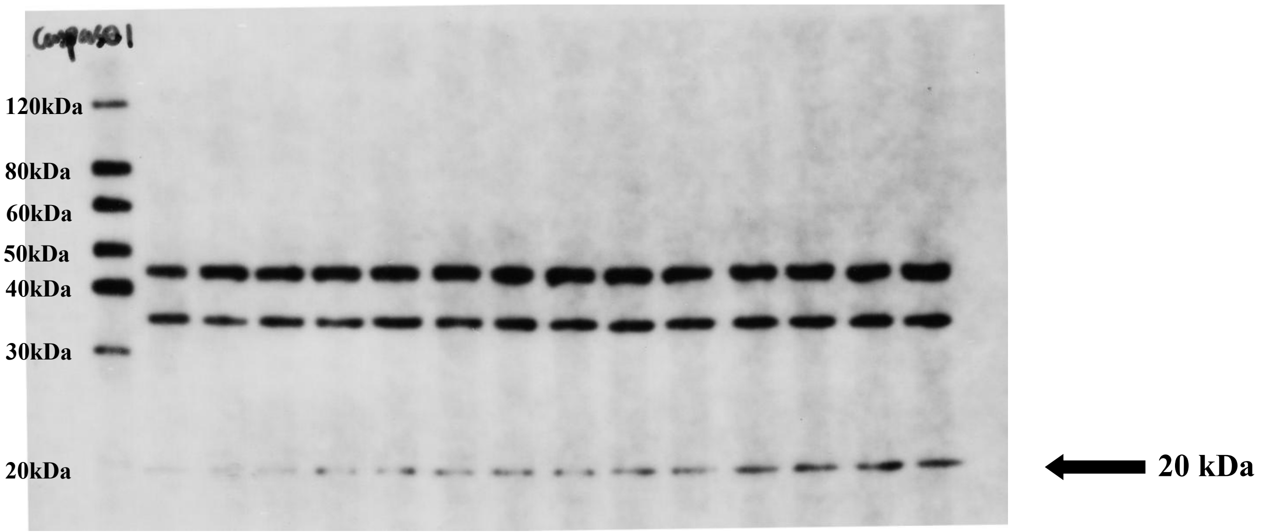

3.3 OLZ-GSDMD

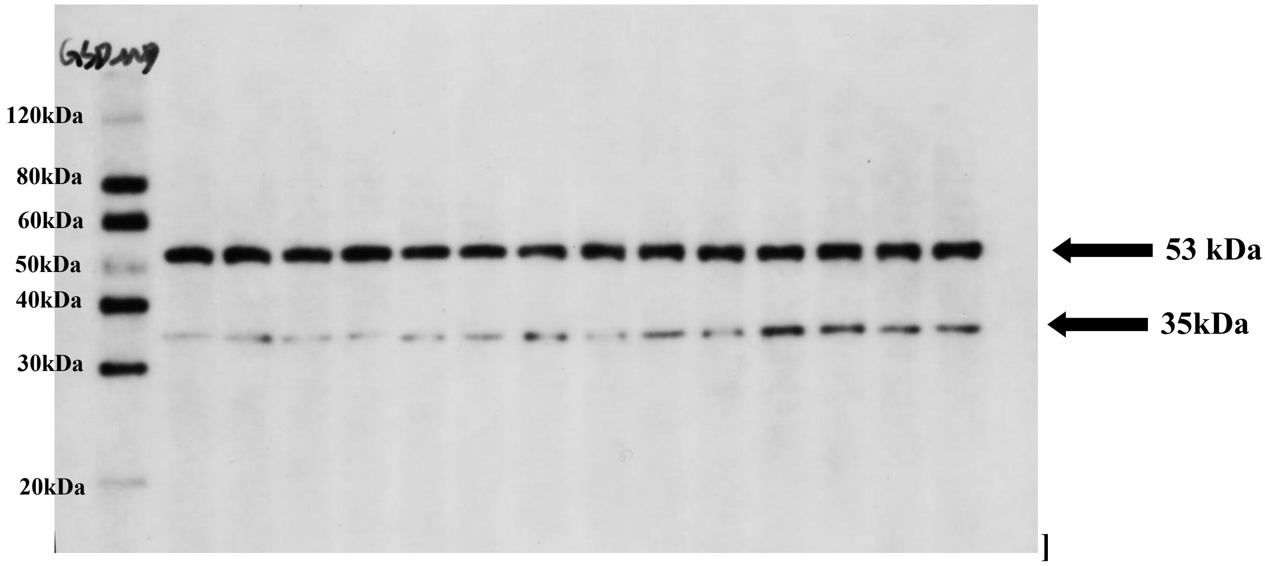

3.4 OLZ-NLRC4

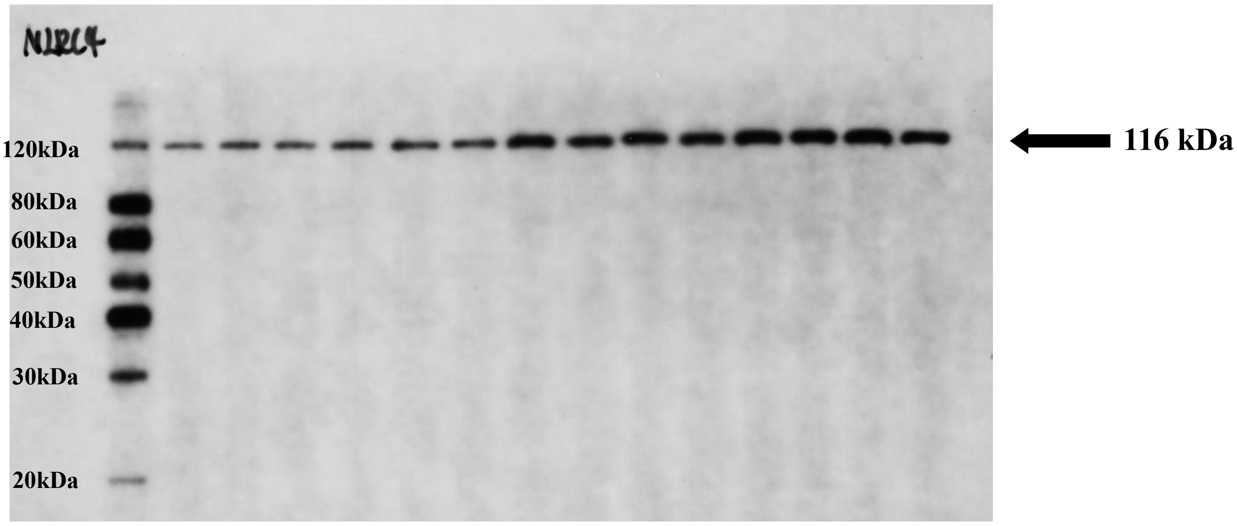

3.5 OLZ-NLRP6

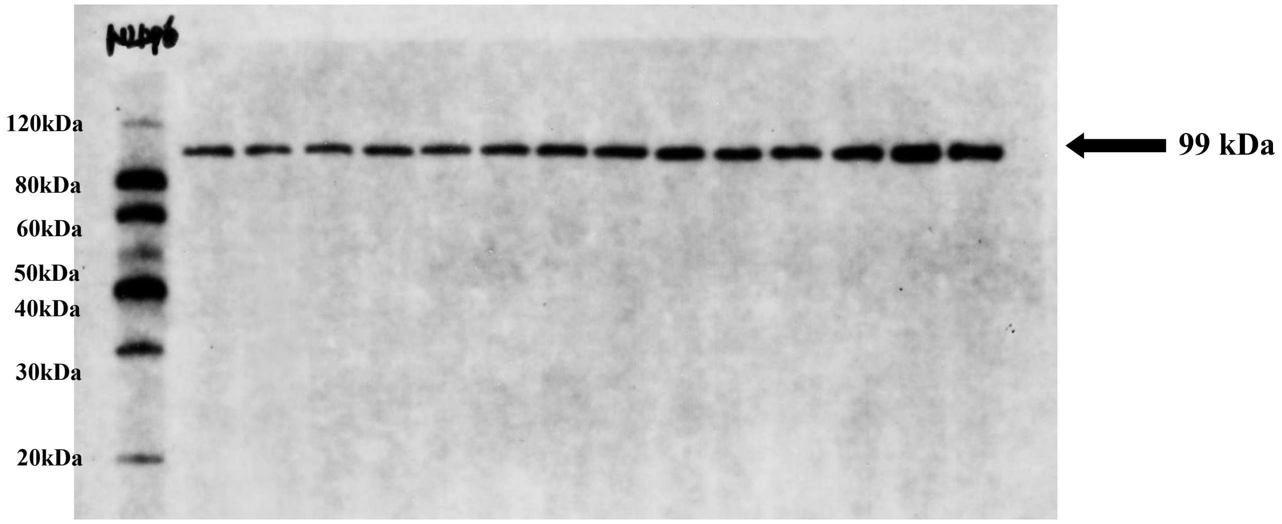

3.6 OLZ-Caspase4

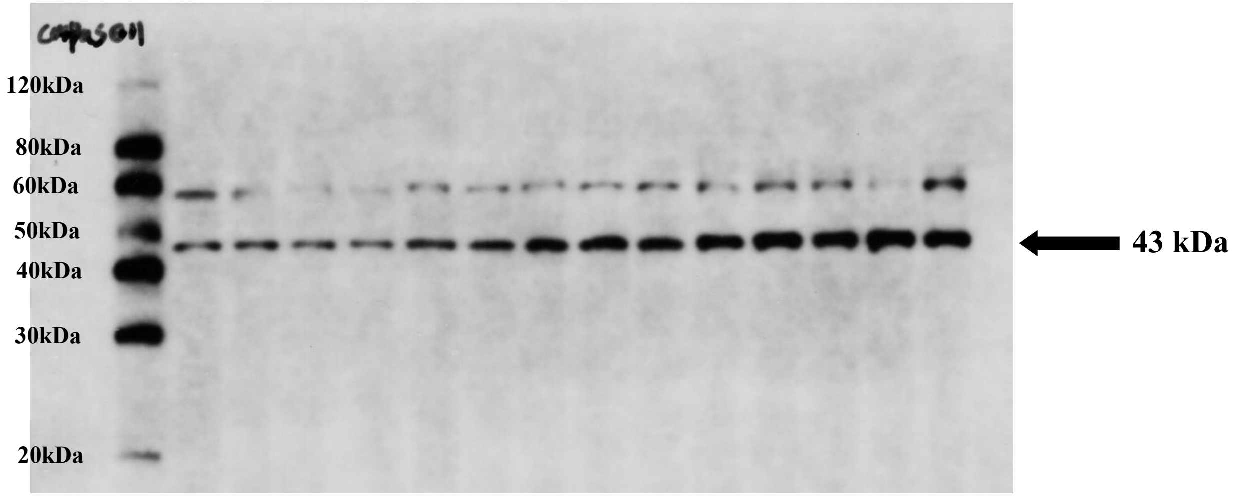

3.7 OLZ-β-actin

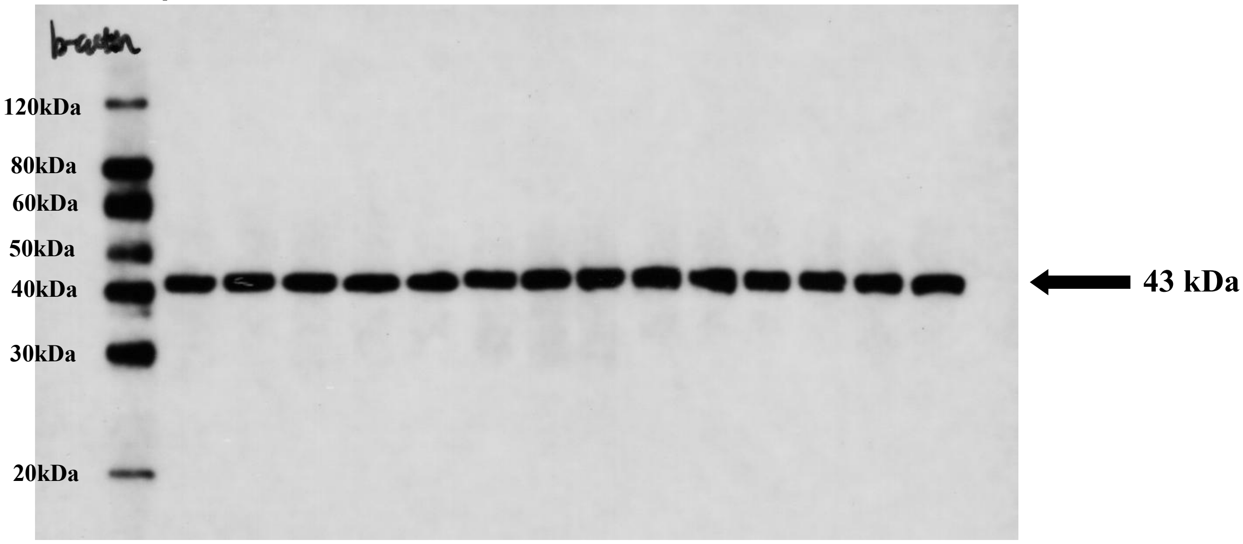

3.8 OLZ-IL-1β

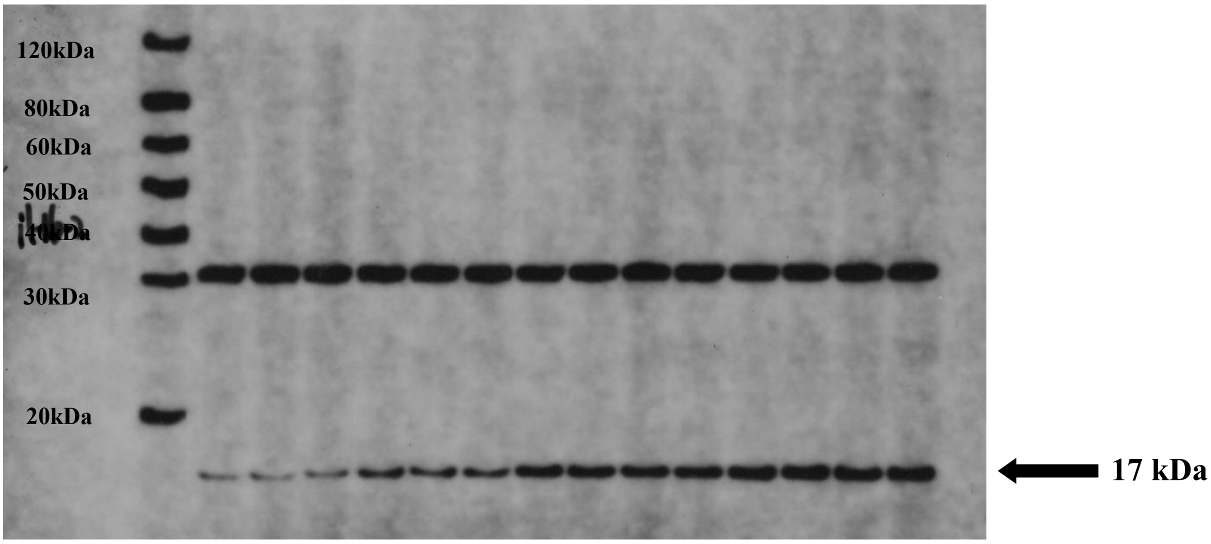

3.9 OLZ-β-actin

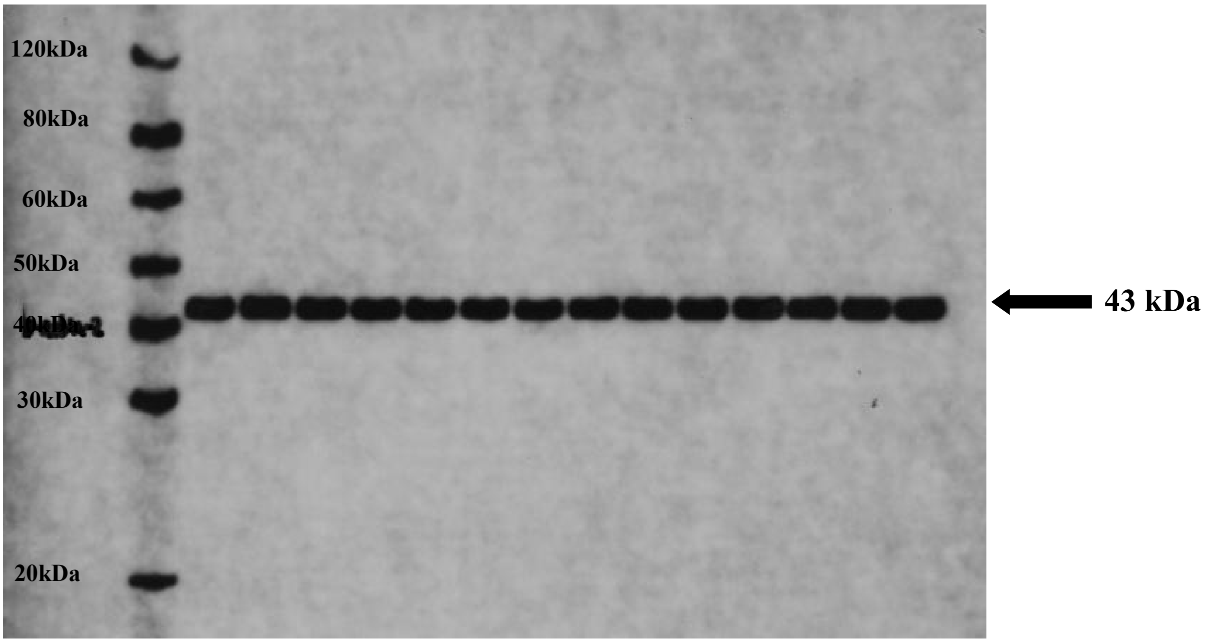

4. Supplementary figure 4. Original images in figure 3A and D

4.1QUE-NLRP3

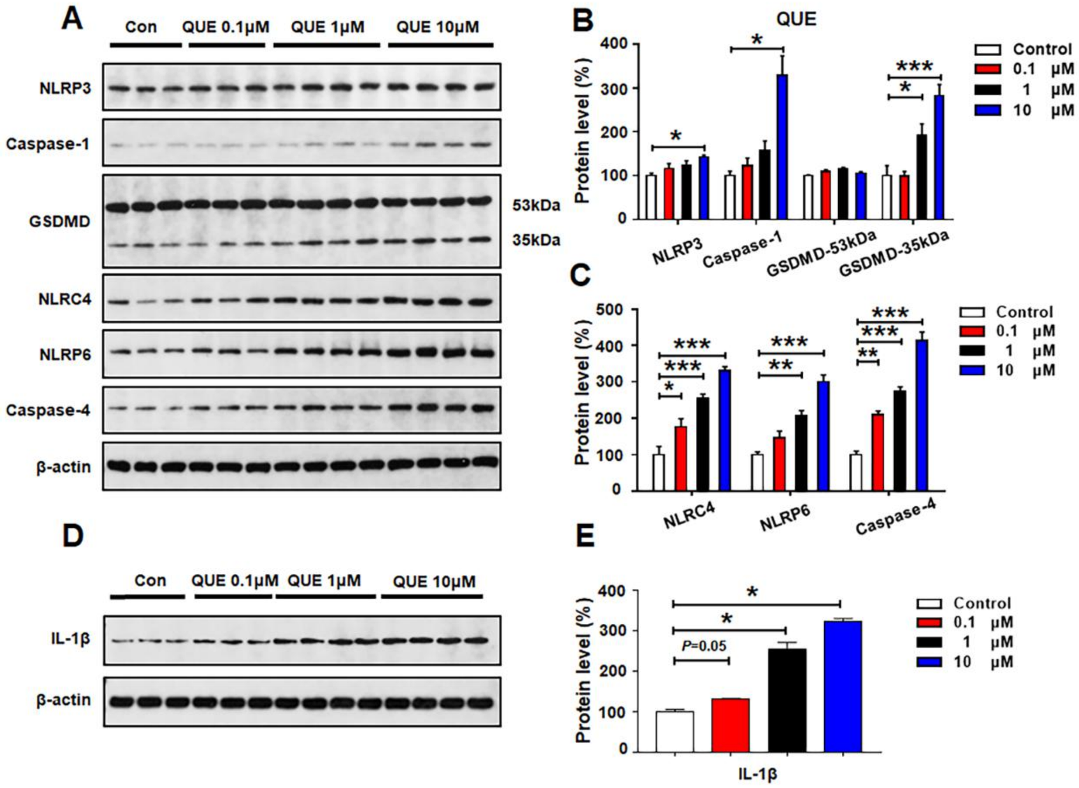

110 kDa

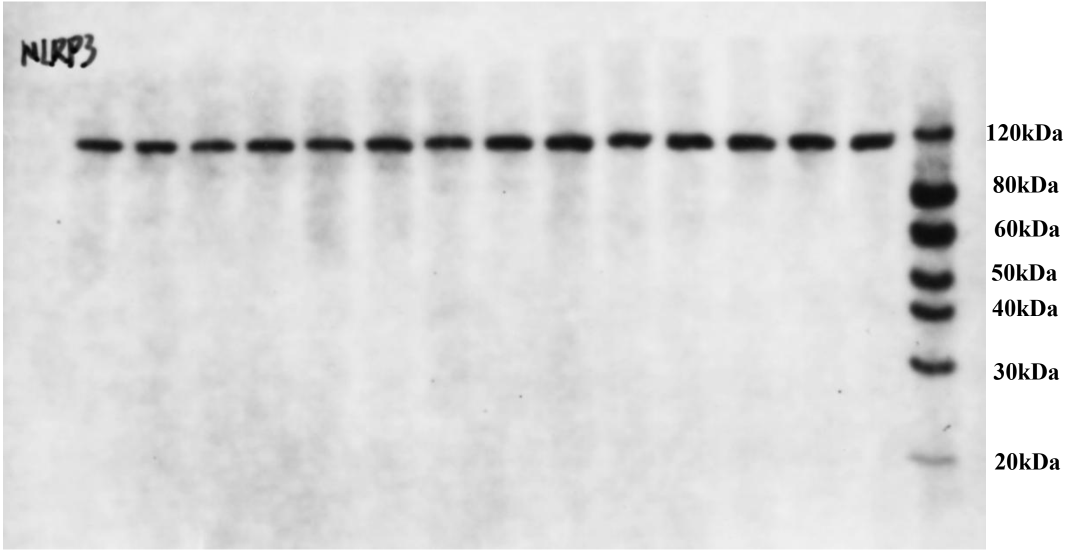

4.2 QUE-Caspase1

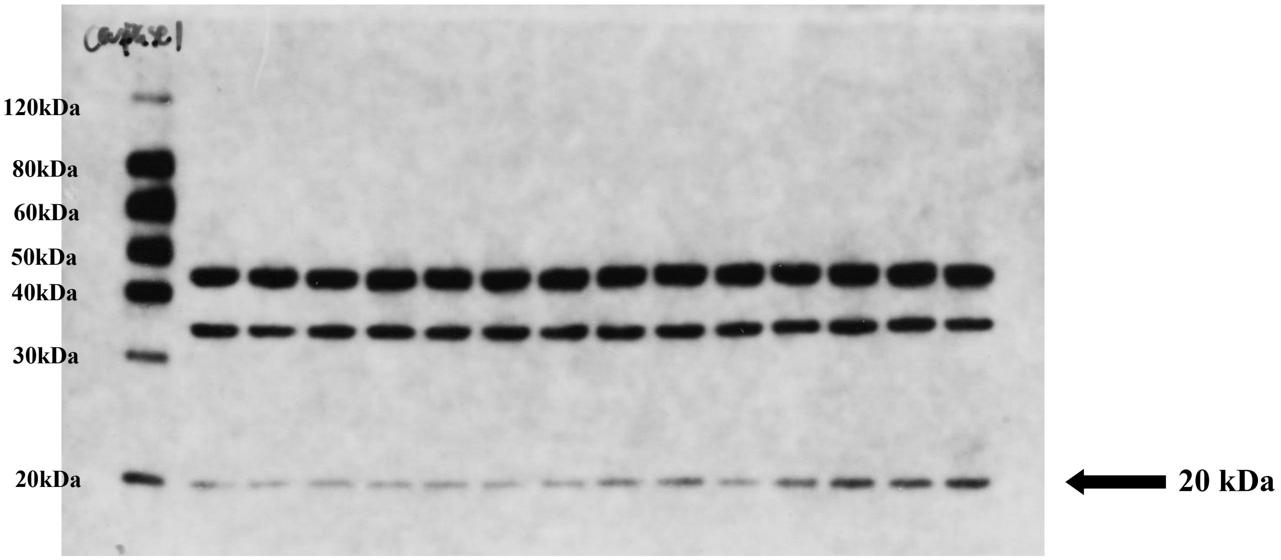

4.3QUE-GSDMD

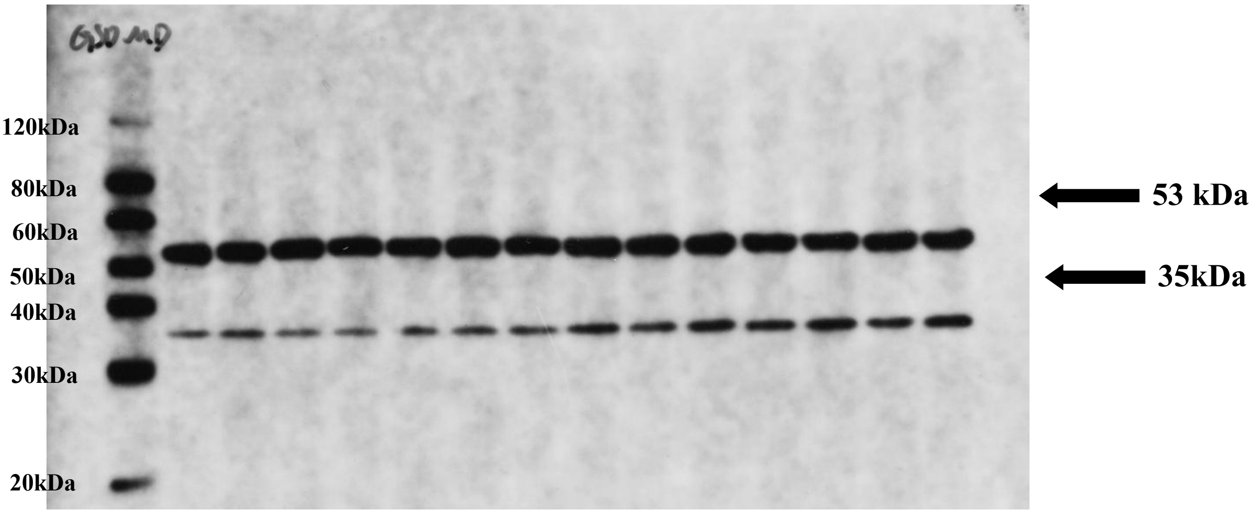

4.4QUE-NLRC4

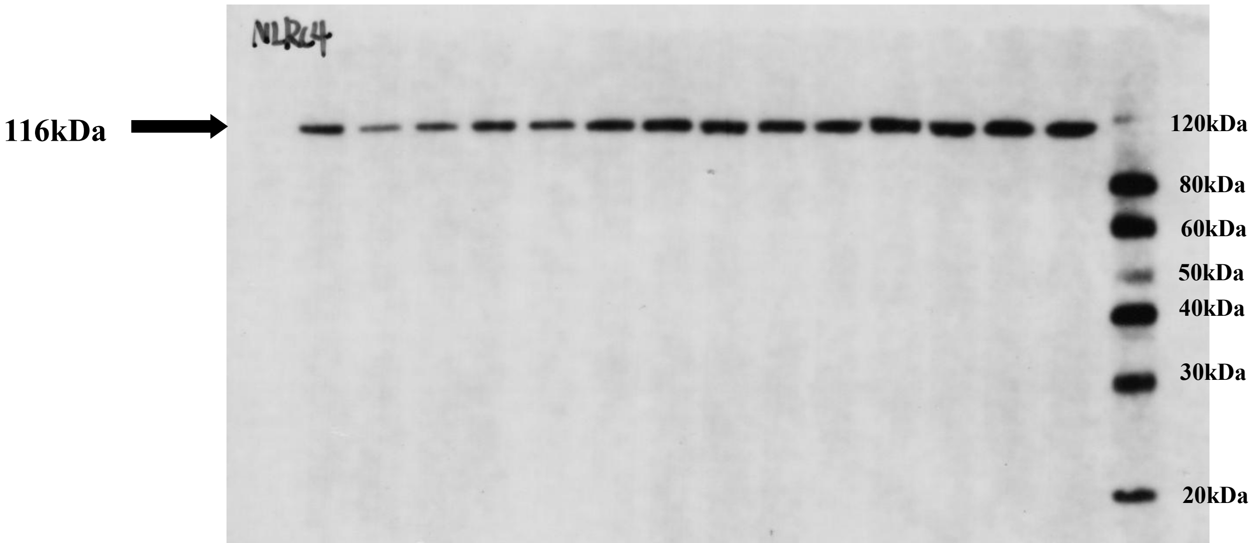

4.5 QUE-NLRP6

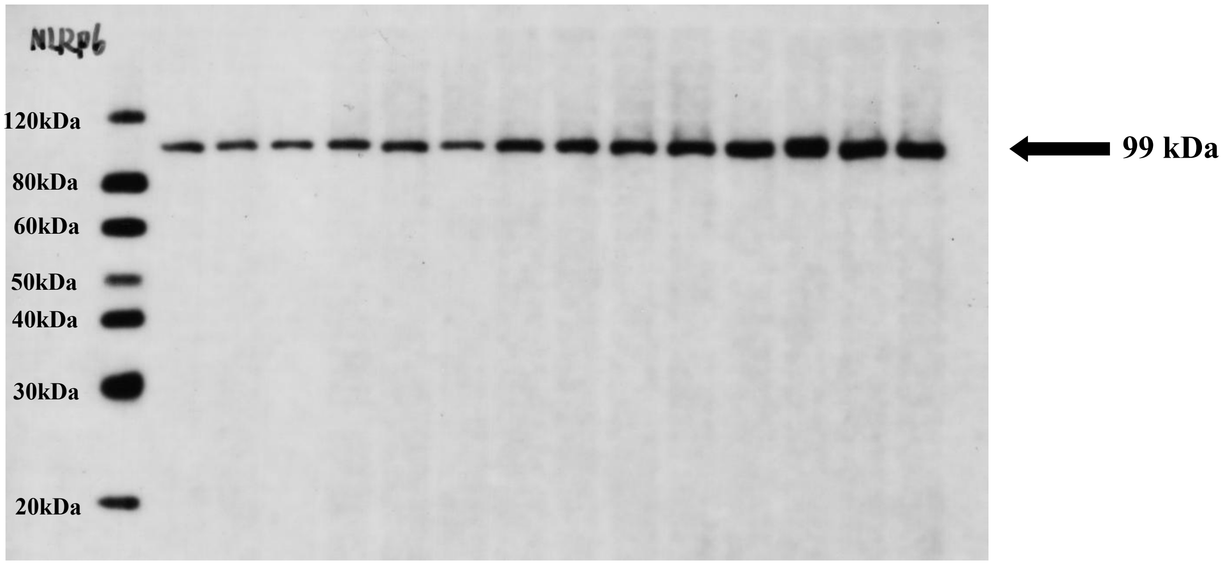

4.6 QUE-Caspase4

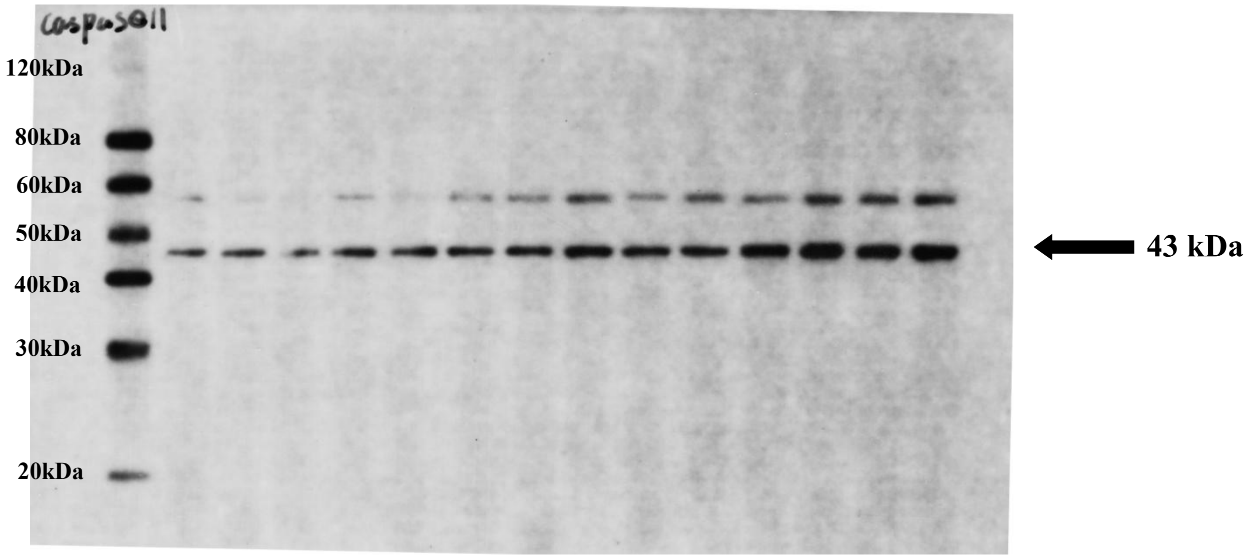

4.7 QUE-Actin

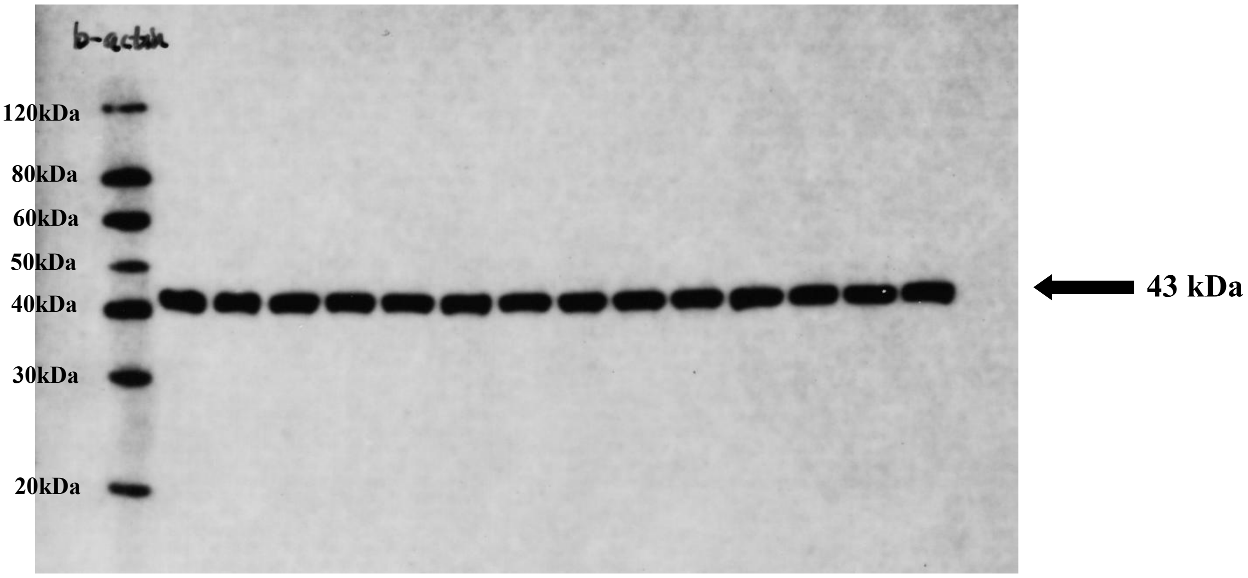

4.8 QUE-IL-1β

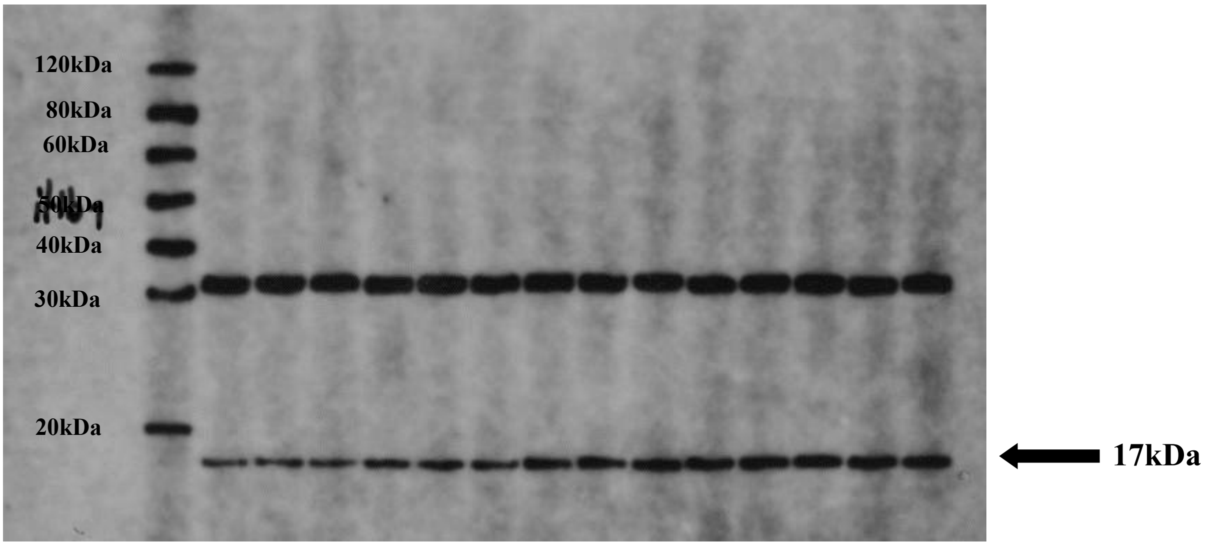

4.9 QUE-β-actin

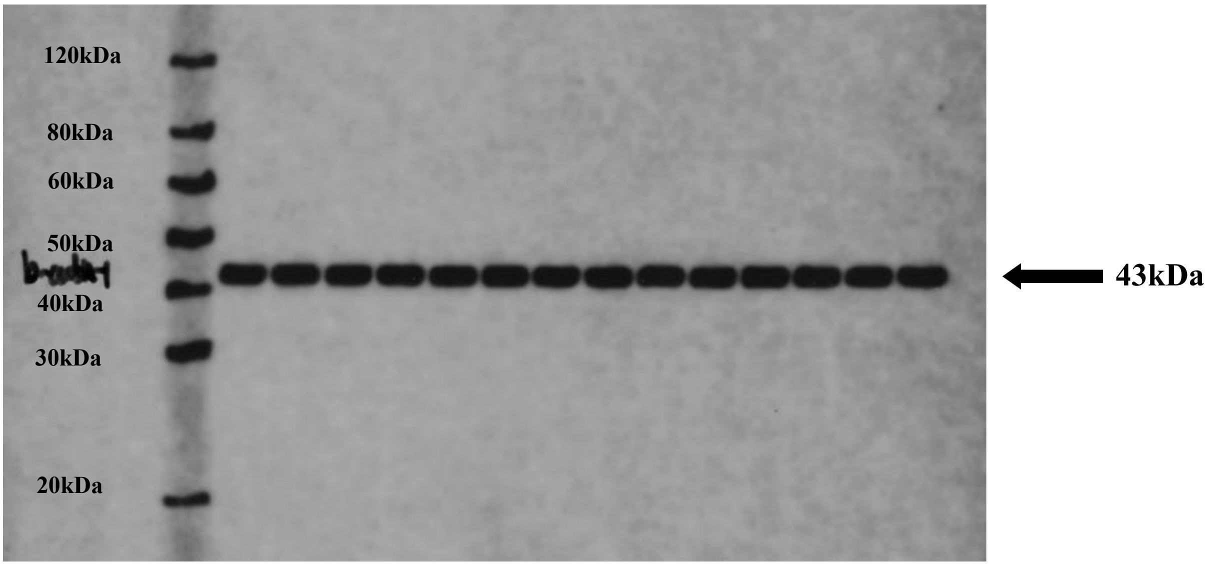

5. Supplementary figure 5. Original images in figure 4A and D

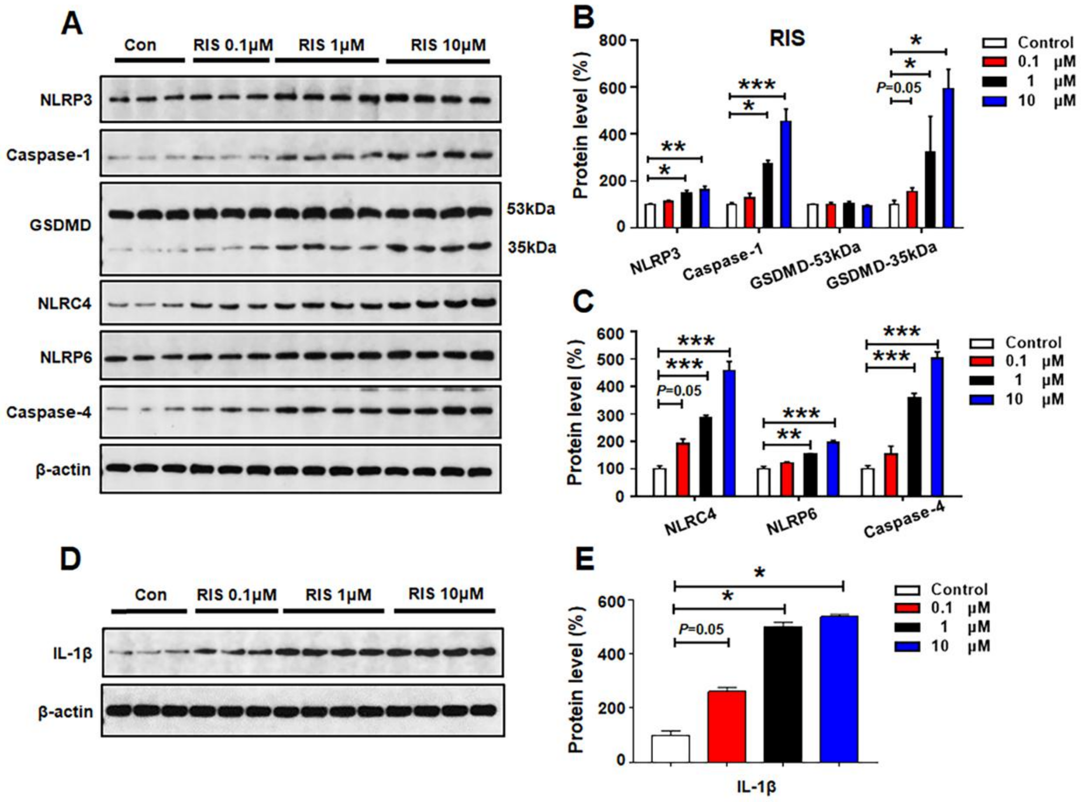

5.1 RIS-NLRP3

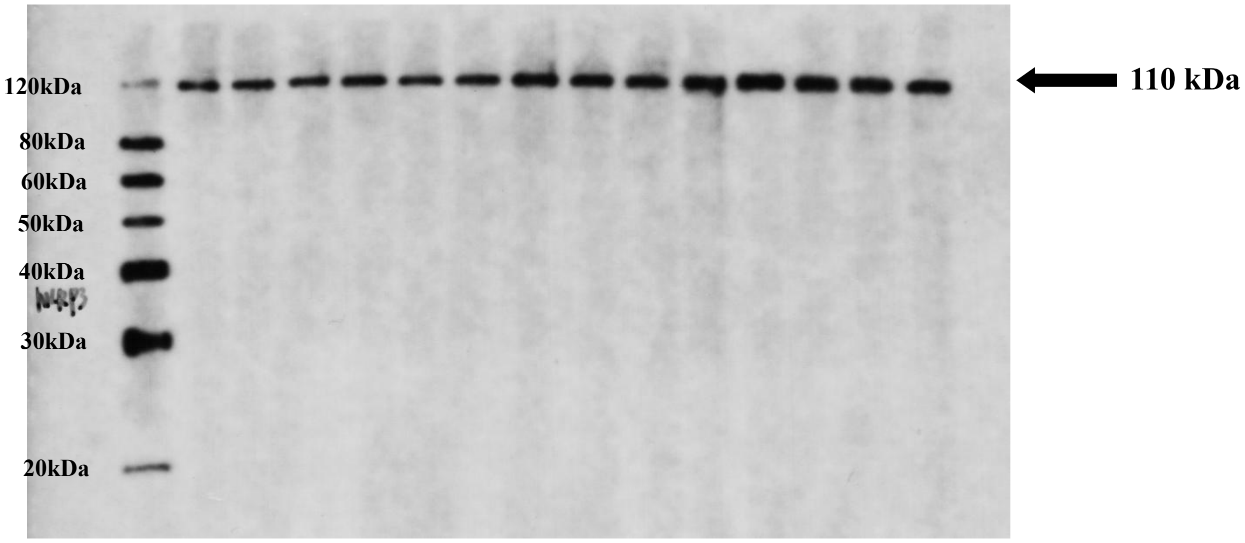

5.2 RIS-Caspase1

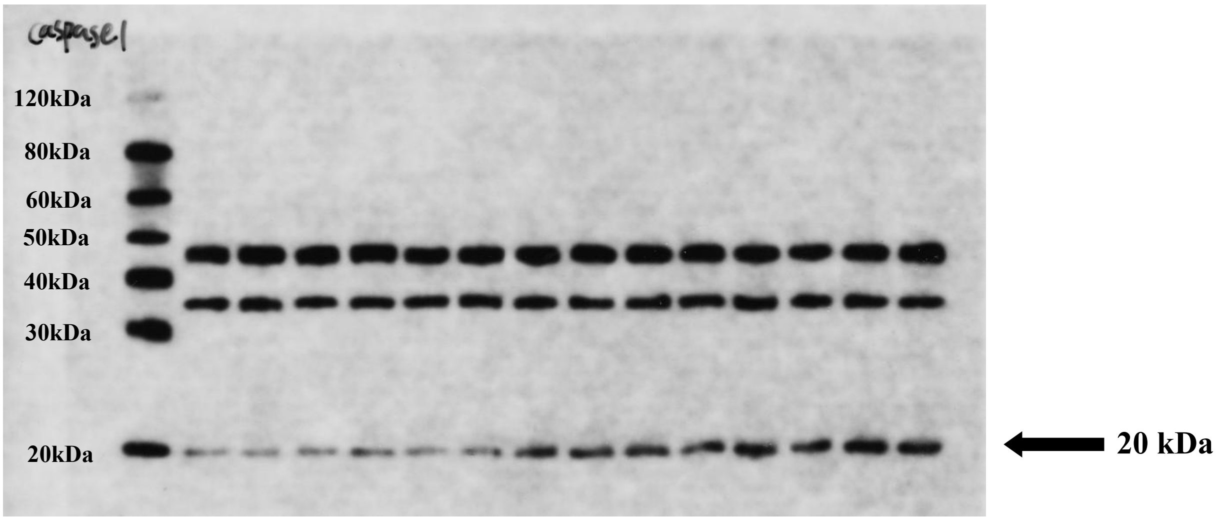

5.3 RIS-GSDMD

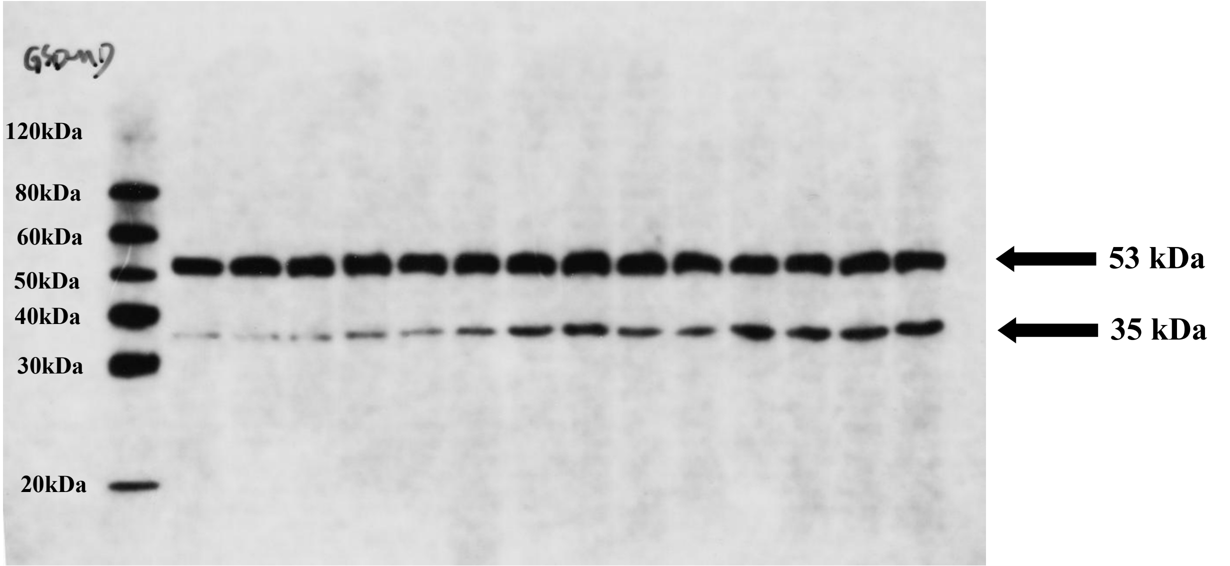

5.4 RIS-NLRC4

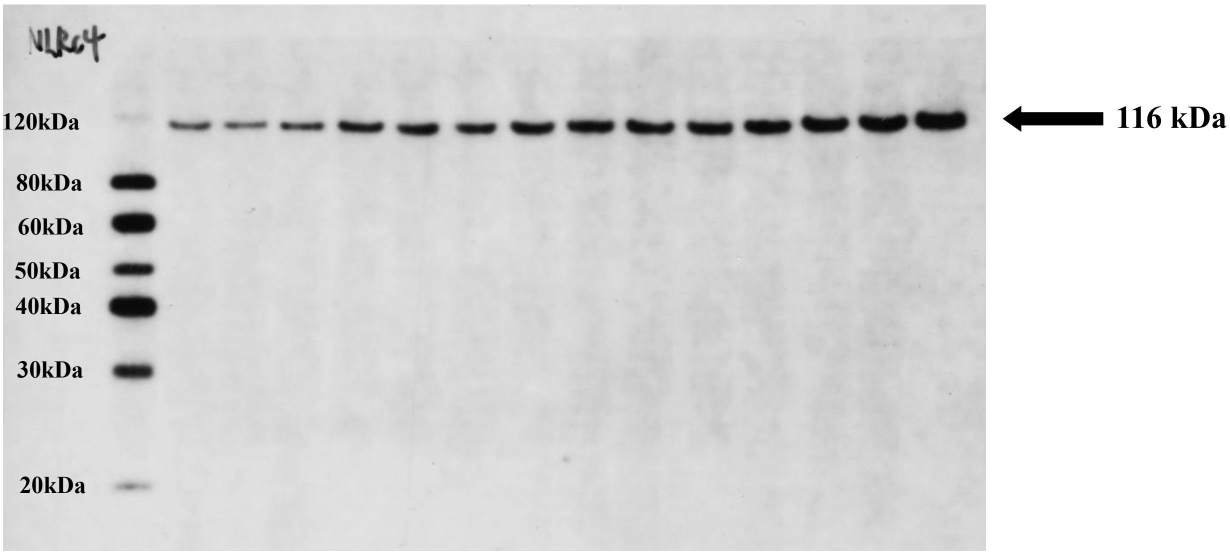

5.5 RIS-NLRP6

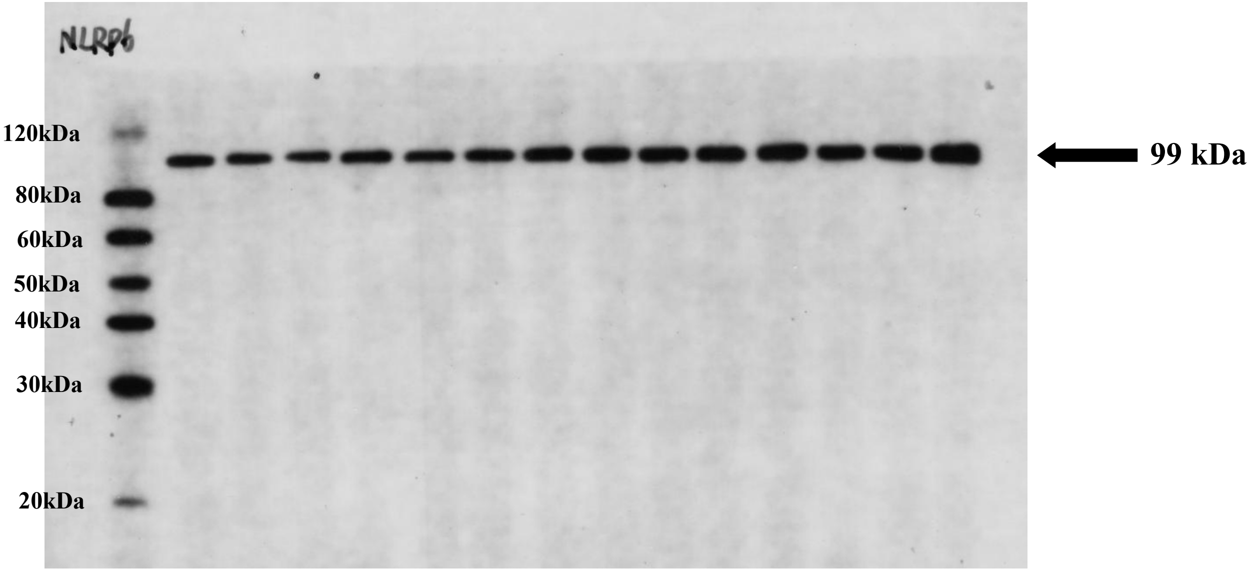

5.6 RIS-Caspase4

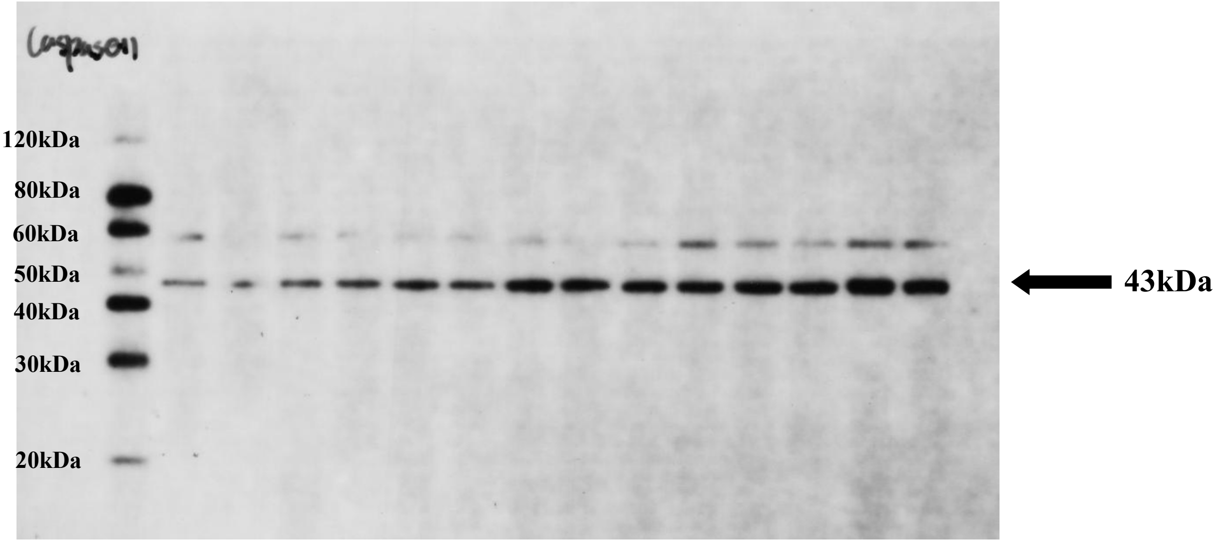

5.7 RIS-Actin

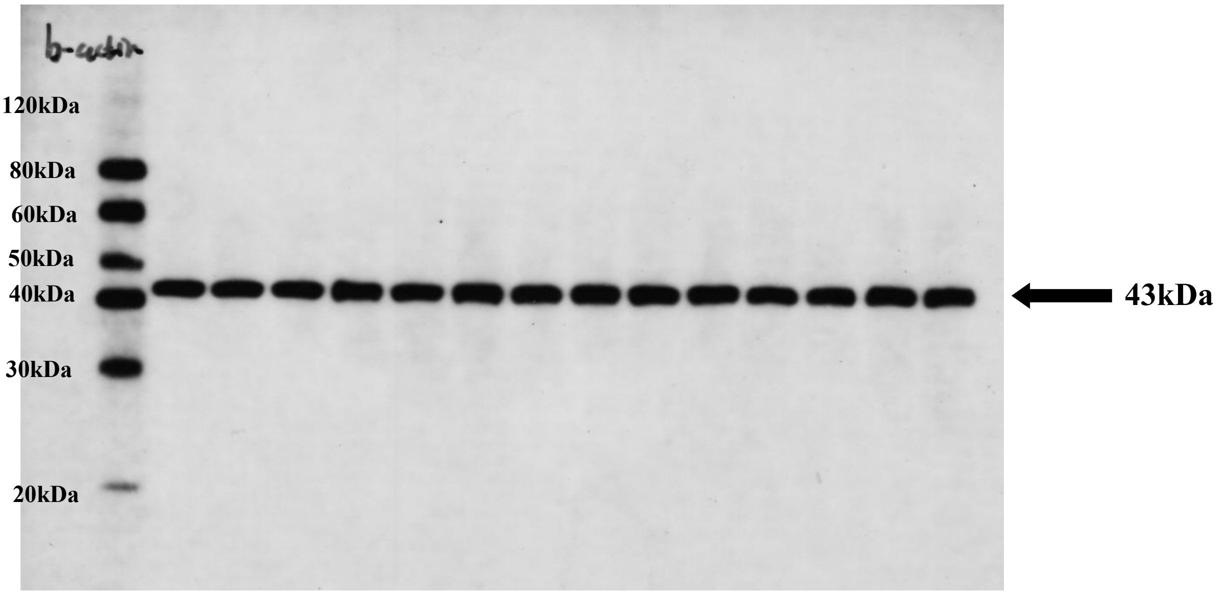

5.8 RIS-IL-1 β

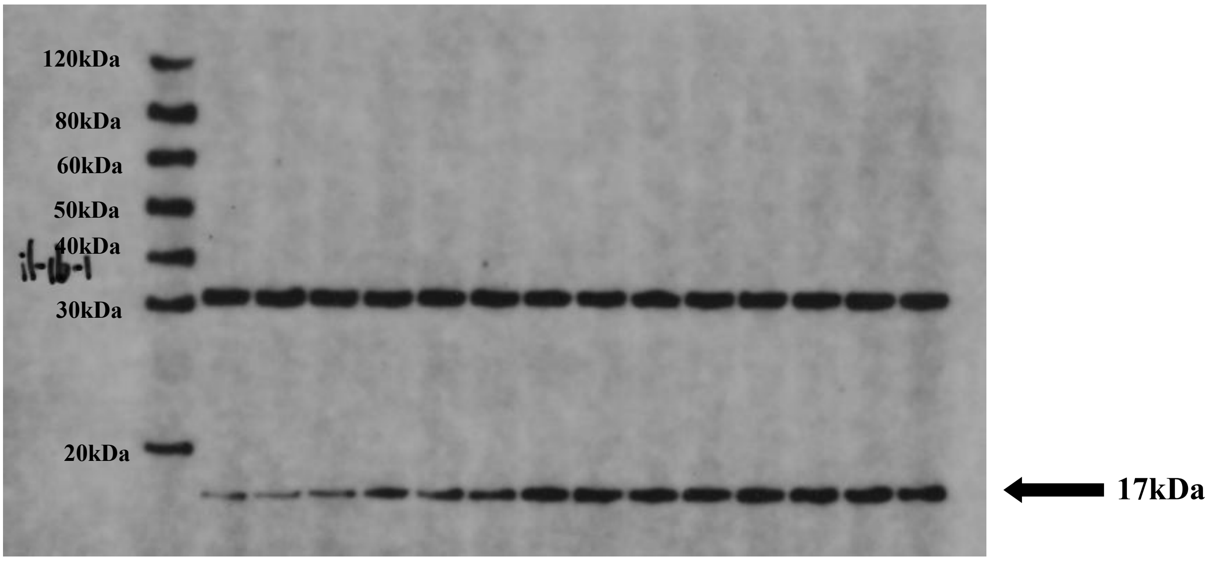

5.9 RIS- β -actin

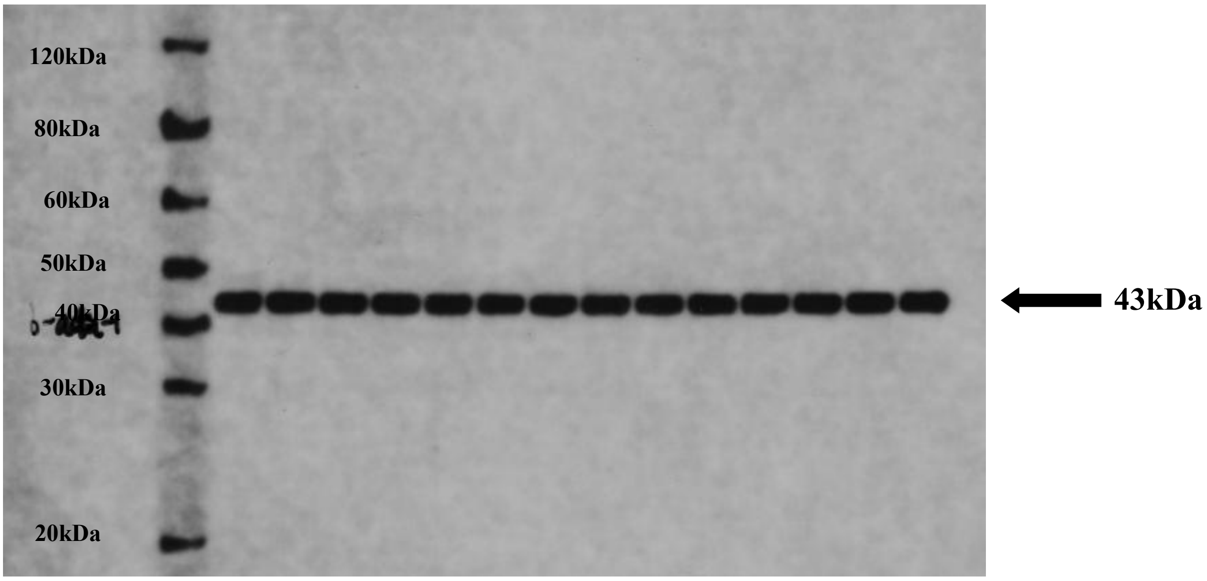

6. Supplementary figure 6. Original images in figure 5A and D

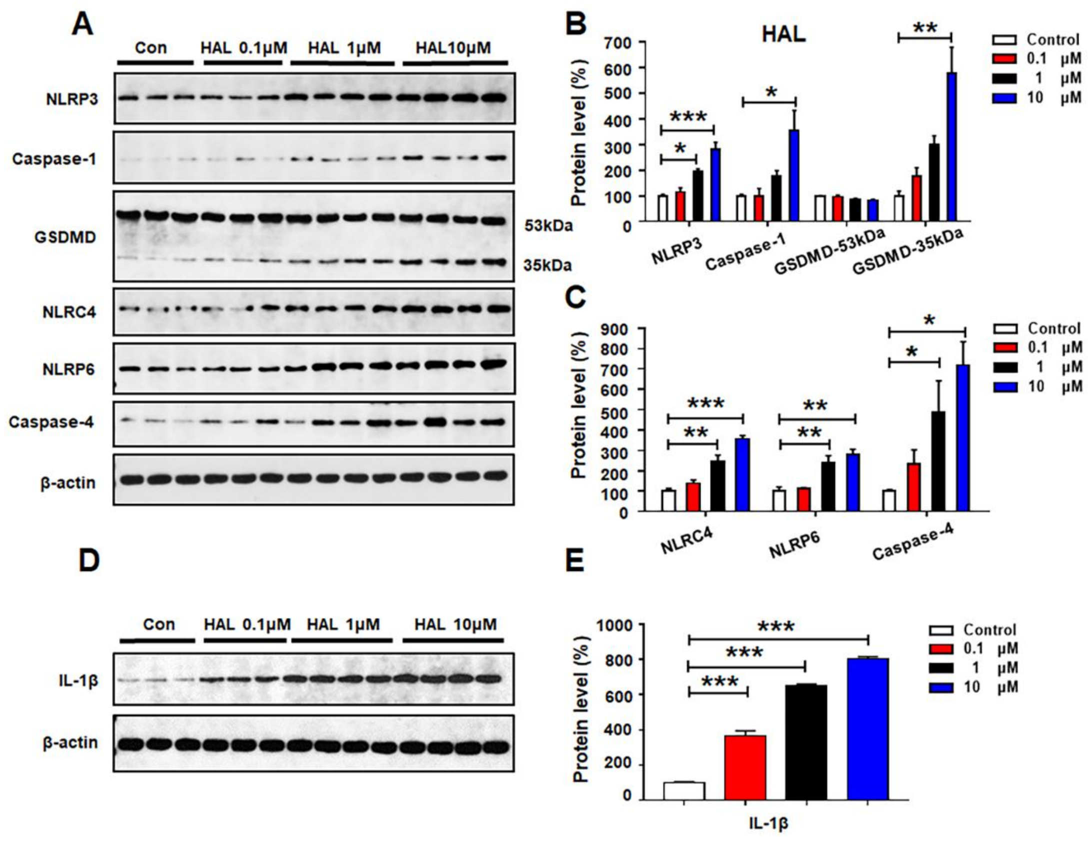

6.1 HAL-NLRP3

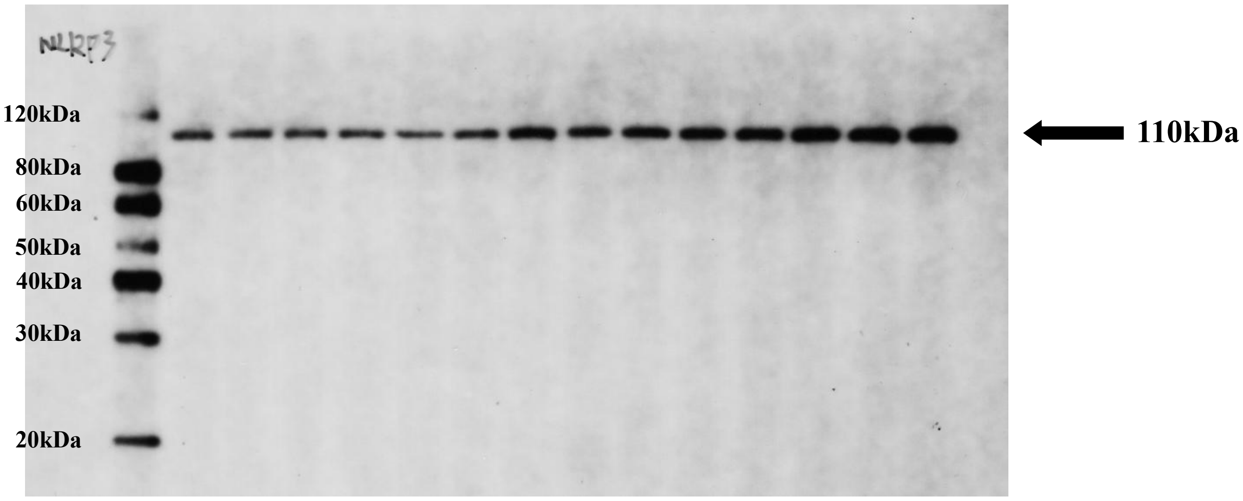

6.2 HAL-Caspase1

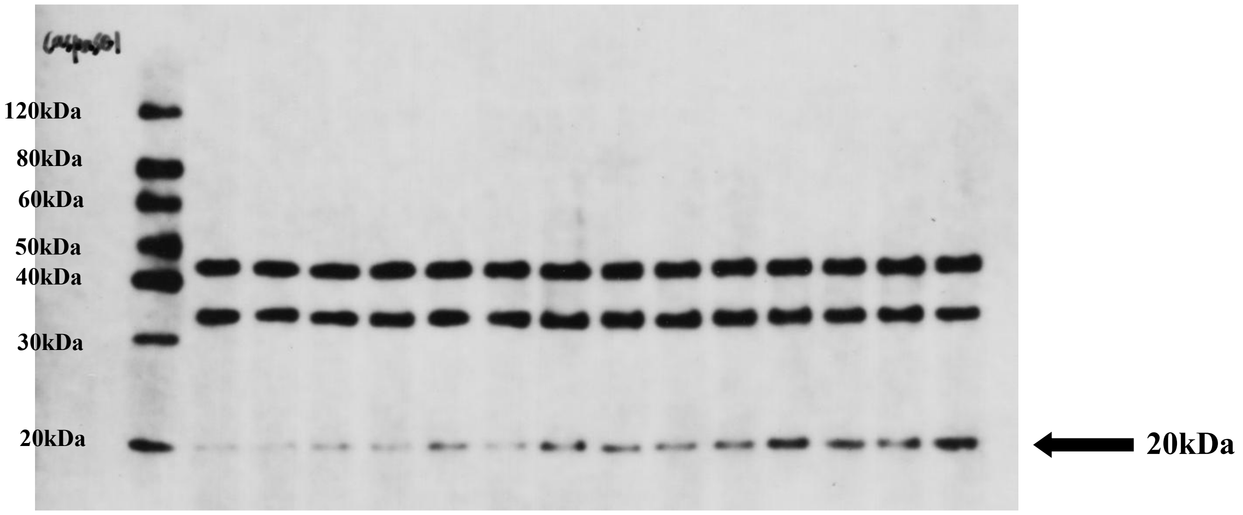

6.3 HAL-GSDMD

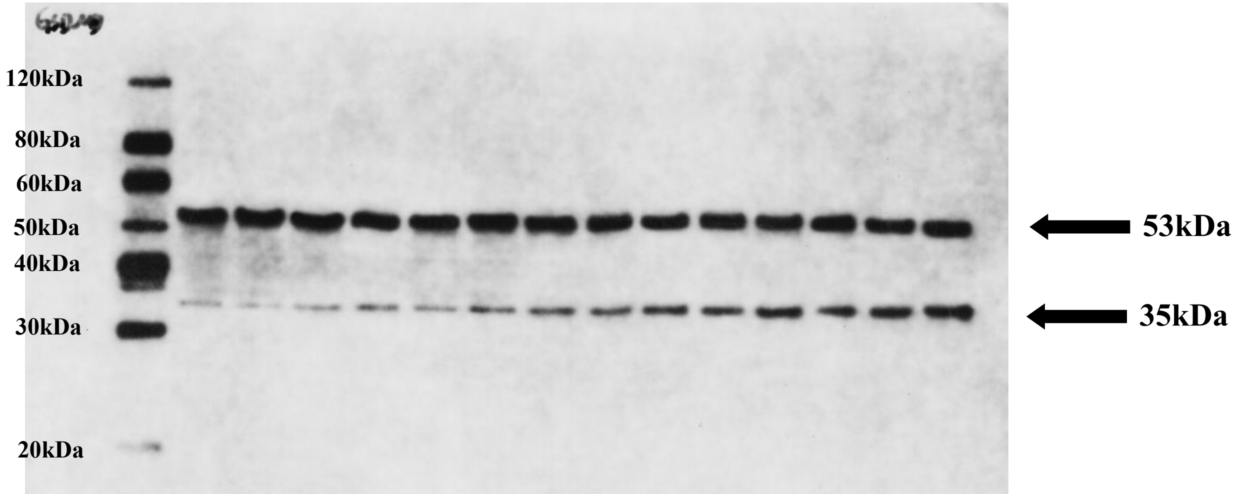

6.4 HAL-NLRC4

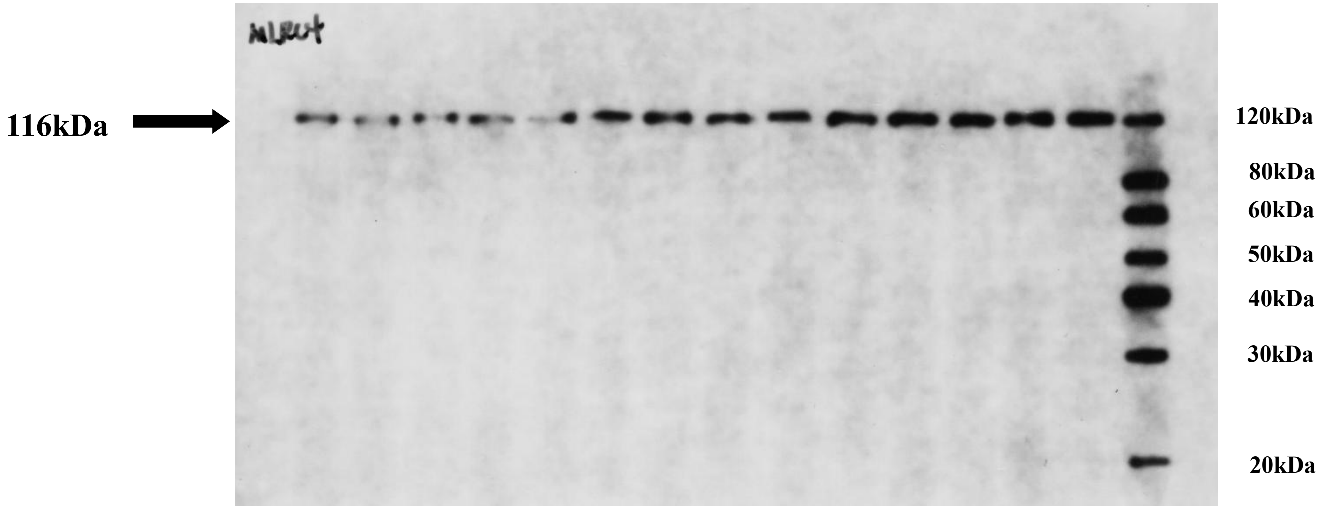

6.5 HAL-NLRP6

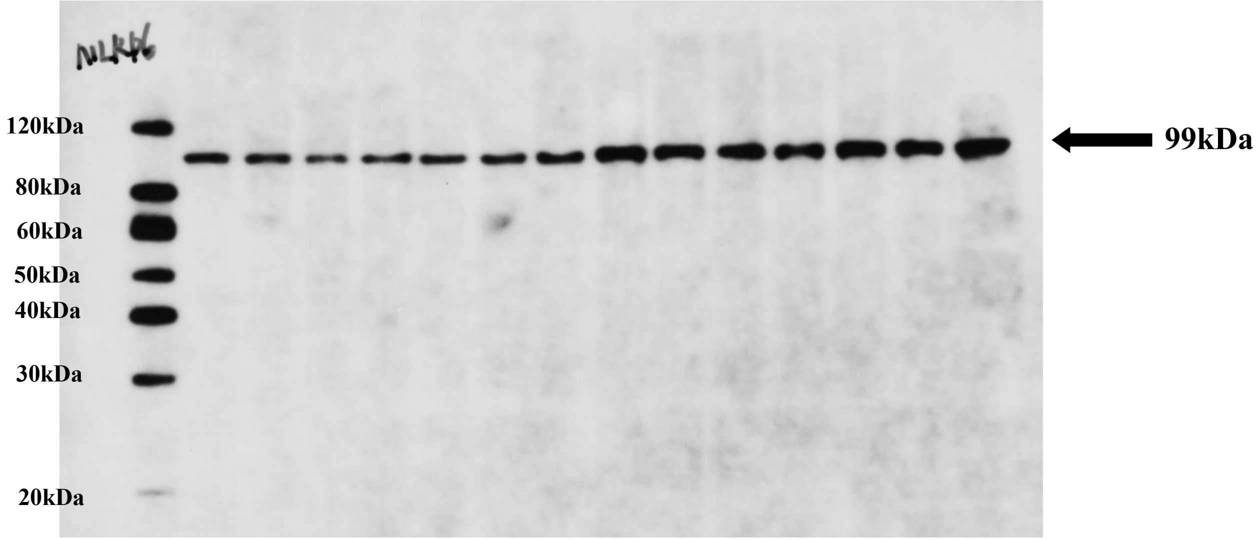

6.6 HAL-Caspase4

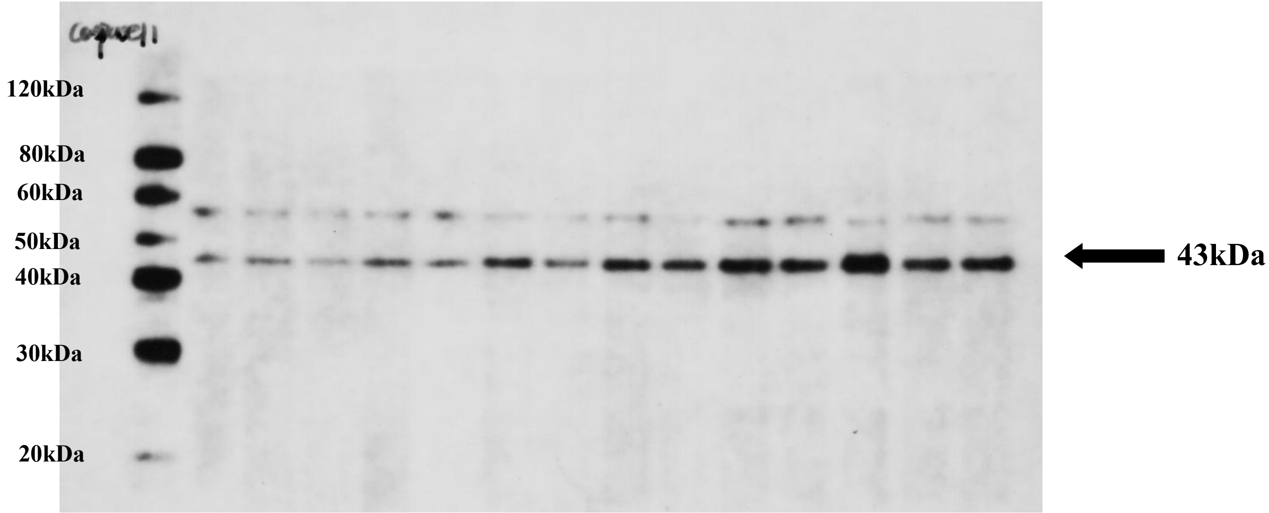

6.7 HAL-β-actin

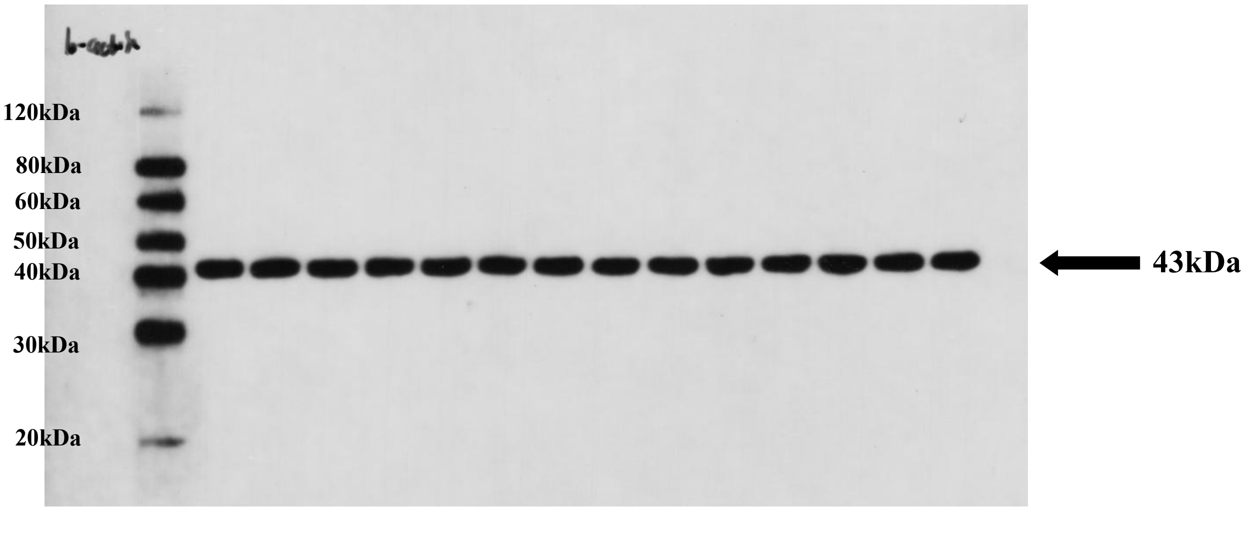

6.8 HAL-IL-1β

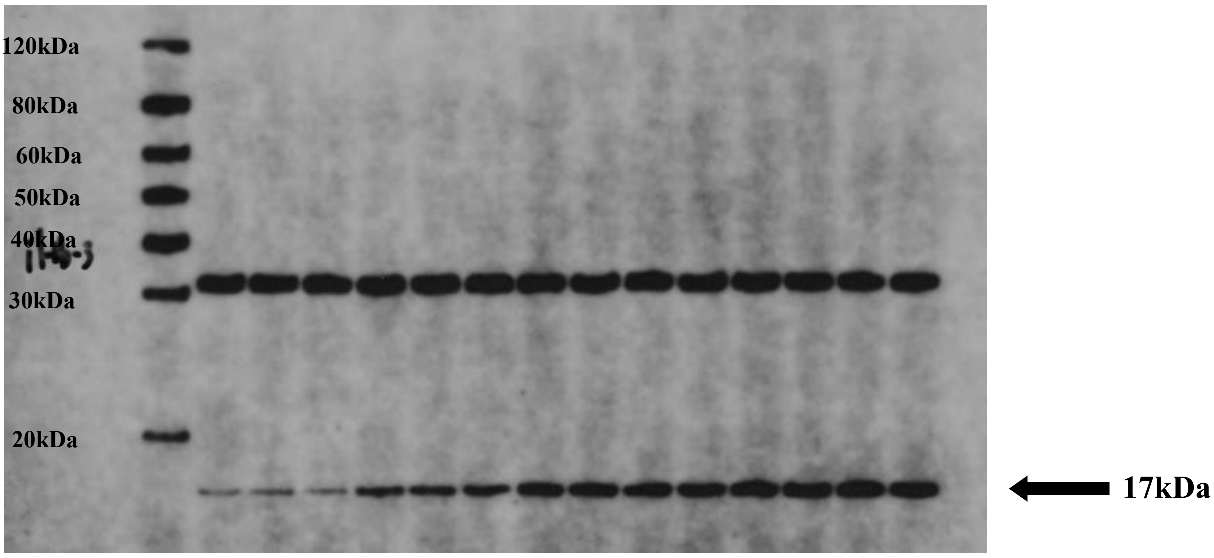

6.9 HAL-β-actin

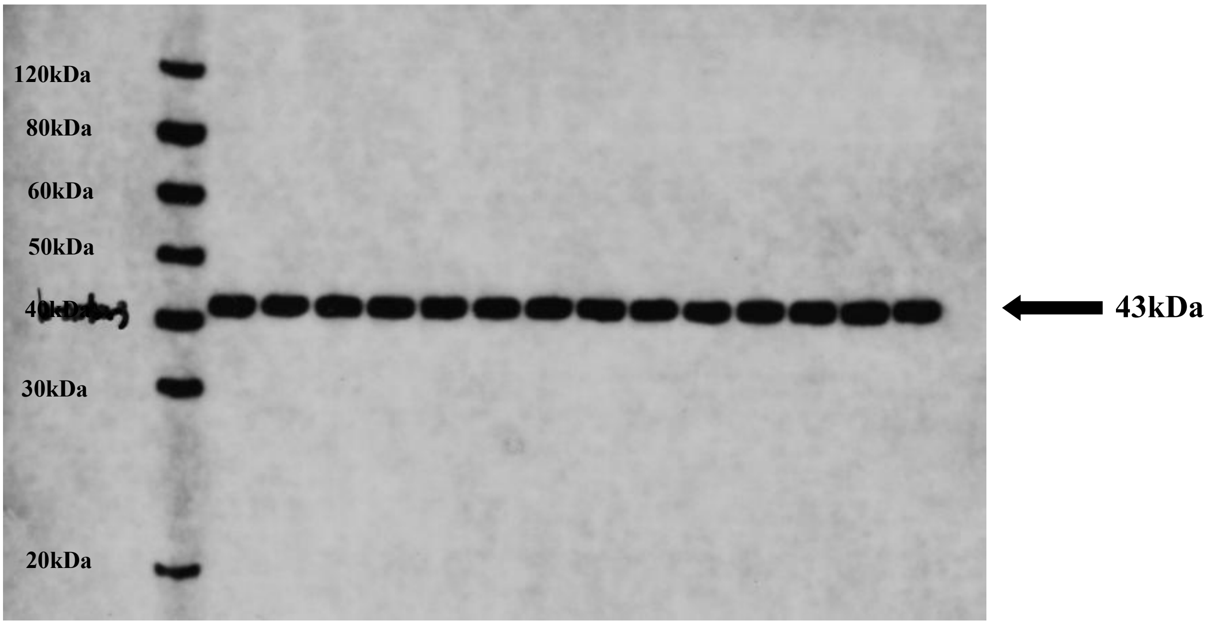

7. Supplementary figure 7. Original images in figure 6A and E

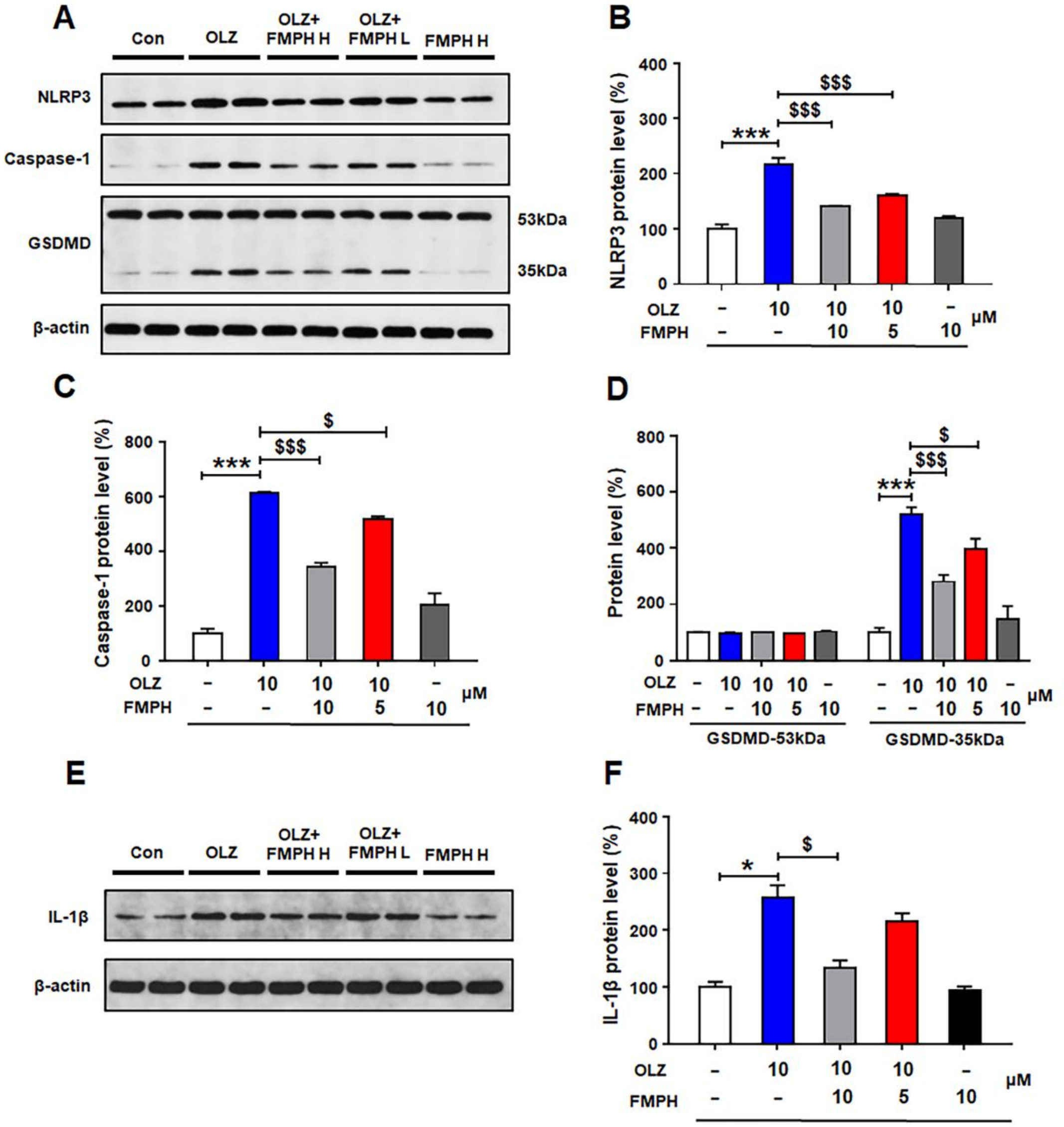

7.1 OLZ+FMPH-NLRP3

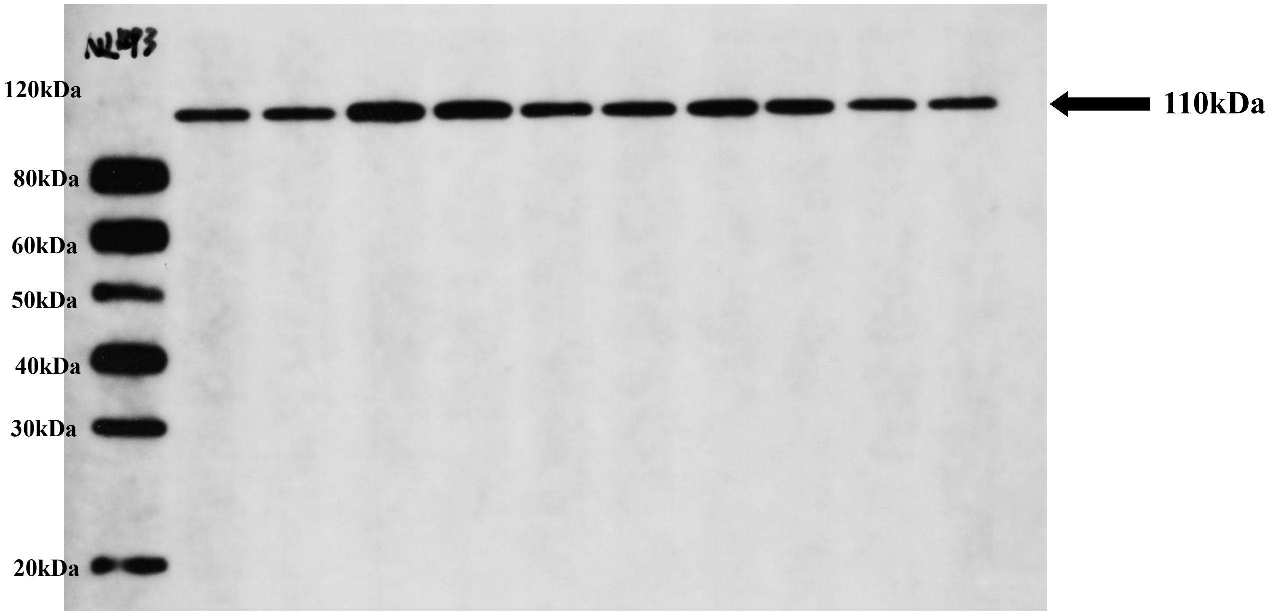

7.2 OLZ+FMPH-Caspase-1

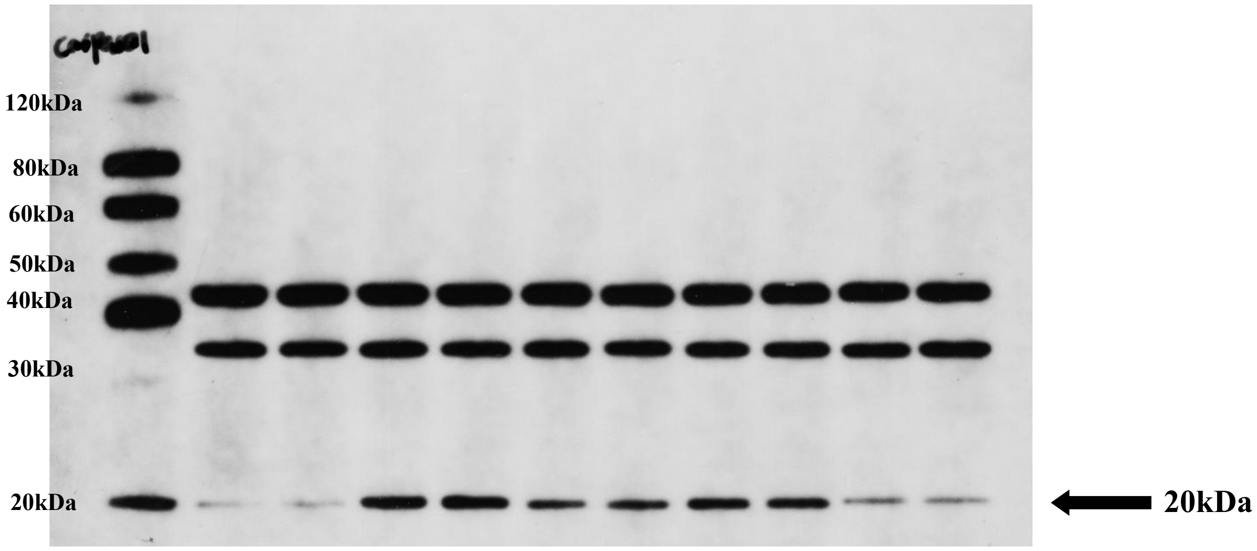

7.3 OLZ+FMPH-GSDMD

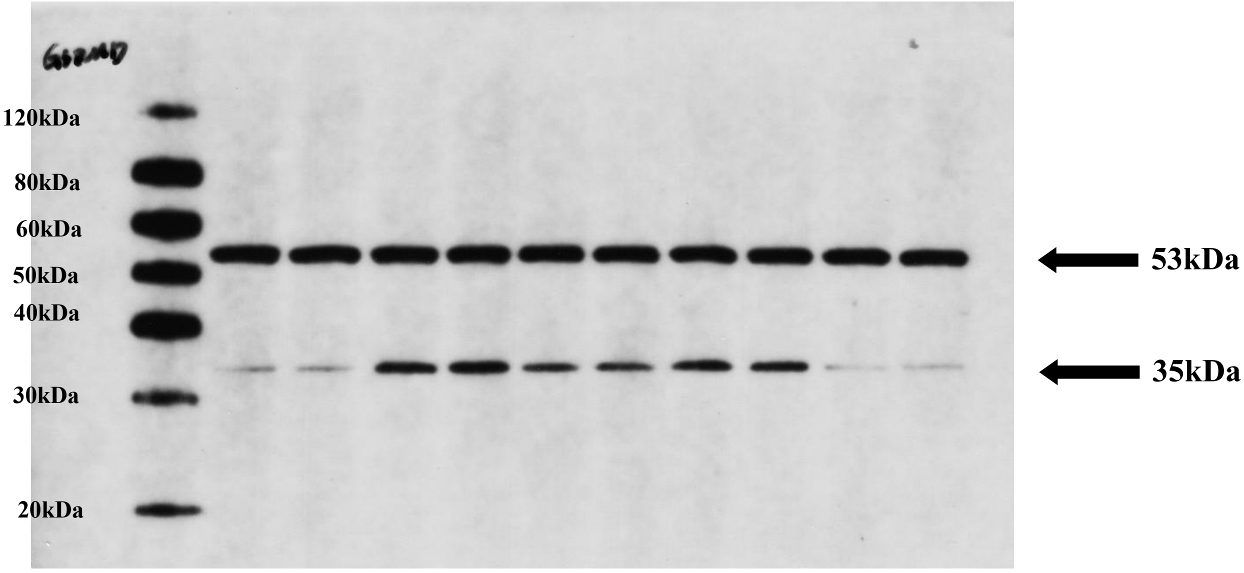

7.4 OLZ+FMPH-β-actin

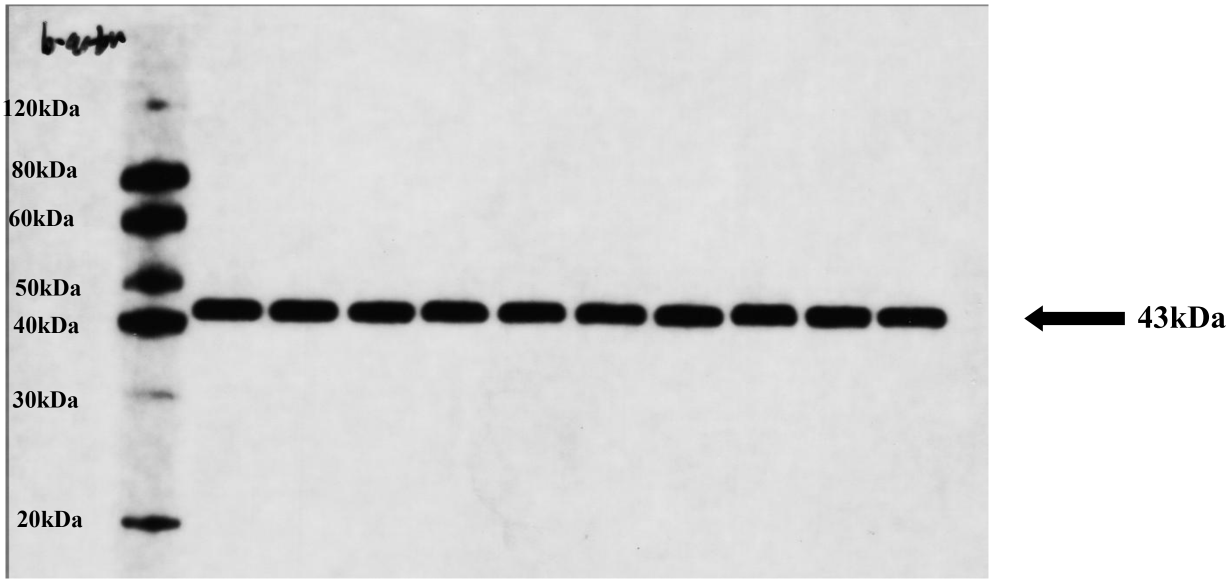

7.5 OLZ+FMPH-IL-1β

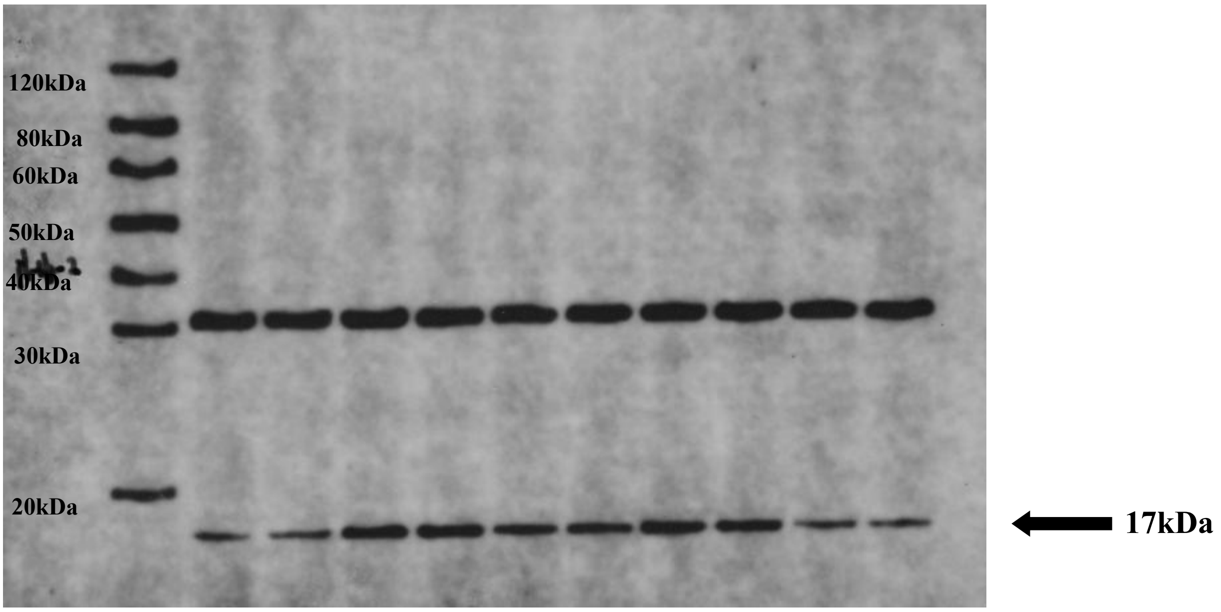

7.6 OLZ+FMPH-β-actin

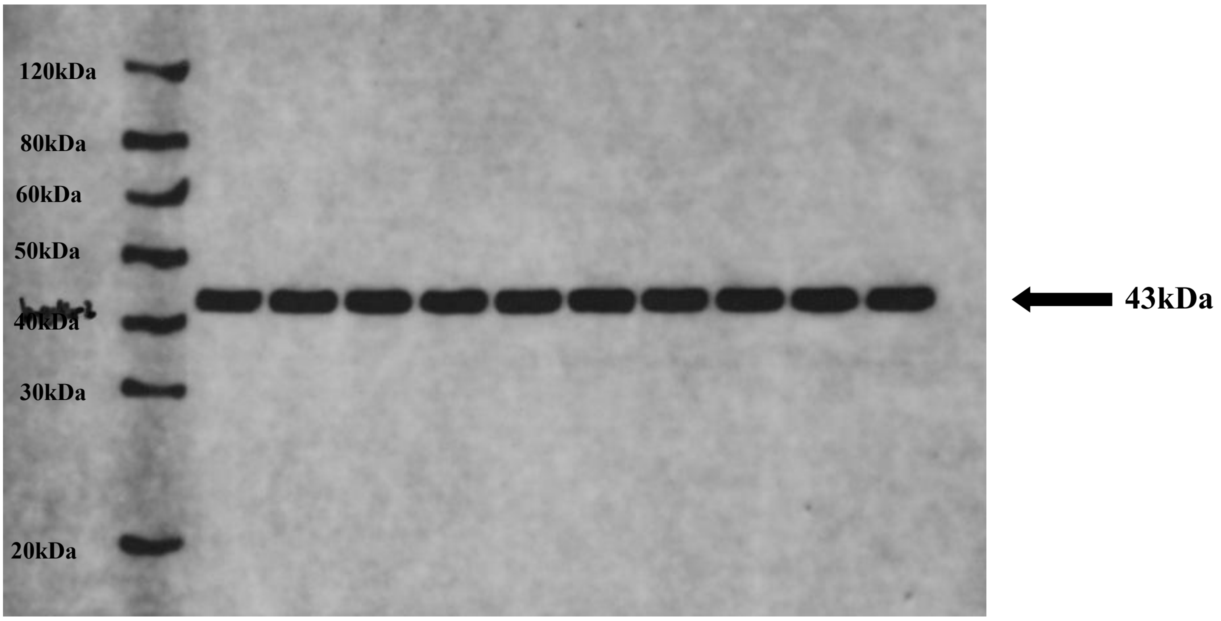

8. Supplementary figure 8. Original images in figure 7A and E

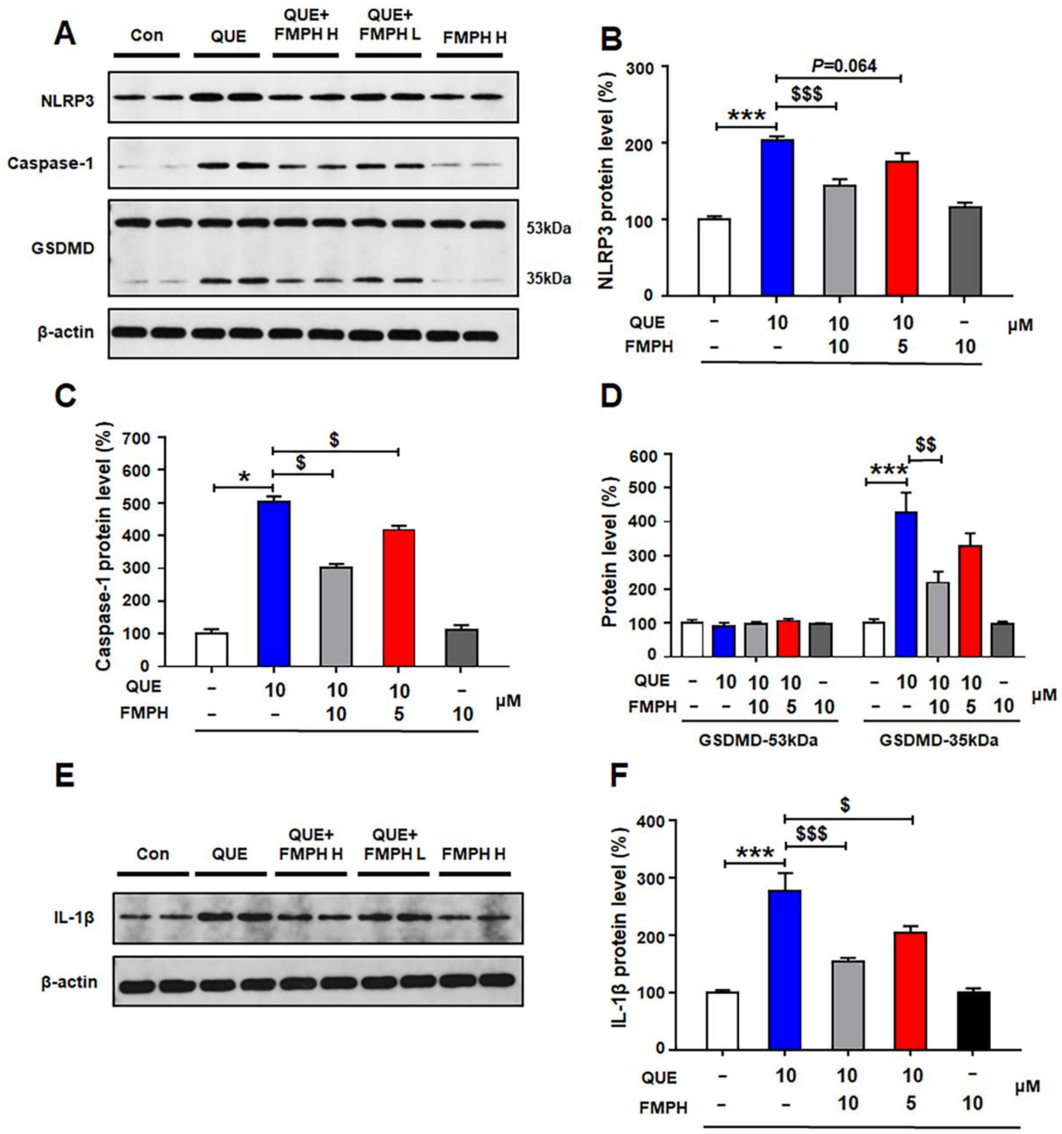

8.1 QUE+FMPH-NLRP3

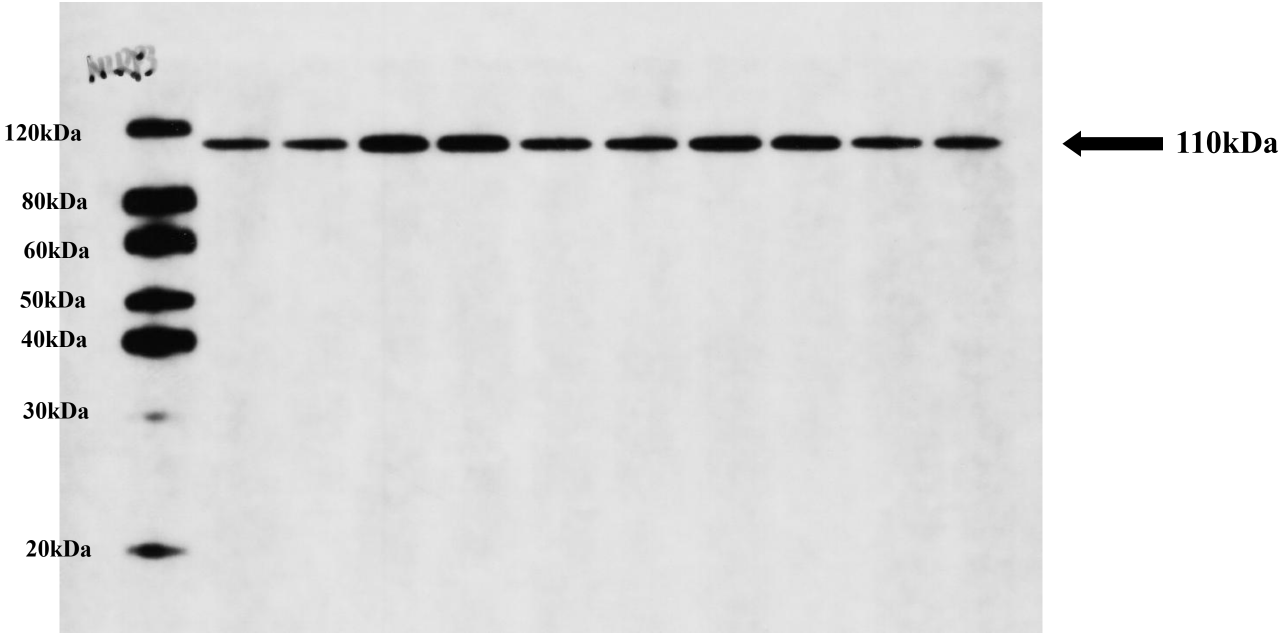

8.2 QUE+FMPH-Caspase-1

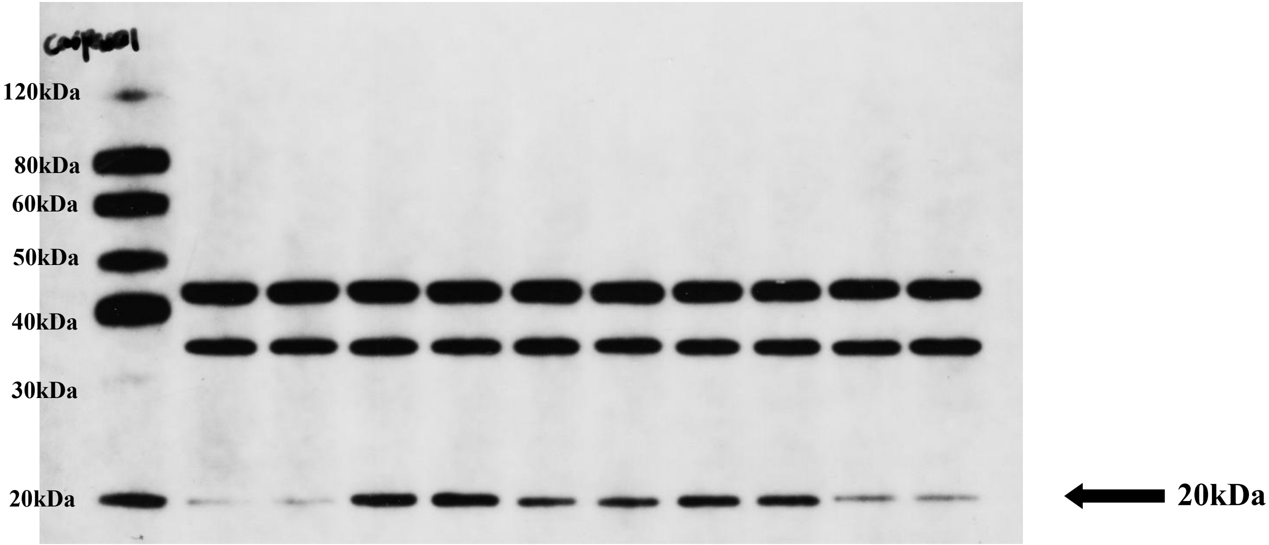

8.3 QUE+FMPH-GSDMD

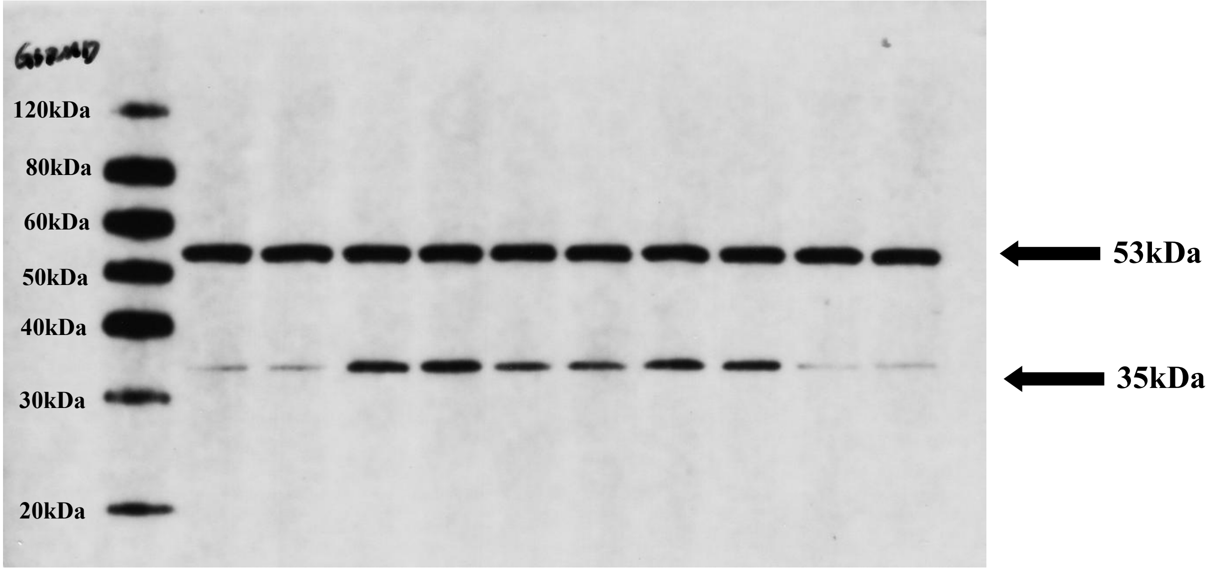

8.4 QUE+FMPH-β-actin

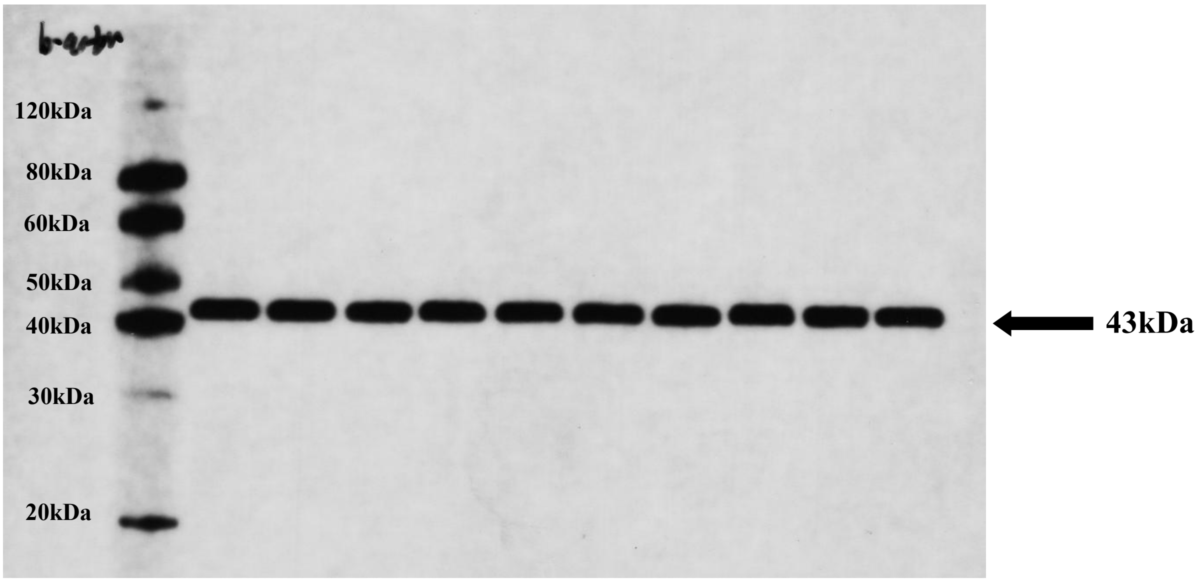

8.5 QUE+FMPH-IL-1β

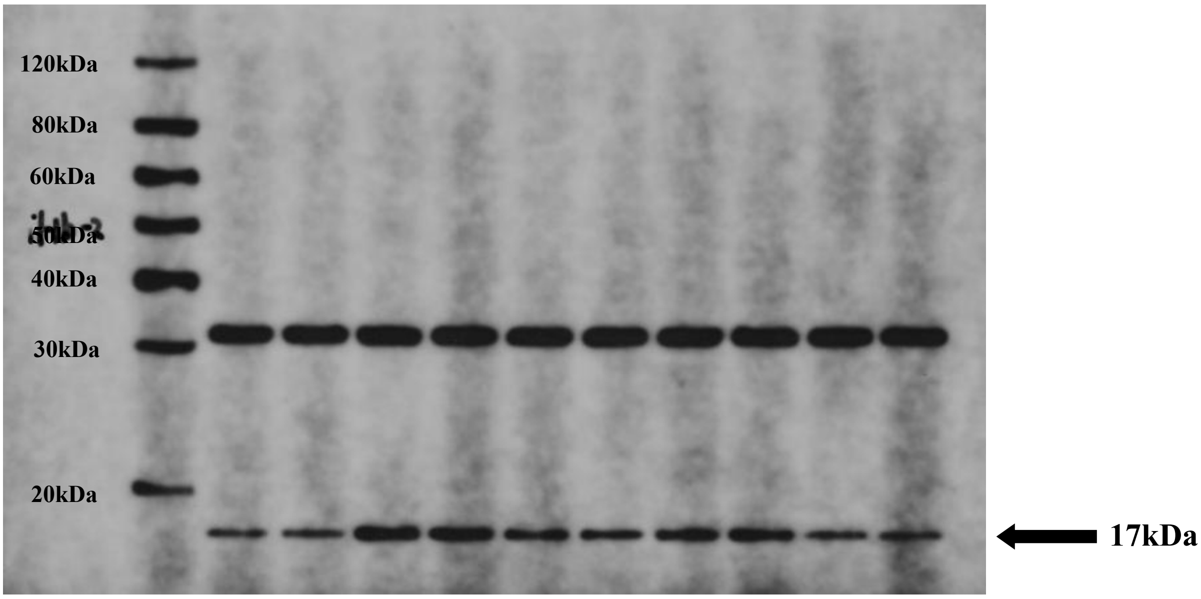

8.6 QUE+FMPH-β-actin

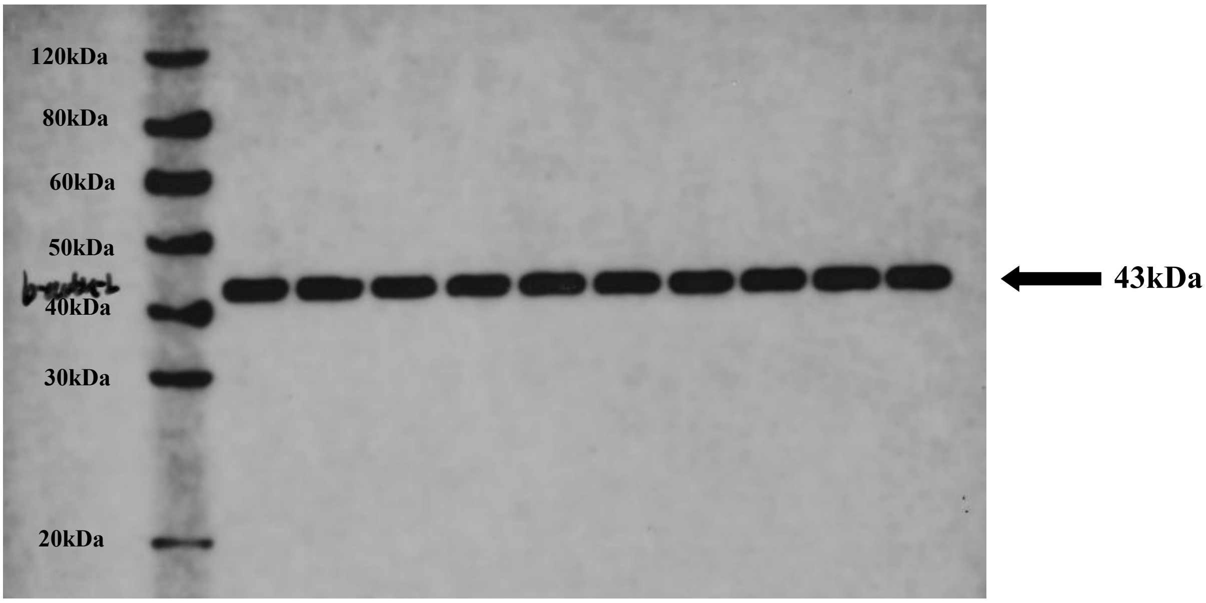

9. Supplementary figure 9. Original images in figure 8A and E

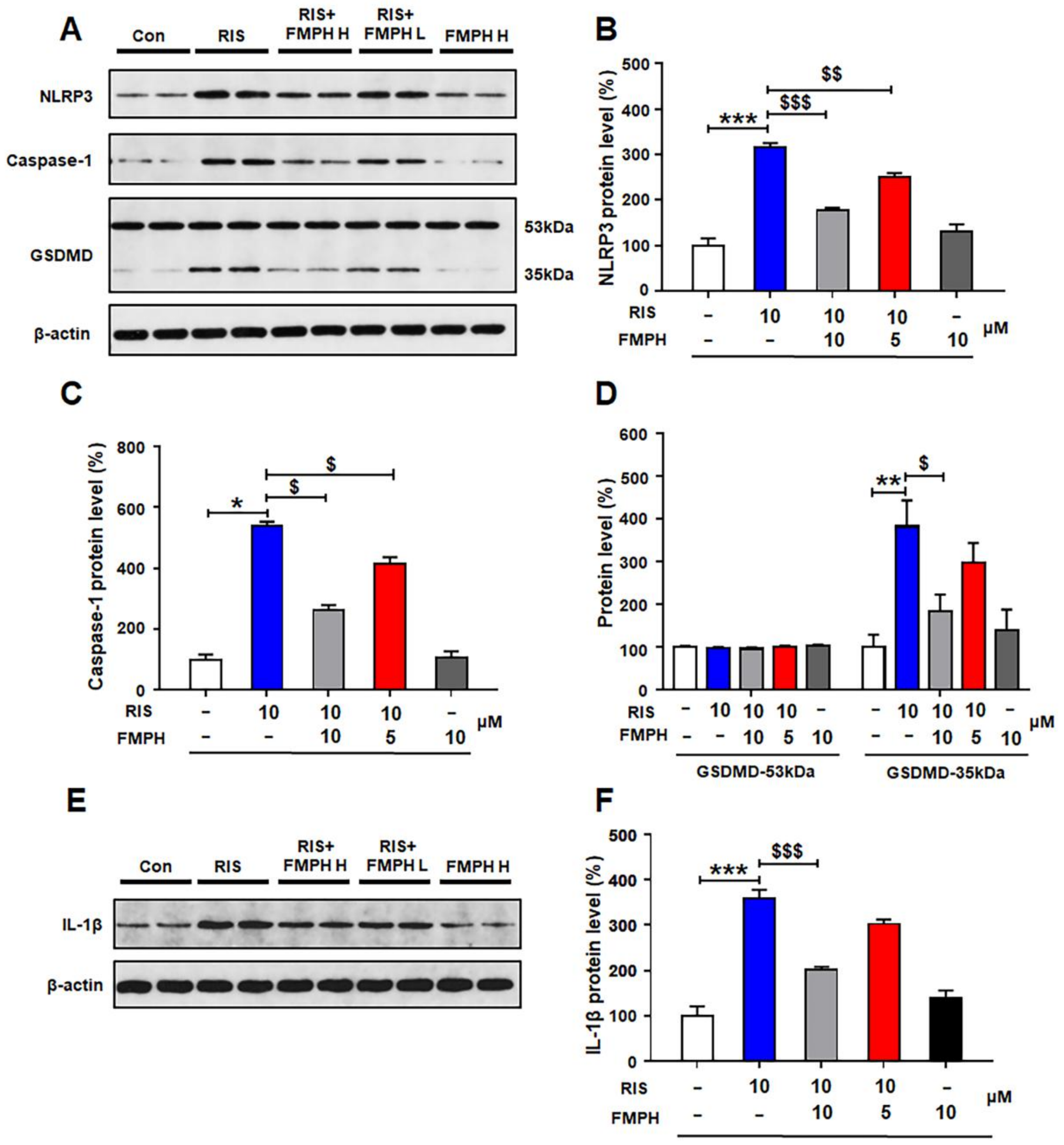

9.1 RIS+FMPH-NLRP3

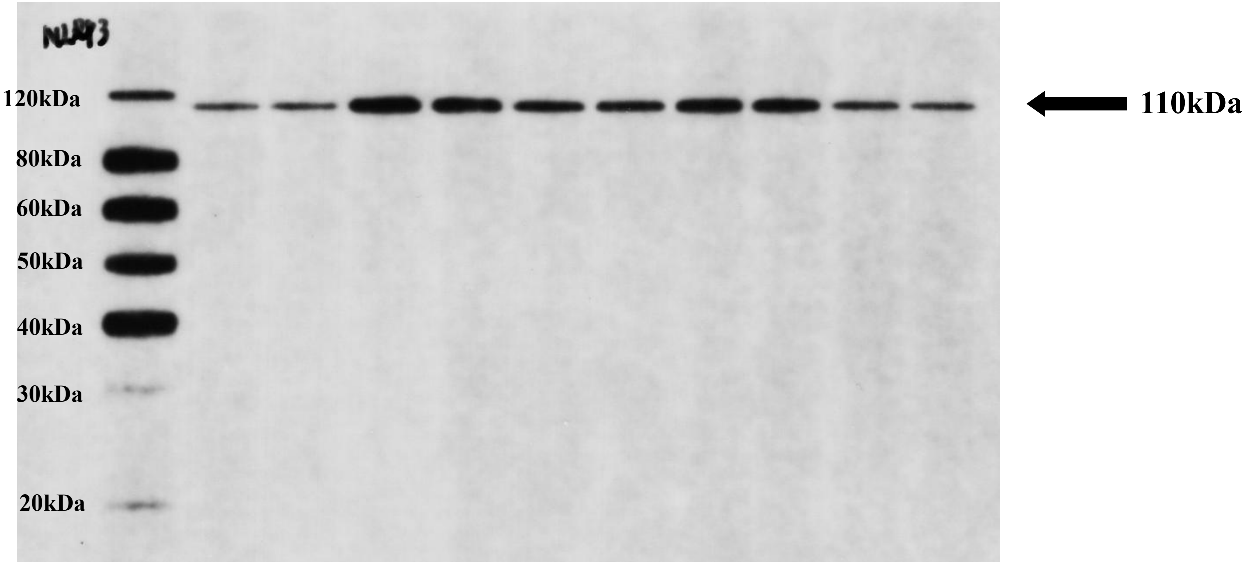

9.2 RIS+FMPH-Caspase-1

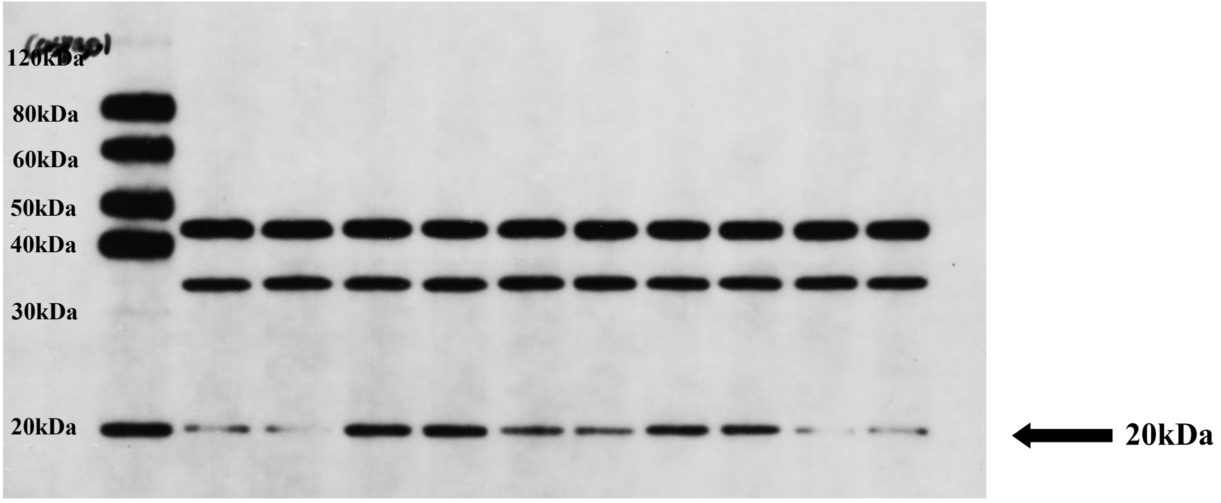

9.3 RIS+FMPH-GSDMD

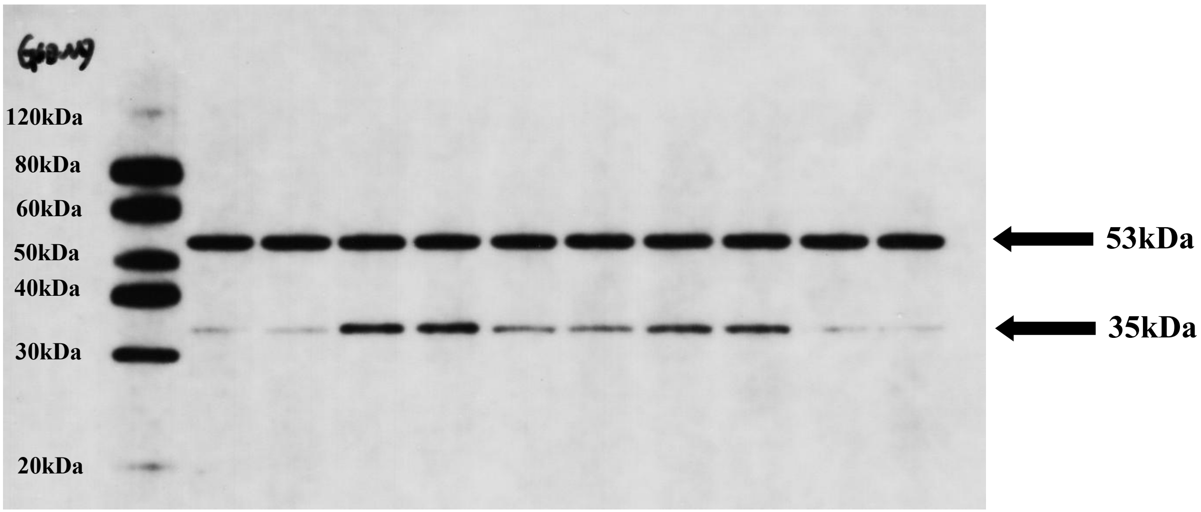

9.4 RIS+FMPH- β -actin

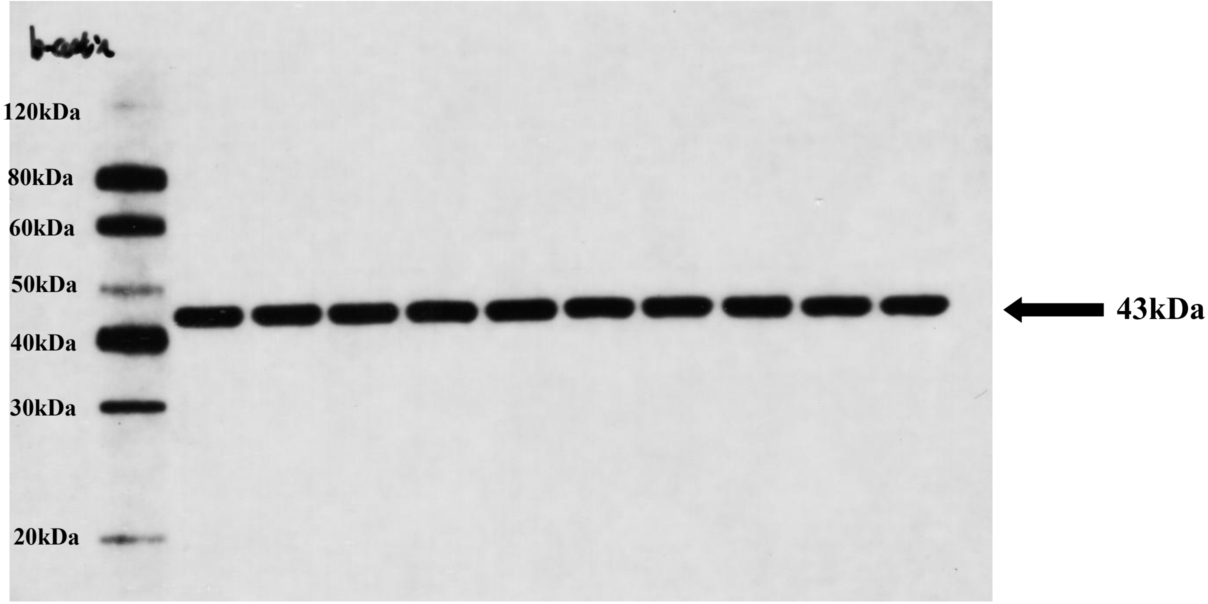

9.5 RIS+FMPH-IL-1β

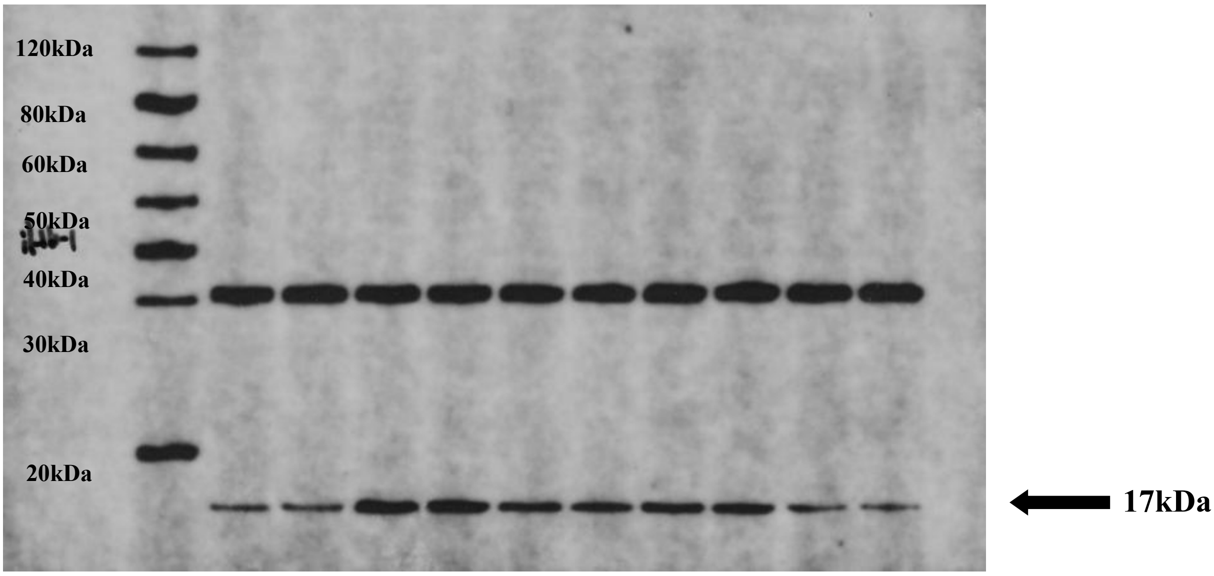

9.6 RIS+FMPH-β-actin

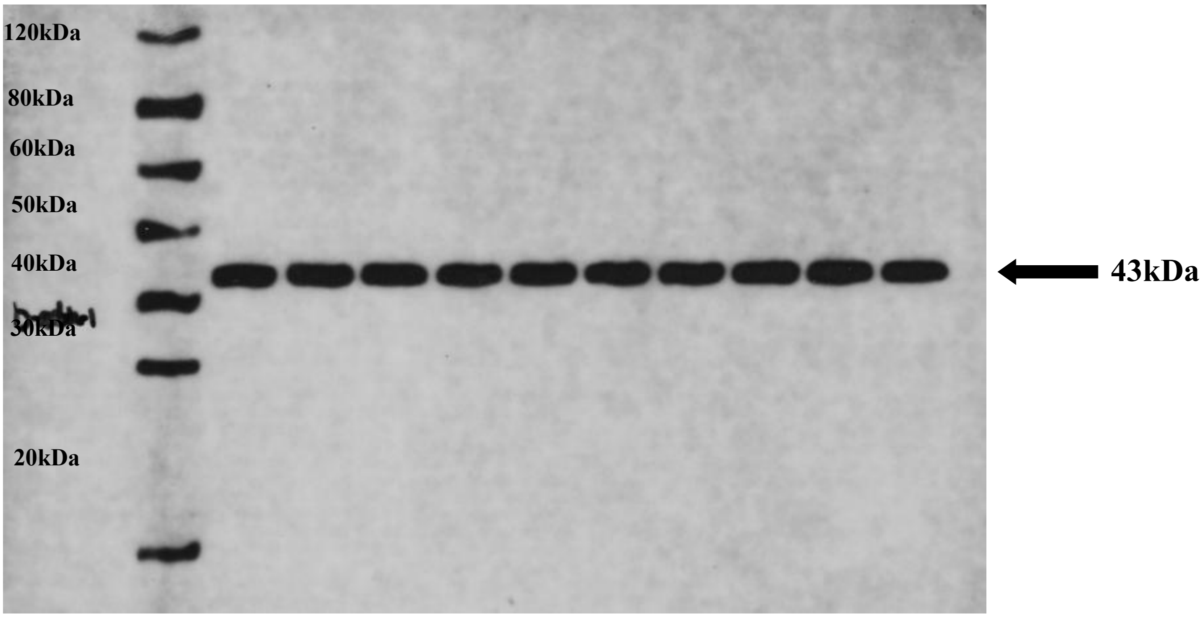

10. Supplementary figure 10. Original images in figure 9A and E

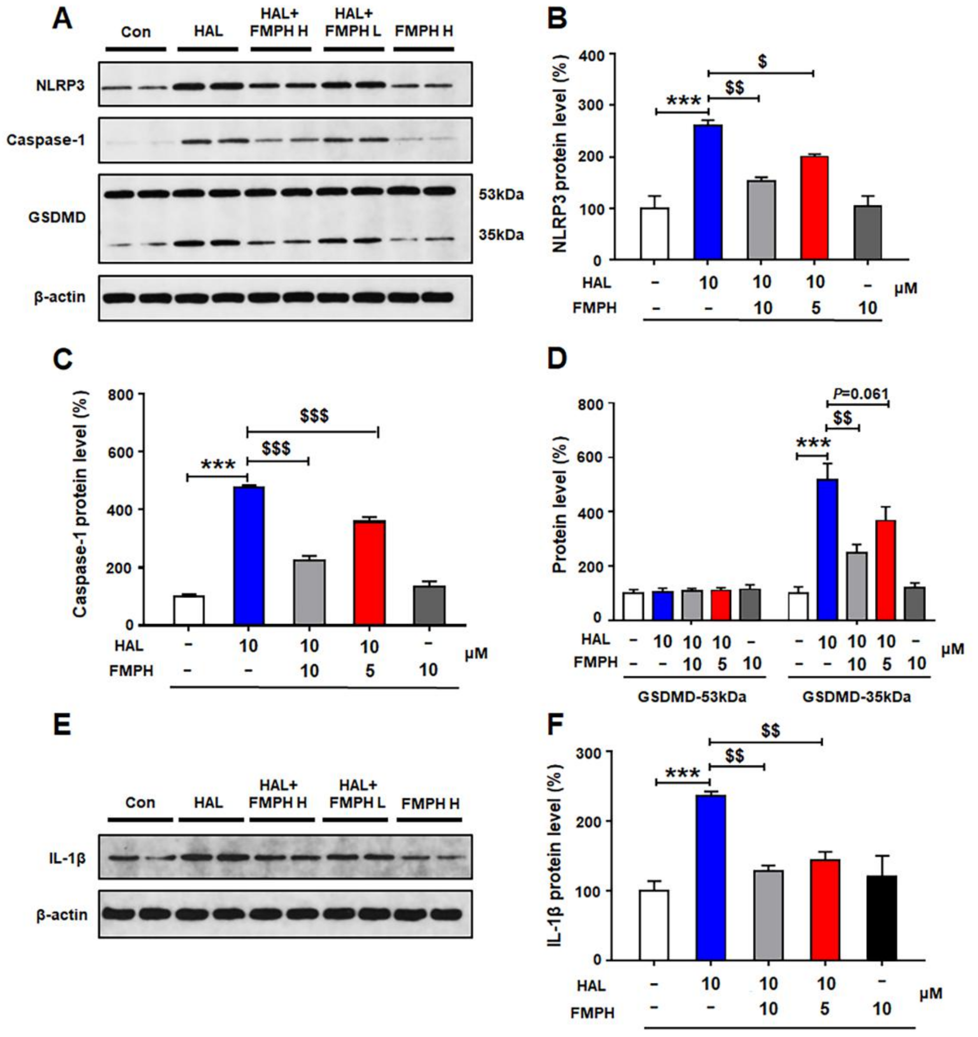

10.1 HAL+FMPH-NLRP3

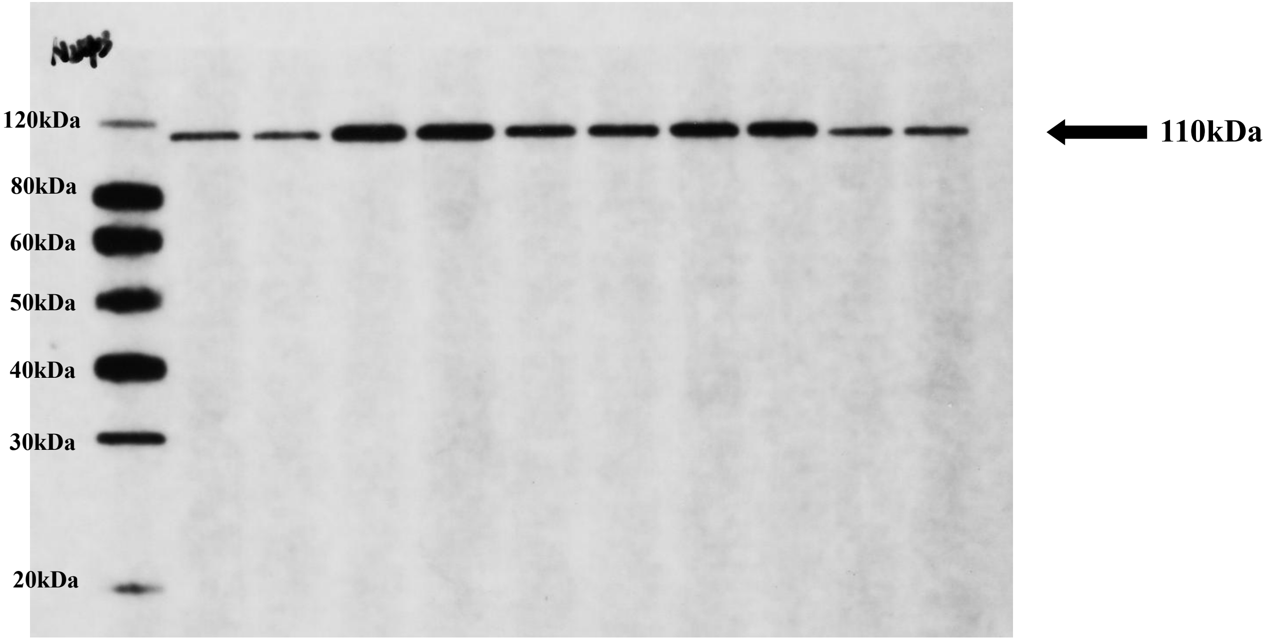

10.2 HAL+FMPH-Caspase-1

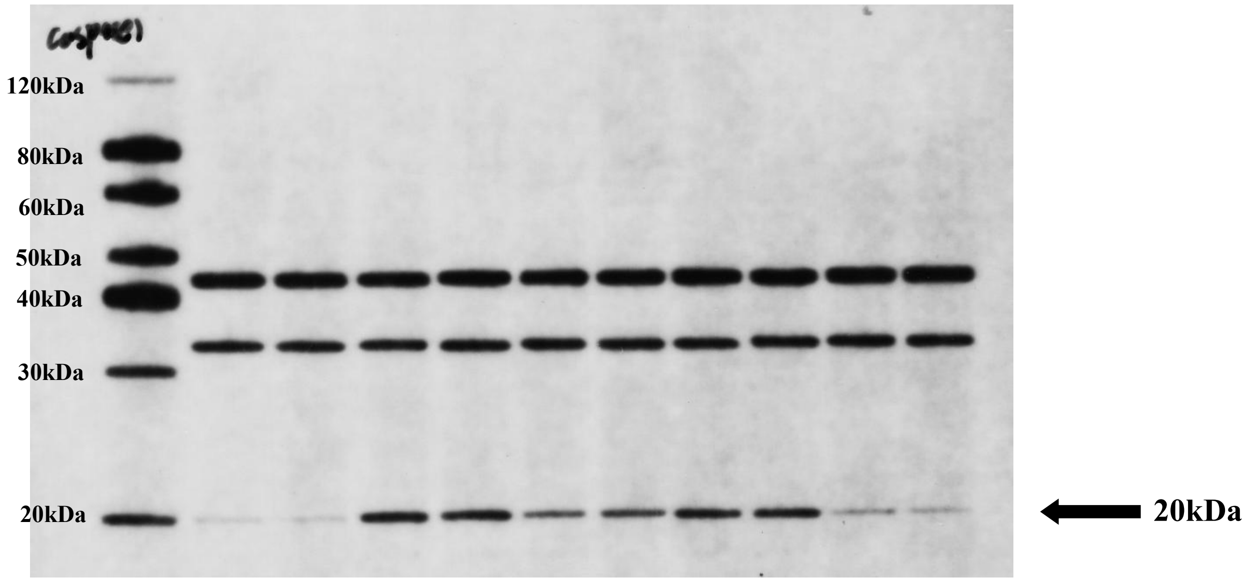

10.3 HAL+FMPH-GSDMD

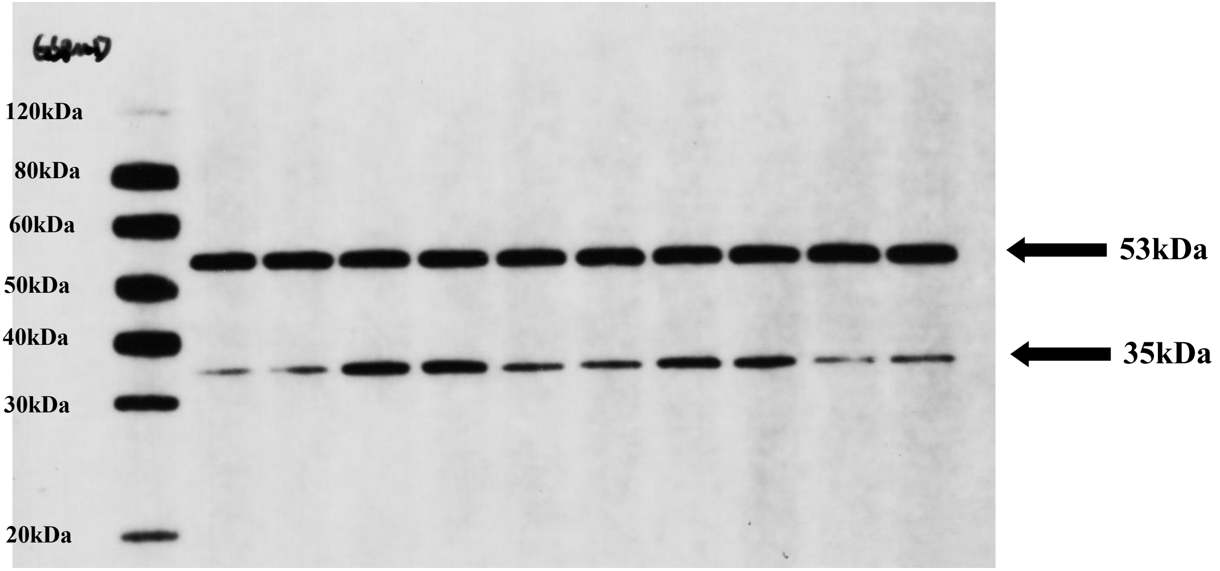

10.4 HAL+FMPH-β -actin

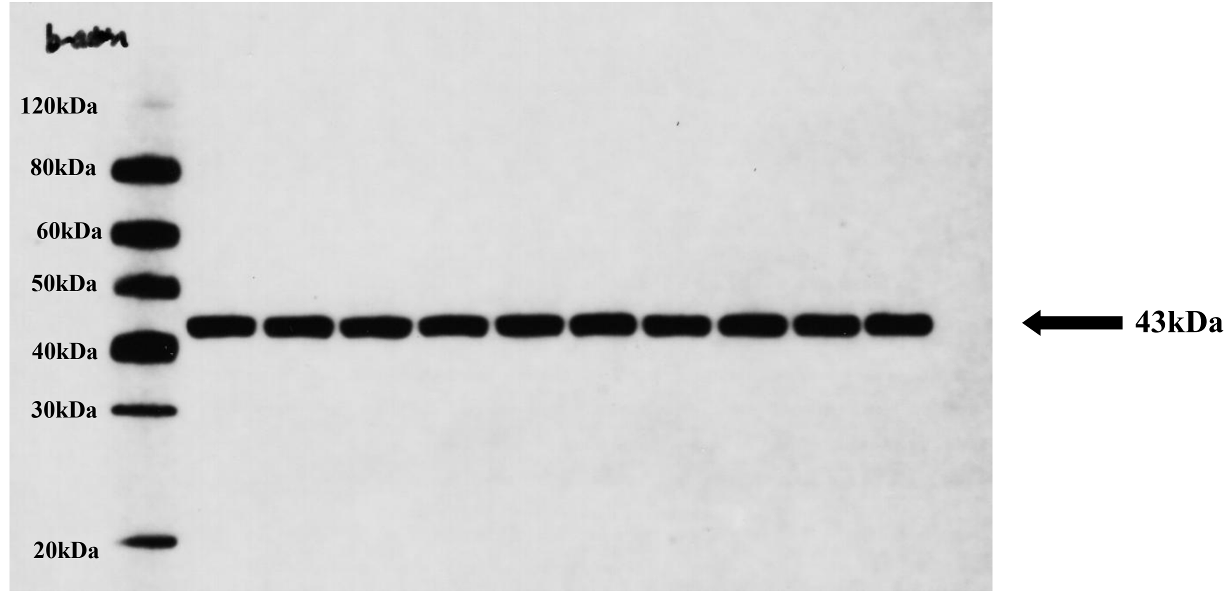

10.5 HAL+FMPH-IL-1β

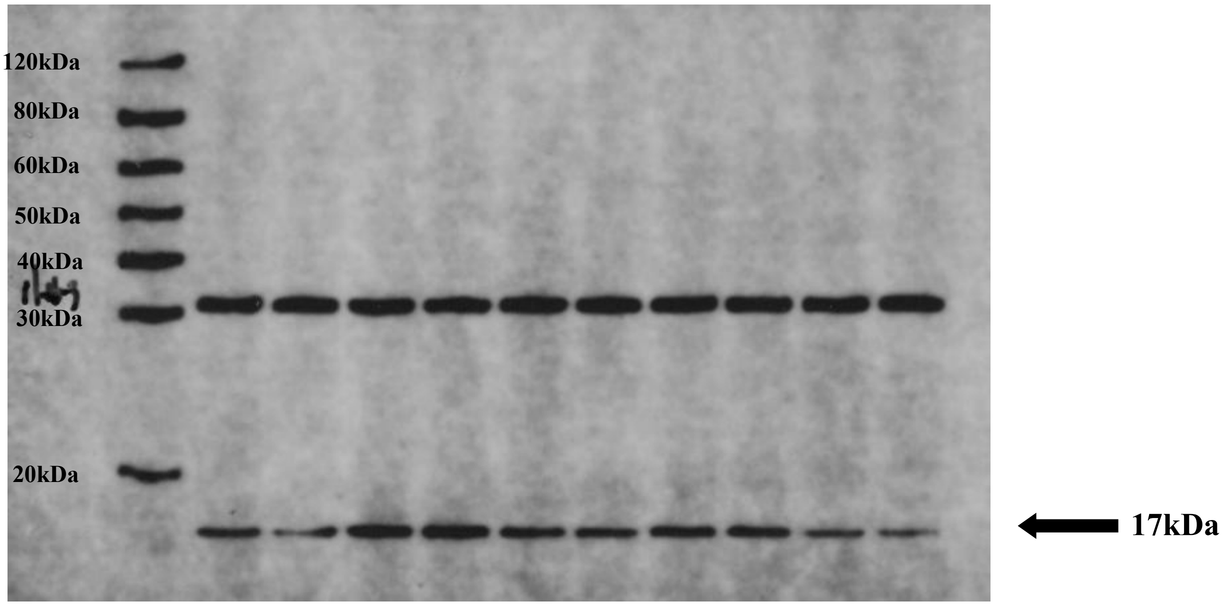

10.5 HAL+FMPH-β-actin

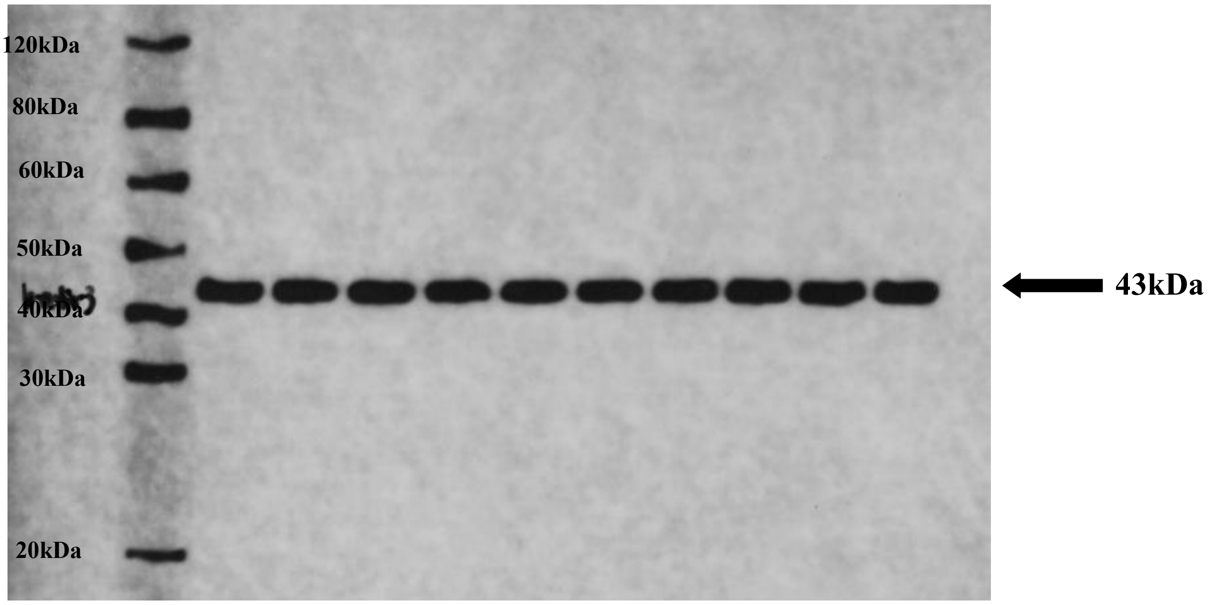

Supplement: Supplementary Figure 1 — Treatment with 1, 5, 10, or 20 μM 2-(3-trifluoromethylphenyl) histamine (FMPH) did not affect astrocyte viability. [file Data_Sheet_1.pdf]
